# Critical appraisal of fairness metrics for artificial intelligence-based clinical prediction models: a scoping review

**DOI:** 10.1016/j.landig.2026.101001

**Published:** 2026-05-28

**Authors:** João Matos, Ben Van Calster, Leo Anthony Celi, Paula Dhiman, Judy Wawira Gichoya, Richard D Riley, Chris Russell, Sara Khalid, Gary S Collins

**Affiliations:** Centre for Statistics in Medicine, Nuffield Department of Orthopaedics, Rheumatology and Musculoskeletal Sciences, University of Oxford, Oxford, UK (J Matos MSc, P Dhiman PhD, S Khalid PhD); Department of Development and Regeneration, KU Leuven, Leuven, Belgium (B Van Calster PhD); Leuven Unit for Health Technology Assessment Research (LUHTAR), Leuven, Belgium (B Van Calster); Beth Israel Deaconess Medical Center, Boston, MA, USA (L A Celi MD); Laboratory for Computational Physiology, Massachusetts Institute of Technology, Cambridge, MA, USA (L A Celi); Department of Biostatistics, Harvard T H Chan School of Public Health, Boston, MA, USA (L A Celi); Department of Radiology and Imaging Sciences, Emory University, Atlanta, GA, USA (J W Gichoya MD); Department of Applied Health Sciences, School of Health Sciences, University of Birmingham, Birmingham, UK (Prof R D Riley PhD, Prof G S Collins PhD); National Institute for Health and Care Research (NIHR), Birmingham Biomedical Research Centre, Birmingham, UK (Prof R D Riley, Prof G S Collins); Oxford Internet Institute, University of Oxford, Oxford, UK (C Russell PhD)

## Abstract

Predictive artificial intelligence (AI) offers an opportunity to improve clinical practice and patient outcomes but risks perpetuating biases if fairness is inadequately addressed. However, the definition of fairness remains unclear. We conducted a scoping review to identify and critically appraise fairness metrics in clinical predictive AI models. We defined a fairness metric as a metric quantifying whether a model discriminates (societally) against individuals or groups defined by sensitive attributes. We searched five databases for literature published during 2014–24, screened 820 records, included 42 studies, and extracted 63 fairness metrics. The search was limited to studies published in English. These metrics, which were classified by performance dependency, model output level, and base performance metric, revealed a fragmented landscape in the field of clinical predictive AI, with inadequate clinical validation and over-reliance on threshold-dependent metrics. 19 metrics, including only one metric for clinical use, were explicitly developed for health care. Our findings highlight conceptual challenges in defining and quantifying fairness and identify gaps in uncertainty quantification, intersectionality, and real-world applicability. Therefore, future works on clinical predictive AI models should prioritise clinically meaningful metrics.

## Introduction

Clinical prediction models (CPMs) are typically built using regression or machine learning methods, which are collectively known as predictive artificial intelligence (AI) methods.^1^ CPMs can be diagnostic or prognostic; diagnostic models estimate the probability that an individual currently has a condition (typically a disease), whereas prognostic models estimate the likelihood of an individual developing a clinical outcome over a specific time period.^2^ Predictive AI promises to improve patient outcomes and reduce health-care costs by informing clinical decision making and risk communication.^3^ However, despite CPMs being abundant in the biomedical literature,^4^ their effects on the real world remain suboptimal,^5^ with only a few exceptions such as FRAX^6^ and QRISK3.^7^

Several challenges hinder successful implementation of predictive AI models, including long-standing issues with respect to reporting quality^8,9^ and transparency. Such issues compromise model reproducibility and independent evaluation of model performance.^10^ Therefore, the TRIPOD guidelines were published in 2015 to provide minimum reporting recommendations for CPMs;^11^ in 2024, the TRIPOD guidelines were updated as TRIPOD+AI guidelines, encompassing AI methods.^12^ Further, design and methodological limitations such as small sample sizes and increased risk of overfitting affect robust model development,^13–16^ often resulting in suboptimal or unreliable model performance^17^ and poor generalisability to new settings.^18^

Algorithmic bias, arising when data or analysis biases are encoded directly or indirectly into a model during development, adds another layer of complexity to evaluating predictive AI performance.^19^ These biases can arise from non-representative data,^20^ different underlying disease distributions,^21^ existing health disparities,^22^ biases in medical devices,^23,24^ and other sources.^13^ Such biases are inherently dependent on the notion of sensitive (or protected) attributes, such as characteristics, variables, and dimensions, along which fairness can be evaluated. Such protected characteristics vary across geographical regions,^25–27^ and in medical contexts, some of them might reflect biological differences that legitimately influence health outcomes.^28,29^

Obermeyer and colleagues’ seminal work in 2019 shed light on the issue of fairness in clinical predictive AI.^22^ In this study,^22^ a model used to predict patients’ future health needs in the USA underestimated the needs of Black patients compared with those of White patients. This bias stemmed from the model’s use of health-care costs as a proxy for health needs, which did not account for systemic disparities in access to care.^22^ Deploying such models risks exacerbating existing health disparities,^30^ leading to unfairness.

### Defining fairness

Evaluating the performance of a CPM (eg, in terms of statistical discrimination, calibration, or clinical utility; table 1) typically focuses on population-level estimates (ie, averaged across individuals), which can mask potential differential model behaviour within that population. This masking of subgroup-level variation is also referred to as hidden stratification.^37,38^ Model performance, however, is expected to vary across subgroups. It is therefore important to understand the nature and magnitude of any differential model behaviour during model evaluation and identify signals suggesting unfairness.

A fundamental question that arises when evaluating potentially unfair CPMs is how to define fairness? In the context of predictive AI, fairness has been conceptualised in various ways, including group fairness,^42^ fairness through unawareness^43^ or awareness,^39^ counterfactual fairness,^35^ and minimax fairness,^44^ among others. Despite the abundance of fairness definitions,^45^ there remains a notable gap in a precise and widely accepted definition of a fairness metric.

### Motivation and aims

The incomplete understanding of fairness metrics hinders the development of specific definitions and recommendations in reporting guidelines, such as TRIPOD+AI,^12^ the Fairness, Universality, Traceability, Usability, Robustness, and Explainability-AI (FUTURE-AI) consensus guideline for trustworthy and deployable AI in health care,^46^ and the Standards for Data Diversity, Inclusivity, and Generalisability (STANDING Together) consensus recommendations.^30^ These guidelines address fairness considerations with caution and minimal specificity. Although previous notable efforts have reviewed fairness in machine learning,^36,47–51^ they rarely focus on CPMs or do not provide a comprehensive or critical perspective on fairness metrics in CPMs.^45,52,53^

Hence, we sought to identify and critically appraise fairness metrics reported in the CPM literature by addressing the following questions: first, which fairness metrics have been proposed, applied, and analysed in the CPM literature; second, how each metric should be interpreted in light of existing ethical and legal frameworks; and third, when the use of each metric is justified.

## Methods

We conducted a scoping review to identify fairness metrics used in the clinical predictive AI literature. The inclusion criteria and article selection process were kept intentionally broad to maximise coverage. We followed the methodological framework by Arksey and O’Malley^54^ and adhered to the PRISMA extension for Scoping Reviews (PRISMA-ScR) guidelines.^55^

For the purpose of this scoping review, we define fairness metric as a metric that quantifies the extent to which a model’s output does or does not discriminate (in the societal sense, based on a given notion of fairness) against individuals or groups defined by a sensitive attribute (table 1).^56^

### Search strategy and selection criteria

JM searched the literature published between Jan 1, 2014, and Oct 22, 2024, in PubMed, ACM Digital Library, IEEE Xplore, arXiv, and medRxiv databases to include journal publications, conference publication, and preprints. The search was limited to studies published in English. Four main keywords—namely, “fairness”, “metric”, “clinical”, and “model”—and their equivalent terms (“fair*”; “metric*”; “health*”, “clinic*”, “medic*”; “model*”, “algorithm*”, “machine learning”) were searched for in the title or abstract of articles. We deliberately excluded the terms “bias”, “parity”, and “disparity” from our search strategy owing to their ambiguity and broad use across different disciplines. Complete details of search strings for each database are previously published.^45^ Backward citation tracking was conducted throughout the review period from Oct 22, 2024, to May 1, 2025.

Non-duplicate research studies and review articles that directly engaged with fairness considerations in applied CPMs were included without restrictions on how fairness was addressed.

We excluded studies if the concerned metrics did not align with our definition of a fairness metric (table 1). We also excluded causal fairness notions (not necessarily the complete document) that require counterfactual predictions. Metrics that were devoid of sufficient detail to permit clear reporting of their definitions were also excluded. Finally, studies that did not propose or define fairness metrics were removed.

To reduce the risk of omission, we complemented the database search with backward citation tracking and manual reference screening to finally identify 63 distinct fairness metrics. These steps helped to include metrics from previous non-clinical reviews and those from new studies, relevant to CPMs. Backward citation searching was conducted after screening based on inclusion and exclusion criteria to maximise the identification of relevant fairness metrics and to obtain additional details on their definitions. This approach enabled the inclusion of both review articles and original studies that proposed, defined, or applied fairness metrics, although these metrics were not originally developed for health-care contexts.

### Data extraction: metric information

For each metric, JM extracted the formula and all information required for its computation. In case of studies proposing families of metrics, we report specific representative examples as applicable (eg, equity-scaled area under the receiver operating characteristic curve [AUROC] for the family of equity-scaled metrics^57^). Metrics with ambiguous names were standardised to ensure consistency and alignment with the information in the literature. For example, the metric discrimination index was redefined as F1-score parity after reviewing the implementation details of both metrics.^58^

We categorised all metrics based on their proposed domain (health care or others) and their positions within existing fairness taxonomies.^1,31,36,39^ We also identified alternative names of metrics and the required inputs for their computation (eg, type of model output, outcome labels, or predictor conditioning). Further, we examined the types of sensitive attributes (binary, multigroup, or continuous) that each metric supports. We also assessed interpretation aspects, including bias-preserving versus bias-transforming properties,^1,31,36,39^ outcome prevalence dependency, and target fairness values.

### Data extraction: taxonomy of fairness metrics

Multiple and conflicting fairness taxonomies (n=12)^45^ exist in the literature. The figure presents the taxonomy adopted in this scoping review for fairness metrics. The adopted taxonomy builds on previous taxonomies and can serve as a guide in selecting metrics according to the available information and fairness objectives.

Evaluation of fairness begins with consideration of performance dependency—that is, whether fairness should be evaluated with respect to model performance (performance-dependent or supervised fairness evaluation) or not (performance-independent or unsupervised fairness evaluation, with no consideration of outcome labels or ground truth labels). This distinction between the evaluation approaches hinges on the interpretation of fairness and validity of the labels used to develop and evaluate the model. If these outcome labels are accepted as a fair representation of the target population, the use of a performance-dependent metric is justified. Conversely, if the labels risk encoding health inequities that the model should not reinforce (eg, disparities in access to care), performance-independent metrics are more appropriate. In practice, what is being inquired is whether the status quo reflected in the data and historical outcome labels are acceptable as a reference (figure).

The second consideration is whether fairness should be assessed at the level of estimated probability $\left(\hat{\text{P}}\right)$ or the predicted class $\left(\hat{\text{Y}}\right)$. Probability-based metrics (eg, mean score parity^59^) assess fairness without applying a decision threshold. In contrast, threshold-dependent metrics (eg, statistical parity^60^) evaluate fairness at the predicted class level; the application of threshold-dependent metrics thus depends on applying a threshold to convert the estimated probability into class prediction. This distinction has also been described in a preprint published online in 2025 as approaches that use “informing with a risk score” (probability-based evaluation) versus “decision support with a classification” (threshold-dependent evaluation).^61^

The third consideration is the type of performance metric. If a performance-dependent fairness metric is chosen, the focus shifts to what type of model performance^1^ should be compared across groups.

Probability-based metrics focus on calibration (eg, calibration-in-the-large parity^62^), discrimination (eg, AUROC parity^63^), or overall performance (eg, Brier score parity^64^), whereas threshold-dependent metrics focus on partial metrics (eg, equality of opportunity difference^42^), summary metrics (eg, accuracy gap^65^), or clinical utility (eg, subgroup net benefit^66^). Following prior taxonomies, fairness metrics were also categorised as individual^39^ or group fairness metrics^36^ (table 1).

### Data extraction: qualitative critical appraisal

We developed a data extraction form^45^ to critically appraise each metric by assessing its suitability, limitations, and potential pitfalls in clinical scenarios. The appraisal process included outlining its justified use. We provide overall critical appraisal and guidance for different groups of fairness metrics. To aid interpretation, we categorised our recommendations into three levels of guidance: recommended if, referring to metrics that are generally suitable and align with clinical and ethical standards under specific conditions; use with caution, referring to metrics that might be relevant but require careful contextual consideration and are not essential; and inadvisable, referring to metrics with substantial limitations or risk of harm that outweigh potential benefits in most scenarios.

## Results

927 documents were identified through database searches. After removing duplicates and ineligible records based on language and format, 820 records were screened for inclusion. 644 articles were excluded for not focusing on CPMs, not addressing fairness, or being devoid of any fairness metric. The remaining 176 reports were sought for retrieval, with 157 further excluded during data extraction owing to the absence of fairness metric proposals, definitions, or relevant applications. Finally, 19 reports met inclusion criteria, and an additional 23 reports were identified through backward citation searches, resulting in a total of 42 articles (three reviews and 39 research articles) describing fairness metrics relevant to CPMs.^45^

Across the 42 papers, we identified 63 fairness metrics that aligned with the definition proposed in this Review (table 1). A comprehensive and detailed formalisation (including mathematical formulation) of each metric have been previously published.^45^

Fairness metrics from most articles (20 of 42) originated from AI rather than from biomedical (14 of 42) or applied ethics (eight of 42) research. Only 19 of the 63 metrics were explicitly defined for health-care applications.

### Metrics identified

Among the identified performance-independent metrics (15 of 63, including that reported in a preprint^67^),^48,60,67–73^ most (n=13) were group fairness metrics, and only two were individual fairness metrics. The performance-independent metrics were further divided into probability-based (n=6, including mean score parity^59^) and threshold-dependent (n=7, including statistical parity^60^). However, only three of these 15 metrics were proposed for health-care applications.

Performance-dependent metrics (n=48, including those reported in preprints^66,74^)^42,51,57,58,62–66,69,74–96^ were substantially more common than performance-independent metrics, and all studies associated with performance-dependent metrics focused on group fairness. Only 16 of these 48 metrics were explicitly proposed for use in health-care contexts. The performance-dependent metrics were also divided into probability-based (n=22) and threshold-dependent (n=26) metrics. Most of the probability-based performance-dependent metrics focused on discrimination (n=12, including AUROC parity^63^); these metrics were followed by calibration-based metrics (n=5, including expected calibration error parity^78^) and metrics that focused on overall performance (n=5, including Brier score parity^64^). Threshold-dependent performance-dependent metrics primarily assessed fairness using confusion matrix-derived metrics, with most of them focusing on partial metrics (n=18, including equal opportunity difference^42^), followed by those focusing on summary metrics (n=7, including overall accuracy gap^65^). Only one threshold-dependent performance-dependent metric that focused on clinical utility (subgroup net benefit^66^) was identified (table 2). Most of the metrics identified (61 of 63) were group fairness metrics. Individual fairness metrics were notably scarce (two of 63).

A structured catalogue of fairness metrics, including classification information within the fairness taxonomies, operationalisation details, interpretation and implications, and justified use and critical appraisal, is summarised.^45^

### Critical appraisal of identified fairness metrics

Based on the catalogue of fairness metrics, we critically appraised the metrics in terms of their applicability, interpretability, quality of definition, validation, and alignment with clinical and ethical considerations (table 3). Table 4 lists examples of metrics that are currently used in real-world scenarios. We summarise key qualitative findings for each category, providing guidance on which metrics to prioritise when assessing the fairness of CPMs.

Performance-independent metrics typically aim to ensure parity in the model’s positivity rates.^31^ For these metrics, a perfectly accurate model (ie, with no classification errors at the population level after finalising a decision threshold) might never achieve complete fairness if prevalence differs across groups defined based on the sensitive attribute.^102^

Probability-based performance-independent metrics (eg, mean score parity^59^) should be used with caution as they are challenged by their reliance on mean values without a defined metric of uncertainty. Metrics such as unsupervised ranking fairness^69^ might be of value if some form of unsupervised discrimination analysis was deemed necessary; however, these metrics are inadvisable without further empirical evaluation, as their theoretical foundation in health care remains unclear with no proper validation.

Threshold-dependent performance-independent metrics such as statistical parity,^60^ children metrics,^68,72^ and disparate impact^73^ can be useful for assessing fairness at the predicted class level, without relying on the quality of existing labels. Conditional statistical parity^48^ metric is especially relevant when legitimate factors (such as comorbidities or biological differences) justify group-specific, differential risk estimates. The disparate impact metric has been connected with the US disparate impact law^73^ despite the fact that the four-fifths rule that is often associated with this metric is neither necessary nor sufficient to abstract the disparate impact law;^112^ in contrast, the conditional statistical parity metric aligns with the EU non-discrimination law, which could be desirable from a legal perspective.^97^ However, operationalising fairness remains inconsistent when sensitive attributes are non-binary or require conditioning across multiple categorical strata. Although survival^70^ and censoring-based^71^ metrics extend fairness evaluation to more complex scenarios than standard prediction settings without time-to-event outcomes, the adoption of these metrics is hindered by insufficient empirical evaluation and understanding. Similarly, metrics such as differential fairness^68^ are not supported by adequate empirical evidence and are currently inadvisable for real-world use.

Performance-dependent metrics compare performance across individuals or subgroups of individuals. According to these metrics, a model is perfectly fair when it achieves, for example, consistent (but not necessarily perfect) discrimination, calibration, or clinical utility across subgroups. These metrics assume that the distribution of the predictors and outcomes present in the model development data reflect the nature of target population. By prioritising consistency in model performance (ie, parity across individuals or groups), the status quo in health-care delivery is preserved and labels are reinforced.^31^ Whether statistical parity should be prioritised in fairness evaluation remains debated; some researchers argue that model performance can naturally vary between subgroups and that the focus should instead be on ensuring minimum acceptable performance for all subgroups.^102^

Probability-based performance-dependent metrics assess disparities in model performance across subgroups using base performance metrics that are probability-based and do not apply thresholds. These metrics are often preferred for CPMs because they evaluate model performance independently of threshold choices that might differ across subgroups or clinical settings.^1^

Among probability-based performance-dependent metrics focusing on discrimination, AUROC parity,^63^ which was built on a previous study^1^ is recommended for quantifying discrimination disparities. Although AUROC, with its value remaining unchanged after rank-preserving transformation of estimated probabilities, is a semi-proper metric, it remains useful as it focuses on discrimination performance. In contrast, area under the precision–recall curve parity^64^ metric is inadvisable owing to its semi-proper scoring nature, absence of focus (mixing discrimination and clinical utility),^1^ and tendency to favour groups with higher outcome prevalence.^98^ Extensions such as xAUROC,^76^ xAUROC disparity,^76^ sAUROC,^77^ pairwise ranking fairness,^69^ mean performance-scaled disparity AUROC (reported in a preprint^74^), equity-scaled AUROC,^57^ concordance imparity,^79^ and AUROC parity ratio^80^ are not backed by any clear motivation, sufficient implementation details, or in-depth clinical validation, making them challenging to interpret and inadvisable for use in the time being.

Among probability-based performance-dependent calibration metrics, equal calibration^82^ and well calibration^51^ are vaguely defined in the literature and should be used with caution, particularly as no clear implementation details exist on how to consider multiple thresholds. Nevertheless, such metrics that satisfy the notion of equal calibration across groups are (in principle) desirable.^99^ Expected calibration error parity,^78^ calibration-in-the-large parity,^62^ absolute calibration error parity,^81^ and other similar metrics should also be used with caution. Although such metrics can be informative to assess calibration, their use should be accompanied by a calibration plot for each subgroup and be paired with discrimination metrics. Furthermore, despite being proper metrics,^1^ expected calibration error parity and absolute calibration error parity metrics have been criticised for being dependent on the way in which calibration binning is performed and for handling overestimation and underestimation in a similar manner.^100^ Behaviour of fairness metrics focusing on differences in expected calibration error parity or absolute calibration error parity is not well studied.

Among probability-based metrics that focus on overall performance, Brier score parity^64^ and log-loss parity^81^ metrics should be used with caution. Despite being proper metrics (table 1) of model performance,^1^ these two metrics offer restricted interpretability and provide only an overall assessment of the model, blending elements of discrimination and calibration.^1^ Balance for positive class and balance for negative class metrics^82^ should also be used with caution because they are not studied for use in CPMs. The Earth mover’s distance for equivalent separation,^64^ which compares the distributions of predicted outcome probabilities using the Wasserstein distance, can be conceptually informative as it seeks to satisfy the separation non-discrimination criterion of fairness^36^ in its strongest form. Although its behaviour in CPMs is expected to be appropriate, the metric is not empirically evaluated.

Threshold-dependent performance-dependent metrics compare classification performance across groups. These metrics are inherently constrained because they rely on a decision threshold that might be chosen with no clinical rationale. Consequently, the reported quantity (eg, accuracy) is specific to the chosen threshold and might not be generalisable for other metrics. Moreover, some of these metrics are improper when considered alone.^1,98^

Among threshold-dependent performance-dependent metrics focusing on partial metrics, equal opportunity difference^42^ and predictive parity,^87^ which aim to achieve parity in true-positive and false-positive rates, can be relevant if reported together. Similar considerations apply to predictive parity^87^ and negative predictive value parity.^75^ These metrics have the advantage of being easily interpretable; however, their reliance on potentially arbitrary thresholds restricts standalone use. Equalised odds^42^ is inadvisable because combining it with true-positive and false-positive rates makes the metric lose focus, hampering a clear interpretation. Worst-group recall^91^ and maximum difference recall^89^ should be used with caution. They might be relevant under Rawlsian fairness principles, which prioritise improving outcomes in the worst-off subgroup, but their implications require careful evaluation. Recall intergroup standard deviation,^92^ recall coefficient of variation,^64^ recall disparity ratio,^90^ and recall between-group generalised entropy index^93^ are highly complex as metrics, insufficiently studied, and difficult to interpret. Finally, Recall-HEAL^94^ introduces reparative fairness by computing an anticorrelation between model performance and historical disease burden. However, complex tradeoffs and ethical debates might arise, and the use of external data could be deemed arbitrary, making the use of this metric and related metrics questionable.

Among threshold-dependent performance-dependent metrics focusing on summary metrics, overall accuracy gap,^65^ error rate ratio,^95^ and balanced accuracy difference^75^) are inadvisable owing to improper metric behaviour (table 1), paucity of error-type distinction, and threshold dependency. F1 parity^58^ is inadvisable owing to improper scoring behaviour. The use of Matthew’s correlation coefficient parity^96^ is also questionable because of its highly complex formulation that hampers interpretability. The treatment equality^65^ metric has unclear rationale and implications.

Among metrics focusing on clinical utility metrics, only one metric—subgroup net benefit—reported in a preprint,^66^ was identified. Although further empirical evaluation is needed, this metric can be particularly relevant,^1,103,105^ as it reflects the quality of decision making (at a clinically meaningful threshold) and captures the tradeoff between potential harms and benefits. Net benefit is defined as sensitivity × prevalence – (1 – specificity) × (1 – prevalence) × *w*; here, *w* is the odds at the threshold probability. Net benefit is lower for subgroups with lower prevalence, reflecting expected differences.^103,105^ Subgroup net benefit, as defined by Benitez-Aurioles and colleagues,^66^ allows each subgroup to have its own outcome prevalence term, accounting for pre-existing prevalence differences or health inequities.

## Discussion

In this scoping review, we identified and examined 63 fairness metrics used in CPMs. We evaluated how each metric should be interpreted in light of relevant ethical and legal frameworks and assessed when the use of each of these metrics is justified. We carried out a qualitative critical appraisal and provided practical guidance that considers the implications, limitations, and appropriate contexts for each metric. The panel provides broader recommendations for the fairness evaluation of CPMs, emphasising that fairness evaluation is not restricted to the choice of fairness metrics and that transparent reporting is fundamental for fairness evaluation of CPMs.

Our scoping review revealed broader issues regarding fairness evaluation that warrant discussion. The definition of fairness metric in the literature is often vague or ill defined. This conceptual ambiguity undermined our ability to systematically assess the fairness of CPMs. To address this gap, we proposed a working definition of fairness metric, which also serves as the foundation for our methodology. Although diversity in methodological approaches can be beneficial, the high number of proposed metrics and definitions can result in redundancy and overlap, with subtle variations in their formulations leading to inconsistent interpretation of results.

Fairness metrics largely originate from computer science^39^ and are frequently evaluated in case studies unrelated to health care, such as recidivism prediction^99^ or credit scoring.^42^ These metrics often emerge in isolation or is at risk of epistemic trespassing^112^ in the absence of a clear ethical or theoretical foundation,^102^ raising concerns about the suitability of these metrics for clinical applications.^113^

Operational challenges increase as fairness metrics become more complex. Once fairness assessments move beyond binary classification tasks, binary sensitive attributes, or simple conditioning approaches, computing and interpreting these metrics becomes non-trivial.

We found that fairness metrics are often devoid of clear definitions, justified use, or sufficient empirical evaluation, restricting their reliability for real-world AI-based CPMs. Many metrics suffer from theoretical ambiguities, potentially inconsistent behaviour, or inadequate empirical support. The level of scrutiny applied for different metrics varies widely. Some metrics are rigorously studied and grounded in ethical or legal principles (eg, equality of opportunity difference^42^), whereas others are introduced with minimal justification (eg, equity-scaled AUROC^57^).

The predominance of performance-dependent (48 of 63) and threshold-dependent (33 of 63) metrics further underscores a methodological convenience bias. These metrics are easier to compute than performance-independent and probability-based metrics but might not capture a full and relevant picture of model behaviour. Performance-dependent metrics might preserve existing biases in the data rather than questioning them,^31^ whereas threshold-dependent metrics might be inherently inadequate as they often assess fairness at arbitrarily chosen decision cutoffs.^32^

Individual fairness metrics were notably scarce (two of 63), largely because individual metrics are difficult and sometimes controversial to operationalise. We recommend readers to refer to the study by Anderson and Visweswaran^52^ for more on individual fairness metrics. However, the authors^52^ found only six uses of individual fairness metrics in health care,^71–118^ and their operationalisation as metrics remains unclear.

Beyond group and individual fairness, intersectionality presents an important consideration in fairness assessment. Intersectionality captures how overlapping social identities interact to create unique experiences of privilege or oppression.^119^ Although metrics can be operationalised as group fairness metrics with varying subgroup granularity, intersectional or subgroup families of fairness metrics have been proposed.^68,120^ Existing fairness metrics often assess disparities along single attributes such as sex, age, or race, whereas real-world inequities arise at the intersections of these dimensions. For example, an 80-year-old Black woman might face disadvantages not captured by separate assessments of age, race, or sex.^121^

Another notable gap in the literature was the restricted presence of clinical utility metrics. Only one metric, subgroup net benefit,^66^ was explicitly defined as capturing clinical utility, despite the importance of such metrics for informing decision making.^1^ Two other metrics—equalising disincentives^84^ and treatment equality^65^—appeared to draw on utility reasoning; however, both of them were neither explicitly framed as clinical utility metrics nor originally proposed in a health-care context. Existing fairness metrics focus largely on parity in statistical attributes but do not expose any downstream effect on patient outcomes, which is arguably the most important aspect for models in a clinical setting. Implementing predictive AI models should ultimately translate into improved patient outcomes; therefore, future research should prioritise metrics that explicitly link fairness to clinical utility.^122^ For instance, subgroup-specific decision curves and net benefit-based fairness metrics could be of substantial value.^123^

Most fairness metrics are parity based and primarily capture numeric discrepancies between groups. However, these discrepancies do not necessarily correspond to clinically meaningful disparities. The interpretation of such discrepancies depends heavily on the performance regimen of the model. For instance, a difference of 0·10 between AUROC values of 0·90 and 0·80 across subgroups might have very different clinical implications than implications from the same difference between values of 0·70 and 0·60. This variability raises important considerations regarding when a disparity should prompt concern and whether statistical significance alone can be a basis for intervention. Rather than aiming for statistical parity at all costs, fairness should be framed in terms of minimum acceptable performance for all groups,^102^ guided by clinical relevance and known health disparities.

The downstream effects of fairness violations on CPM deployment is inherently context-dependent. For example, in scenarios in which all groups benefit from the model, the fact that one group benefits less than other groups might still be problematic. Although such a scenario could represent an improvement from previous standard practice, tolerating such disparities might have long-term implications. Once deployed, models are subject to data drift and performance degradation. Thus, groups that initially benefited less might be the first to experience a decline in performance, a phenomenon that has been described as fairness drift.^109^

The following crucial gaps were identified in fairness metric development and evaluation during our scoping review. Sample size requirements remain largely unaddressed during fairness evaluation.^124,125^ Fairness metrics rarely report confidence intervals to quantify statistical uncertainty; however, these intervals are fundamental for interpretation as groups typically have very different (and small) sample sizes.^126^ Next, intersectionality remains insufficiently addressed, as most fairness assessments treat sensitive attributes independently and overlook interactions across multiple axes of disparity.^40^ Intersectional analyses are methodologically challenging because of small subgroup sizes, even in large datasets.^124,127–129^ Clinical utility is rarely studied, despite its importance.^102^ Furthermore, the statistical behaviour of many fairness metrics are not empirically evaluated in medical settings, hampering their reliability and usefulness. Finally, tradeoffs between fairness metrics remain understudied for CPMs although some metrics are mutually incompatible.^82^

Future works should prioritise fairness metrics that provide uncertainty estimates (eg, by using bootstrap or closed forms when available), support intersectional analyses, align with clinical outcomes and benefit, and are systematically evaluated across real-world health-care settings.

We did not extensively assess the practical applications of fairness metrics in the literature. Future research should translate our qualitative critical appraisal into quantitative evidence via simulation studies or using real-world data and thereby support recommendations for selecting and applying fairness metrics in CPMs.

## Conclusion

The current landscape of fairness metrics in clinical predictive AI is fragmented, characterised by unclear definitions, standardisation, and clinical relevance. Several metrics are insufficiently evaluated empirically, hampering their real-world relevance. Future research should prioritise fairness evaluations that align with clinical decision making, incorporate uncertainty estimation, and account for intersectionality. Fairness evaluations should be conducted contextually and collaboratively with key actors.

## Supplementary Material

1

## Figures and Tables

**Figure:**
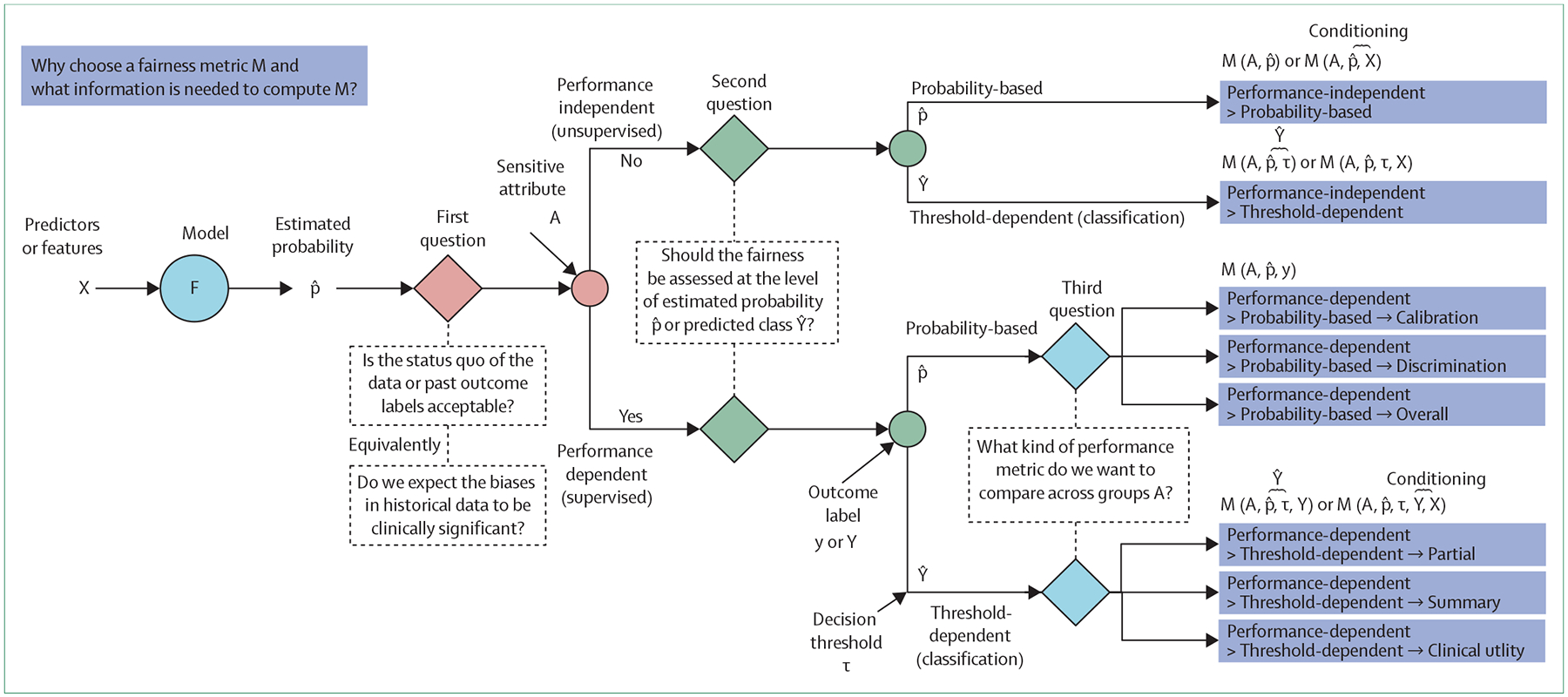
Decision diagram guiding the selection of fairness metrics for artificial intelligence-based clinical prediction models, structured according to taxonomy The figure provides a decision tree to guide the selection of fairness metrics, based on what information is available and what fairness objectives are prioritised. Input features X are processed by a model F, which outputs estimated probabilities $\hat{\text{p}}$. Using the sensitive attribute A, outcome labels y, decision thresholds *τ*, and predicted classes $\hat{\text{Y}}$, users navigate three questions, from left to right. First question (elements in red) is whether the status quo or data patterns are acceptable (ie, whether fairness is assessed independently of labels in an unsupervised manner or in relation to outcomes in a supervised manner). The second question (elements in green) is whether the focus of the metric is on the estimated probability (probability based) or on the predicted classification (threshold dependent). The final question (elements in blue) is regarding what kind of performance metric is the most adequate one to compare the fairness across individuals or groups. Each branch leads to a category of fairness metric, classified along two main axes: performance-independent versus performance-dependent; and probability-based versus threshold-dependent performance metrics. Subtypes of performance-dependent metrics include those that focus on calibration, discrimination, overall performance, partial metrics, summary metrics, and clinical utility. The necessary conditioning variables required to compute the fairness metric in each category are presented in the figure.

**Table 1:** Glossary of terms used in the scoping review

	Context	Definition
Algorithmic bias	Statistical	Condition in which data or analysis biases are encoded directly or indirectly into a model.
Bias	Societal	Condition in which an individual or group is consistently disadvantaged.
Bias (or systematic error)	Statistical	Consistent or proportional difference between the predicted and observed values.
Bias-preserving fairness metrics	Statistical	Fairness metrics that aim to maintain the patterns observed in the data used for model development, such as specialist follow-up referral rates. These metrics assume that the distributions and outcomes present in the data reflect acceptable baselines. Consequently, the metrics prioritise consistency in model performance across groups rather than adjusting for underlying biases to effectively preserve the status quo in health-care delivery.^31^
Bias-transforming fairness metrics	Statistical	Fairness metrics that do not assume that clinical or societal biases (eg, disparities in disease prevalence, access to care, or historical disease patterns) should be preserved during model development. Although the performance of these metrics is typically independent of model performance, the metrics aim to ensure parity in predicted positivity rates. Their goal is to actively modify the influence of underlying data biases to promote greater fairness in outcomes.^31^
Calibration	Statistical	The reliability of risk estimates, associated with the agreement between the estimated and observed proportion of events.^32^
Clinical utility	Statistical	Metrics and plots that evaluate the potential benefit of model-guided decisions by assessing whether such decisions might result in better outcomes or fewer harms than those observed for the results from alternative strategies (eg, standard care, treat-all, treat-none, or another model).
Clinical prediction model	Statistical	A model that aims to estimate the probability of present or future health outcomes, given a set of baseline predictors to facilitate medical decision making and improve people’s health outcomes.^33^
Decision threshold	Statistical	The probability cutoff at which a model categorises a sample as belonging to a particular outcome class, based on the estimated probability. In clinical settings, this threshold determines, for example, a planned intervention or diagnosis.
Discrimination	Societal	Prejudicial treatment against a group of individuals, based on group membership.
Discrimination	Statistical	How well the predictions from the model differentiate between individuals with and without the outcome.
Disparate impact	Societal	Condition in which a policy or practice has an adverse effect on a protected group (ie, a category of individuals who are legally safeguarded against discrimination under specific laws and regulations) despite appearing neutral.
Equality	Societal	Condition in which everyone should (in principle) get the same treatment, regardless of baseline conditions or potential outcomes.
Equity	Societal	Condition in which equals (ie, individuals with similar clinical presentation) are treated equally, and unequals (ie, individuals with distinct clinical presentation) are treated unequally; treat like cases alike such that everyone attains their full potential.
Evaluation (or performance) metric	Statistical	Quantitative measure or plot that is used to assess the performance or effectiveness of a prediction model’s output according to an outcome label (ie, ground truth or reference standard).
Fairness	Societal	Honesty, impartiality, equity, justice, and fair dealing.^34^
Fairness	Statistical	The property of a prediction model whereby individuals or groups defined by protected attributes (eg, age, race or ethnicity, sex or gender, or socioeconomic status) are not systematically disadvantaged in terms of model outputs or associated decisions.
Fairness metric	Statistical	A metric that quantifies the extent to which a model’s output does not discriminate (in the societal sense, based on a given notion of fairness) against individuals or groups defined by a sensitive attribute.
Fairness notion	Statistical	Definition of fairness that a model can satisfy or fails to satisfy. For example, counterfactual fairness is a notion according to which a decision is fair if it remains the same regardless of whether an individual belongs to a different group defined by a sensitive attribute.^35^
Group fairness	Statistical	Condition in which groups of individuals defined by sensitive attributes receive similar care.^36^
Hidden stratification	Statistical	Condition in which a model performs well at the population level but poorly in one or more subgroups, potentially leading to disparities in predictions.^37,38^
Individual fairness	Statistical	Condition in which similar individuals receive similar care.^39^ The concept of similarity is defined by a metric or distance that can be adjusted and agreed upon by relevant stakeholders (eg, patients, health-care professionals, and policy makers).
Intersectionality	Societal	The interconnected nature of social categorisations such as age, race or ethnicity, sex or gender, or socioeconomic status, which creates overlapping and interdependent systems of discrimination or disadvantage.^40^
Justice	Societal	Conformity (of an action or thing) to moral rights or to reason, truth, or fact.^41^
Performance-dependent metric	Statistical	A fairness metric that compares model performance across individuals or groups of individuals (eg, AUROC parity). See the definition of bias-preserving fairness metrics for implications.
Performance-independent metric	Statistical	A fairness metric that compares model behaviour (eg, by assessing parity in positivity rates) instead of comparing performance (eg, statistical parity) across individuals or groups of individuals. See bias-transforming fairness metrics entry for implications.
Probability-based metric	Statistical	A fairness metric in which estimated probabilities are used as input (eg, AUROC parity).^1^
Proper metric	Statistical	A performance metric is proper when its expected value is optimal only for the correct model.^1^
Semi-proper metric	Statistical	A performance metric is semi-proper when the expected value is optimal for the correct model and for some incorrect models.^1^
Sensitive (or protected) attribute	Statistical	A characteristic, feature, variable, or axis (such as age, race or ethnicity, sex or gender, or socioeconomic status) according to which fairness can be evaluated; often derived from legal frameworks.
Threshold-dependent metric	Statistical	A fairness metric in which predicted classes (a result of converting estimated probabilities using a decision threshold) are used as inputs (eg, equality of opportunity difference).^1^

AUROC=area under the receiver operating characteristic curve.

**Table 2:** Type of fairness metrics, their descriptions, and the number of metrics identified during the scoping review

Type of metric	Description or rationale	Individual fairness metrics	Group fairness metrics	Metrics proposed in health care
**Performance-independent or unsupervised metrics; model performance is not computed against an outcome label or ground truth**				
Probability-based metrics	Estimated probabilities are used (eg, mean score parity^59^)	2	6	3
Threshold-dependent metrics	Predicted classifications are used (eg, statistical parity^60^)	0	7	0
Total		2	13	3
**Performance-dependent or supervised metrics; model performance compared across individuals or groups of individuals**				
Probability-based metrics (estimated probabilities are used)				
Probability-based metrics focusing on discrimination	The model should assign higher probabilities to individuals who experience an event than to those who do not experience the same event (eg, AUROC parity^63^)	0	12	5
Probability-based metrics focusing on calibration	Probabilities estimated by the model should correspond to the observed event proportions (eg, equal calibration^82^)	0	5	1
Probability-based metrics focusing on overall performance	Probabilities estimated by the model, ranging from 0 to 1, should closely match actual outcomes (eg, Brier score parity difference^64^)	0	5	0
Subtotal		0	22	6
Threshold-dependent metrics (predicted classifications are used)				
Threshold-dependent metrics focusing on partial metrics	Individuals should be correctly classified according to their observed outcome, based on a partial view of the confusion matrix (eg, equal opportunity difference^42^)	0	18	6
Threshold-dependent metrics focusing on summary metrics	Individuals should be correctly classified according to their observed outcome, based on the whole confusion matrix (eg, overall accuracy gap^65^)	0	7	3
Threshold-dependent metrics focusing on clinical utility	Predicted classification should result in improved clinical decision making; subgroup net benefit^66^ was the only metric identified in this category	0	1	1
Subtotal		0	26	10
Total		0	48	16
**Grand total**		**2**	**61**	**19**

The data represent number of identified metrics. AUROC=area under receiver operating characteristics curve.

**Table 3:** Guidance and critical appraisal of fairness metrics according to their type in clinical prediction models based on qualitative assessment

	Guidance level (three levels in the order of most favourable to the least favourable: recommended if, use with caution, and inadvisable)	Comments
Probability-based performance-independent metrics		
Mean score parity^59^ and regression demographic parity (reported in a preprint)^67^	Use with caution, provided there is no difference in risk across subgroups	Can quickly summarise how a model behaves when comparing different subgroups; utility restricted as only a mean value is reported
Unsupervised ranking fairness,^69^ survival group fairness,^70^ censoring-based group fairness,^71^ survival intersectional fairness,^70^ survival individual fairness,^70^ and censoring-based individual fairness^71^	Inadvisable, unless the metrics are properly validated and implementation details are clarified	These metrics have not been sufficiently validated for use in health care
Threshold-dependent performance-independent metrics		
Statistical parity,^60^ children metrics,^68,72^ and disparate impact^73^	Use with caution, provided the positivity rate is relevant, there is no difference in risk across subgroups, and the chosen threshold is appropriate	Useful only if the positivity rate is relevant in the context of the application of the model; the relevance will be dependent on the selected threshold, and therefore, also on the context, ie, parity with one threshold value does not guarantee parity with other threshold values
Conditional statistical parity^48^	Recommended if there is no expected interaction between the legitimate factor and the protected attribute	Relevant in legal and clinical contexts in which consistent positivity rates are important despite the fact that the metric might be legitimately affected by other factors such as comorbidities; aligned with EU non-discrimination law in light of contextual equality^97^
Differential fairness^68^	Inadvisable as the metric is not validated in health-care contexts	No empirical evaluation or considerable validation in health care
Probability-based performance-dependent discrimination metric		
AUROC parity^63^	Recommended if paired with a calibration-related parity metric	Quantifies disparity in discrimination using AUROC; AUROC parity metric should be paired with calibration metrics as AUROC focuses only on discrimination
AUPRC parity^64^	Inadvisable, as the base metric AUPRC is not proper and its use is not advisable	AUPRC by itself is an inadvisable metric^1,98^ and can be an explicitly discriminatory metric that favours high-prevalence subgroups^98^
xAUROC,^76^ xAUROC disparity,^76^ sAUROC,^77^ pairwise ranking fairness,^69^ pairwise ranking fairness disparity,^69^ equity-scaled AUROC,^57^ mean- performance-scaled disparity AUROC,^74^ max-performance-scaled disparity AUROC,^74^ and concordance imparity^79^	Inadvisable owing to insufficient validation and implementation details; some metrics could become acceptable after further investigation	Insufficient knowledge or inadequate support to use these metrics; defined or proposed seldom in the health-care domain; some of these metrics could become relevant after further research and empirical evaluation—however, currently, using these metrics is inadvisable
Probability-based performance-dependent calibration metric		
Equal calibration^82^ and well calibration^51^	Use with caution, as implementation details are insufficient and the metrics should be paired with a calibration plot per group and a discrimination metric	Details on how to operationalise remain unclear, as the metric definition is not specific in terms of implementation; metrics that satisfy the notion of equal calibration across groups would (in principle) be desirable^99^
Expected calibration error parity,^78^ absolute calibration error parity,^81^ and calibration-in-the-large parity^62^	Use with caution, as the metrics should be paired with a calibration plot per group and a discrimination metric	Can be informative but a calibration plot should be presented for each group; should be paired with discrimination metrics. Expected calibration error and absolute calibration error have been criticised for being dependent on how binning is done and for handling overestimation and underestimation equally;^100^ the behaviour of fairness metrics focusing on the differences between the expected calibration error and absolute calibration error is not well studied
Probability-based performance-dependent overall metrics		
Log-loss parity^81^ and Brier score parity^64^	Use with caution but not essential	Log-loss and Brier score are proper metrics but hard to interpret and not very informative owing to their overall nature, mixing discrimination and calibration
Earth mover’s distance for equivalent separation,^64^ balance for positive class,^82^ and balance for negative class^82^	Use with caution but hard to interpret and should be validated further	Although the metrics are relevant in principle and some have been used in health care, they have not been empirically evaluated; the metrics, especially Earth mover’s distance, are hard to interpret
Threshold-dependent performance-independent partial metrics		
Equal opportunity difference,^43^ recall equality difference,^89^ and predictive equality^83^	Use with caution, provided they are reported together and if true-positive and true-negative rates are relevant	The choice of a decision threshold is often arbitrary; if the threshold is being appraised, reporting these metrics together can be descriptively informative—nevertheless, the base metrics are improper on their own^1,98^
Predictive parity^87^ and negative predictive value difference^75^	Use with caution if reported together and if positive predictive values and negative predictive values are relevant	The choice of a decision threshold is often arbitrary; if the threshold is being appraised, reporting these metrics together can be descriptively informative—nevertheless, the base metrics are improper on their own^1,98^
Equalised odds,^43^ equalising disincentives,^84^ average odds difference,^85^ average disparity in equalised odds,^86^ conditional equality of opportunity gap,^88^ and conditional use accuracy equality^64^	Inadvisable despite being widely used in the literature	Unclear how true-positive and true-negative rates or positive predictive values and negative predictive values should be combined, as the aggregation of both quantities are not always well defined; naming conventions can be misleading (eg, use of the term odds); these metrics are often incompatible with each other; for example, if prevalence differs across subgroups, equalised odds and predictive parity cannot be achieved simultaneously^101^
Worst-group recall,^91^ maximum difference recall^89^	Use with caution, descriptively, if Rawlsian fairness notions are sought and outliers are not expected	Linked to Rawls’ Theory of Justice (minimum benefit should be maximised across groups); can have unintended consequences on levelling down;^102^ these metrics are sensitive to outliers; for example, if a single group with poor performance, for example, because of small sample size, exists
Recall intergroup standard deviation,^92^ recall coefficient of variation,^64^ recall disparity ratio,^90^ and recall between-group generalised entropy index^93^	Inadvisable because of overcomplexity	Scenarios for justified use are unclear and expected behaviour has not been sufficiently explored; hard to interpret
Recall-HEAL^94^	Use with caution, as validation is required	Relates to reparations theory, which society might want to promote; complex tradeoffs and ethical debates might arise; the use of external data can be deemed arbitrary
Threshold-dependent performance-independent summary metrics		
Overall accuracy gap,^65^ error rate ratio,^95^ balanced accuracy difference,^75^ FI parity,^58^ Matthew’s correlation coefficient parity,^96^ and error distribution disparity index^86^	Inadvisable owing to improper behaviour and threshold dependency	Flawed models might yield higher values than robust ones; all errors are treated in the same way, and some metrics are hard to interpret; threshold dependency is restricted to one clinical decision threshold^1,98^
Treatment equality^65^	Inadvisable	Appears to be utility inspired but not explicitly defined as such, and the implications remain unclear
Threshold-dependent performance-independent clinical utility metrics		
Subgroup net benefit (as reported in a preprint^66^)	Recommended if a clear clinical utility rationale is present	The most relevant category of metric among all performance-independent metrics, as it combines discrimination and calibration;^103^ the formulation assumes different prevalence per subgroup;^66^ requires further empirical evaluation

AUROC=area under the receiver operating characteristic curve. AUPRC=area under the precision–recall curve. sAUROC=subgroup area under the receiver operating characteristic curve. xAUROC=cross-area under the receiver operating characteristic curve.

**Table 4:** Different metric categories and examples of CPMs showing how these categories can be used

	Clinical example
Performance-independent (Hypothetical scenario)	Context: In a 2019 study by Obermeyer and colleagues,^22^ a health-care model showed racial bias because of the use of health-care costs as a proxy label to estimate future health needs. Because access to health-care services had historically been lower among Black patients than among White patients, this label systematically underestimated the health burden in this population. Although the model showed high overall performance, the underlying bias went undetected until after deployment. Metric: Had a performance-independent metric been used during development, this disparity might have been identified earlier.
Threshold-dependent performance-independent (statistical parity^60^)	Context: Ravindranath and colleagues^104^ described various approaches for developing a CPM to estimate whether patients with glaucoma will require incisional glaucoma surgery within 12 months of assessment. Electronic health record data from nearly 40 000 patients across seven US health systems were used to develop the model. Metric: Demographic parity (termed independence) was used as the fairness metric to measure whether the model’s prediction rates were equal across different demographic groups regardless of actual outcomes. Models that excluded sensitive attributes were less fair than those that included sensitive attributes (eg, with respect to sex, a demographic parity of 0·134 was obtained for the model that excluded sensitive attribute *vs* that for the model that included sensitive attribute, ie, 0·038).^104^
Performance-dependent (hypothetical scenario)	Context: Consider a CPM used to guide prostate biopsy decisions. In this context, men with elevated PSA levels, typically 3 ng/mL or higher, might be referred for biopsy. However, only a small proportion of those referred for biopsy are ultimately diagnosed with high-grade cancer. Biopsy results, which serve as the ground-truth label in this context, are reliable in identifying the presence and aggressiveness of cancer. Prediction models have been developed to improve risk stratification, recommending biopsy when there is a high risk of clinically significant disease.^103,105^ Metric: This scenario can be viewed as a justified application of bias-preserving metrics, as the outcome is based on a well established and clinically meaningful reference standard. However, it is important to recognise that such assessments might overlook disparities in access to follow-up testing or subpopulation-level differences in PSA kinetics. Even in these circumstances, a careful use of stratification and performance-independent metrics, such as conditional statistical parity, can be a useful diagnostic aid. Differences between the positivity rates of subpopulations should be explained by differences in known risk factors between the groups; a scenario in which such an explanation is not possible can indicate the importance of a previously unconsidered risk factor or some form of sampling selection bias during the data acquisition process.
Probability-based performance-dependent discrimination metric (AUROC parity^63^)	Context: Byrd and colleagues^80^ evaluated the performance of Epic’s proprietary deterioration index, a prognostic CPM that estimates the risk of clinical deterioration in patients who are hospitalised. Deterioration was defined as mechanical ventilation, intensive care unit transfer, or death. Metric: AUROC parity was calculated as the ratio of the AUROC for a protected group to that of a reference group, with values closer to 1·0 indicating similar discriminative performance. The AUROC parity was higher than 1·00 for most groups except for those with unknown ethnicity,^80^ which had a score of 0·93.
Probability-based performance-dependent calibration metric (ACE parity^81^)	Context: Pfohl and colleagues^81^ described a CPM for atherosclerotic cardiovascular disease risk using electronic health record data from over 250 000 patients. The researchers utilised adversarial learning techniques to create a model that ensures similar error rates (ie, similar proportion of classification errors after setting a decision threshold) across different demographic groups defined by race, sex, and age. Metric: ACE parity was used to quantify differences in calibration between subgroups. ACE parity assesses whether models consistently overestimated or underestimated risk across groups.^81^ Calibration is particularly important in this setting, as clinical guidelines recommend initiating interventions based on fixed risk thresholds of 7·5%, which define an intermediate-risk category, or of 20%, which define high-risk category.^106^
Probability-based performance-dependent overall metric (EMD for equivalent separation^64^)	Context: Pfohl and colleagues^81^ described a CPM for atherosclerotic cardiovascular disease risk using electronic health record data from over 250 000 patients. The researchers utilised adversarial learning techniques to create a model that ensures similar error rates (ie, similar proportion of classification errors after setting a decision threshold) across different demographic groups defined by race, sex, and age. Metric: Pfohl and colleagues^64^ used EMD (for equivalent separation), which quantifies differences between the distributions of estimated atherosclerotic cardiovascular disease risk across subgroups of patients (using the same model as that used in another study by Pfohl and colleagues^81^), conditioned on the true outcome. Their results showed that the adversarial training approach resulted in a substantial reduction in the mean pairwise EMD between each predictive distribution in both outcome strata for both sex and age, with a negligible effect for race. For example, for the positive outcome, with respect to sex, a standard modelling approach achieved an EMD of 0·0167, while the improved model achieved an EMD of 0·00593, indicating an improvement of fairness, according to this metric.^64^
Threshold-dependent performance-independent partial metric (predictive equality^83^)	Context: Yang and colleagues^107^ developed several CPMs for the prognosis of three key outcomes in patients in the intensive care unit: in-hospital mortality, 30-day re-admission, and 1-year mortality. The researchers linked the publicly available MIMIC-IV electronic health record database^108^ with community-level social determinants of health data to assess whether the inclusion of social determinants of health features would enhance predictive performance. Metric: To evaluate algorithmic bias across demographic groups, the authors utilised predictive equality (false-positive rate parity). For demonstration purposes, a decision threshold of 0·5 was used to convert estimated probabilities into binary classifications. False-positive rate parity indicates whether the model disproportionately flags some groups as high-risk groups. The analysis revealed that patients who were older, Black, women, from communities with lower incomes, or more frequent users of public transit, or those with lower educational attainment showed higher false-positive rates, and thus, lower predictive equality.^107^
Threshold-dependent performance-independent summary metric (overall accuracy gap^65^)	Context: Davis and colleagues^109^ described the development of random forest models predicting three post-surgical outcomes—namely, 30-day mortality, unplanned re-admission, and pneumonia—among veterans receiving surgical care at the US Department of Veterans Affairs facilities. The models were built using features based on the American College of Surgeons NSQIP universal risk calculator^110^ using data from 2013 and evaluated for performance drift over a 10-year period. Metric: Classification thresholds for each model were based on the estimated probability that maximised the mean of the specificity and sensitivity in the entire population. This accuracy gap metric quantified the difference in classification accuracy between disadvantaged versus advantaged groups (Black *vs* White patients and female *vs* male patients). The study found that, for instance, the pneumonia model showed increasing accuracy gaps between Black and White patients over time, with larger performance declines being observed for Black patients.^109^
Threshold-dependent performance-independent clinical utility metric (subgroup net benefit^66^)	Context: Benitez-Aurioles and colleagues^66^ developed three logistic regression models predicting the 5-year incidence of type 2 diabetes to assist clinicians in referring patients to lifestyle intervention programmes. The models were built on a UK Biobank dataset of 477 558 patients using relevant demographic and clinical predictors. A model excluding ethnicity as a predictor (LogNoSA), a model including ethnicity as a predictor (LogSingleSA), and an ensemble of models trained separately for each ethnic group with propensity score weighting (LogMultiSA) were developed. The researchers set a clinical threshold of 15% risk (based on the Leicester Diabetes Risk Score used in clinical practice^111^) and a treatment weight of 0·58 (corresponding to the reported relative risk reduction of a diabetes prevention programme). Metric: Fairness was assessed by comparing subgroup net benefit across different ethnic groups. The subgroup net benefit quantifies the clinical utility of a model in terms of true negatives, per 10 000 patients. The analysis revealed that the models including ethnicity as a predictor (LogSingleSA) were associated with an improved net benefit for Asian (n=9435) and Black (n=9597) populations compared with those observed for models excluding ethnicity.^66^

ACE=absolute calibration error. AUROC=area under the receiver operating characteristic curve. CPM=clinical prediction model. EMD=Earth mover’s distance. PSA=prostate-specific antigen.

## Panel: Key considerations and recommendations for fairness evaluation in clinical prediction models

- Evaluating model performance at the population level will mask unfairness; defining subgroups for evaluation by clinically relevant sensitive attributes is therefore a fundamental first step to any fairness assessment
- Although fairness metrics can be valuable for summarising potential biases in model outputs, fairness evaluation is not restricted to our definition of fairness metric
  - Although not necessarily labelled as a fairness metric, subgroup performance evaluation remains essential for identifying groups in which the model is underperforming
  - Metrics related to discrimination, calibration, and clinical utility should be prioritised; fairness evaluations should be grounded to improve patient outcomes, which can be measured using clinical utility metrics, which are arguably the most important for models in a clinical setting
  - Visual tools such as calibration plots and decision curve analysis, when stratified by sensitive attributes, are fundamental to highlighting performance differences and guiding fairness evaluations
  - For cases in which the validity of outcome labels is uncertain, performance-independent fairness metrics can help to challenge and interrogate the status quo embedded in historical data used for model development
- Performance and model behaviour will vary across subgroups; rather than aiming for statistical parity at all costs, fairness should be framed in terms of minimum acceptable performance for all groups, guided by clinical relevance and known health disparities
- When clinically defined thresholds exist (eg, from clinical guidelines), threshold-dependent metrics such as sensitivity might be descriptively informative when correctly paired (eg, sensitivity and specificity together or positive predictive value and negative predictive value together); however, the interpretation of such threshold-dependent metrics should be constrained to that specific threshold as extrapolating beyond this threshold might mislead the conclusions. Clinical utility metrics are the only threshold-dependent metrics that are advised on their own.
- Transparent reporting is essential, especially given the complexity and plurality of fairness definitions. In addition to adhering to TRIPOD+AI guidelines, we recommend explicitly defining and justifying all fairness-related design decisions, metrics, and visualisations.
- Fairness should be defined through interdisciplinary dialogue. Proposed metrics should involve diverse actors (eg, patients, clinicians, caregivers, and policy makers), whose backgrounds reflect the affected populations. A small, unrepresentative group unilaterally defining fairness for all groups is unacceptable.
