# Supplementary material for: Critical appraisal of fairness metrics for artificial intelligence-based clinical prediction models: a scoping review
Source: Lancet Digit Health. Author manuscript; Available in PMC 2026 Aug 17. (PMC13479377; doi:10.1016/j.landig.2026.101001)
Supplement: Supplementary file 1 [file NIHMS2201143-supplement-1.pdf]

THE LANCET
Digital Health

Supplementary appendix

This appendix formed part of the original submission and has been peer reviewed.
We post it as supplied by the authors.

Supplement to: Matos J, Van Calster B, Celi LA, et al. Critical appraisal of fairness metrics for artificial intelligence-based clinical prediction models: a scoping review. *Lancet Digit Health* 2026. https://doi.org/10.1016/j.landig.2026.101001

Critical Appraisal of Fairness Metrics in Clinical Predictive AI

Supplementary Material 1: Data extraction forms

João Matos, MSc [1], Ben Van Calster, PhD [2,3,*], Leo Anthony Celi, MD [4,5,6], Paula Dhiman, PhD [1], Judy Wawira Gichoya, MD [7], Richard D. Riley, PhD [8,9,*], Chris Russell, PhD [10], Sara Khalid, PhD [1], Gary S. Collins, PhD [1,*]

[1] Centre for Statistics in Medicine, Nuffield Department of Orthopaedics, Rheumatology and Musculoskeletal Sciences, University of Oxford, Oxford, UK
[2] Department of Development and Regeneration, KU Leuven, Leuven, Belgium
[3] Department of Biomedical Data Science, Leiden University Medical Centre, Leiden, Netherlands
[4] Beth Israel Deaconess Medical Center, Boston, MA, USA
[5] Laboratory for Computational Physiology, Massachusetts Institute of Technology, Cambridge, MA, USA
[6] Department of Biostatistics, Harvard T H Chan School of Public Health, Boston, MA, USA
[7] Department of Radiology and Imaging Sciences, Emory University, Atlanta, GA, US
[8] Department of Applied Health Sciences, School of Health Sciences, College of Medicine and Health, University of Birmingham, Birmingham, UK
[9] National Institute for Health and Care Research (NIHR) Birmingham Biomedical Research Centre, Birmingham, UK
[10] Oxford Internet Institute, University of Oxford, Oxford, UK

[*] Full Professor

Data Extraction Forms............ 2
1. Article-level data extraction............ 2
2. Catalogue of Fairness Metrics............ 2
3. Fairness Metrics, main properties, and classification according to different taxonomies.. 2
4. Operationalisation of Fairness Metrics............ 3
5. Interpretation of Fairness Metrics............ 3
6. Critical Appraisal of Fairness Metrics............ 4

Data Extraction Forms

1. Article-level data extraction

The first data extraction step was to analyse the papers that were deemed relevant for our review. For each paper that was included (because of a relevant fairness metric being used, defined, or proposed in the context of a clinical prediction model), we extracted the following article metadata:

- **Reference**
- **Article Title**
- **Venue:** journal or conference where the article was published
- **Article Identification Method:** Database Querying, Backward Citation Tracking, or Grey Literature
- **Name of the Fairness Metric(s)**
- **Community:** AI, (Applied Ethics), or Biomedical
- **Year:** publication year
- **Number Citations:** on Google Scholar, as of 3rd Apr 2025

2. Catalogue of Fairness Metrics

Next, for each fairness metric, basic information, implementation details, and considerations were extracted from the original paper and other related papers:

- **Overall description:** summarisation of metric
- **Mathematical Definition:** formulation
- **Interpretation**
- **Implications**

3. Fairness Metrics, main properties, and classification according to different taxonomies

The following were extracted:

- **Metric name:** common name with which the metric is referred to, although other aliases may exist (see the next step);
- **Reference:** the original paper where the metric was *proposed* for the first time; *defined*, for example in a significant review; or *used* in a publication involving evaluation of clinical prediction models;
- **Defined for healthcare:** whether the metric was originally proposed in the healthcare domain and, if so, in which kind of modality;
- **Performance dependency:** whether the metric is dependent on performance (i.e, compares a pre-existing performance metric) or independent from performance (i.e, assesses the model output independently)
- **Model output type:** probability-based or threshold-independent

- **Performance metric domain:** taxonomy applied to the base performance metric, if applicable. Based on (1)
- **Fairness type:** individual or group fairness
- **Group fairness axiomatic criteria:** also known as statistical non-discrimination; three criteria that aim to define the absence of discrimination in terms of statistical expressions and that be associated with certain classification fairness metrics. Such criteria can be fulfilled "equivalently" or in a "relaxed" manner (127) .

4. Operationalisation of Fairness Metrics

Here we focus on how to operationalise / compute each of the metrics that were found. The following are extracted:

- **Metric name**: common name with which the metric is referred to, although other aliases may exist;
- **Aliases**: alternative names for the same metric;
- **Parent fairness metric**: original fairness metric from which the present metric was (potentially) derived;
- **Base prediction type / performance metric:** type of model prediction that the metric takes as an input (either an estimated probability or threshold-dependent predicted class) or base performance metric that is computed across groups and then compared for the sake of fairness (any ML performance metric is eligible);
- **Use of ground-truth:** whether the metric considers the actual ground-truth (or label) present in the dataset. This will have implications on whether the metric is bias-preserving (i.e., accepts the *status quo*) or bias-transforming (i.e., aim for substantive equality and challenge existing historical labels that may be biased) (53)
- **Conditioning:** whether the metric considers the features used in the model training to create strata and compare groups of patients no only according to a sensitive attribute but also according to such features;
- **Sensitive attribute:** type of sensitive attribute that the metric supports. Can be binary, multi-group, or continuous.

5. Interpretation of Fairness Metrics

Here we analyse each of the metrics from an interpretation and implications perspective. The following are extracted:

- **Description:** short summarisation of the metric
- **Bias-preserving vs bias-transforming**: whether the metric is bias-preserving (i.e accepts the status quo) or bias-transforming (i.e aim for substantive equality and challenge existing historical labels that may be biased);
  Prevalence-dependency: whether the base prevalence per sensitive attribute group
- **Target value**: value that represents "perfect fairness";

- **Direction to be fairer**: direction that the metric takes when fairness is improved, towards a target value;
- **Potential legal or ethical connection**: key concepts or notions of fairness in the literature or in society that can be linked to each metric. Sparsely populated because not all metrics are morally or legally grounded.

6. Critical Appraisal of Fairness Metrics

When relevant, critical appraisal of each identified metric is performed according to the following angles:

- **Justified use:** scenarios in which assessing a model according to that metric is advisable;
- **Example in a clinical prediction model:** clinical prediction model in which the use of the metric fits its justified use;
- **Direct harm:** potential harm to the patient when a metric is not satisfied or is not as good as it should be;
- **Comment:** open questions and further comments.

Supplementary Material 2: Scoping review extra materials

Table of Contents

Supplementary Figure 2.1. PRISMA diagram for Scoping Reviews....6
Supplementary Table 2.1. Search queries per data source.... 7
Supplementary Table 2.2. Definitions of “fairness metric” and similar key concepts in literature.... 8
Supplementary Table 2.3. Fairness Metrics Taxonomies in the literature (in authors’ words). 9
Supplementary Table 2.4. Non-exhaustive review of existing reviews that cover existing fairness metrics.... 13
Supplementary Table 2.5. Individual sources of data (on an article basis) and extracted metrics per source.... 15
Supplemental Table 2.6. Summary statistics of communities, venues, and article identification method for each included article.... 24

Supplementary Figure 2.1. PRISMA diagram for Scoping Reviews

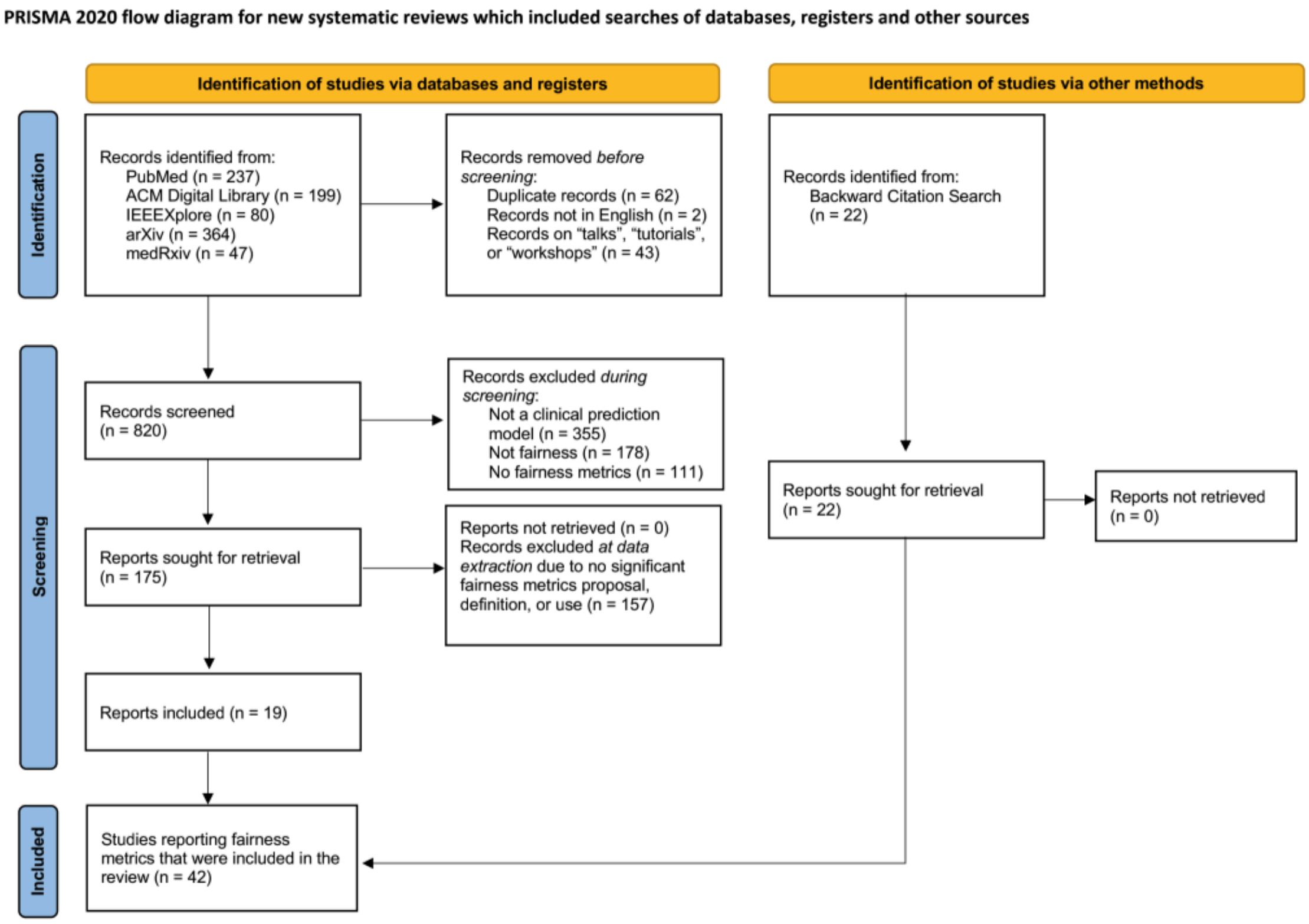

Source: Page MJ, et al. BMJ 2021;372:n71. doi: 10.1136/bmj.n71.

Supplementary Table 2.1. Search queries per data source

Database	Search String	# Results
PubMed	fair* AND (model* OR machine learning OR algorithm*) AND (health* OR clinic* OR medic*) AND metric*	237
IEEEXplore	fair* AND (health* OR clinic* OR medic*) AND (model* OR algorithm* OR machine learning)	561
ACM	fair* AND (model* OR algorithm* OR machine learning) AND (health* OR clinic* OR medic*)	199
arXiv	fair* AND model* AND health*	364
medRxiv	fair* AND model* AND health*	47

Differences in search terms across databases reflected both platform-specific technical limitations and the language conventions of their target communities. In PubMed, including the term “metric” helped focus the search on quantitative methods, whereas in ACM, IEEE Xplore, it was overly broad and often referred to geometric or algorithmic measures, increasing noise. Additionally, arXiv and medRxiv offered only basic keyword searches with limited logical operators, restricting specificity and requiring tailored terms to optimise yield and relevance.

Supplementary Table 2.2. Definitions of "fairness metric" and similar key concepts in literature

Citation	Concept	Definition
(46)	Fairness Metric	*A mathematical definition of fairness that is measurable.*
(128)	Fairness Metric	*Fairness metrics are important tools for evaluating potential disparities across sensitive features in the field of prediction modeling.*
(50)	Fairness Notion	*Definitions of fairness that a given model can either satisfy or fail to satisfy*
	Fairness Metric	*Mathematical formulae for measuring how close a given model is to satisfying some fairness notion (for example, "Equal Opportunity" is a fairness notion, and "Equal Opportunity Difference" is a fairness metric that measures it).*
(129)	Fairness Metric and Notion	*Different fairness metrics formalize varying intuitive notions of fairness.*
(130)	Fairness Metric	*A quantification of unwanted bias in training data or models.*
	Bias mitigation algorithm	*A procedure for reducing unwanted bias in training data or models.*
(131)	Fairness Metric	*Many quantitative metrics have been developed to assess fairness in AI, mostly from the perspective of "equality": a fair model should have equal performance in subgroups with respect to sensitive variables. Two types of fairness metrics are discussed most often: group-based and individual-based*

Note: This table highlights how seldom the concept of "Fairness Metric" is defined in the literature we reviewed. Moreover, the definitions are not necessarily in agreement.

Supplementary Table 2.3. Fairness Metrics Taxonomies in the literature (in authors' words)

**Citation**	**Level 1**	**Level 2**	**Prototype "Metrics"**<br>**(or "Notions", rather, according to our definition)**
(131)	Group Fairness	Parity-based	Demographic Parity
		Performance-based	Equal opportunity
		Rank-based	xAUROC Disparity
	Individual Fairness	Similarity-based	Fairness with awareness
		Counterfactual-based	Counterfactual fairness
	Distributive Fairness	Variance-based	Variance or std of quantities (e.g accuracy)
		Reward-based	Reward based on correlation
(127)	Independence	Equivalent	Demographic parity
		Relaxed	Conditional statistical parity
	Separation	Equivalent	Equalised odds
		Relaxed	Equal opportunity
	Sufficiency	Equivalent	Conditional use accuracy
		Relaxed	Predictive parity
(53)	Bias preserving	-	Predictive parity
	Bias transforming	-	Demographic parity
(41)	Limit the effect of	–	Demographic parity

	decisions on disparities		
	Limit the effect of attribute on decisions	–	Fairness through unawareness; Counterfactual fairness
(132)	Group Fairness	–	Demographic parity
		–	Conditional demographic parity
		–	Error parity (Equal accuracy, equality of odds; predictive parity)
	Individual Fairness	–	Fairness through unawareness / blindness
		–	Fairness through awareness
	Causality-based Fairness	–	Counterfactual fairness
		–	Path-specific counterfactual fairness
(45)	Group-based Fairness	Parity-based Metrics (Independence)	Demographic Parity
		Confusion Matrix-based Metrics (Separation)	Equalised Odds
		Calibration-based Metrics (Sufficiency)	Well calibration
		Score-based Metrics	Balance for positive class
	Individual and Counterfactual Fairness	Distribution-based Metrics	Counterfactual Fairness, Generalised Entropy Index
(46)	Individual Fairness (* the authors exclusively focus on Individual Fairness)	Generally defined distance metric	–
		Domain-specific distance metric	–
		Learned distance metric	–

		Distance in counterfactuals	–
(40)	Statistical measures	Definitions Based on Predicted Outcome	Statistical parity
		Definitions Based on Predicted and Actual Outcomes	Predictive parity
		Definitions Based on Predicted Probabilities and Actual Outcome	Test-fairness
	Similarity-based measures	–	Causal discrimination; fairness through unawareness; fairness through awareness
	Causal reasoning	–	Counterfactual fairness; no unresolved discrimination; no proxy discrimination; fair inference
(47)	Group Fairness	–	Equal false discovery rate (FDR)
	Individual Fairness	–	–
	Equality of Opportunity	–	–
	Demographic Parity	–	–
	Equalised Odds	–	–
	Counterfactual Fairness	–	–
(133)	Group Fairness Measurements	–	Demographic parity
	Individual Fairness Measurements	–	Counterfactual fairness
	Hybrid Measurements	–	Differential fairness
(134)	Fairness definitions	Unconstrained utility maximisation and	Single-threshold rule

	from data alone	single-threshold fairness	
		Equal prediction measures<br>• Definitions from confusion matrix<br>• Analogues with scores	Equal accuracy<br>• Equality of FPR<br>• AUC Parity
		Equal decision measures	Demographic parity
	Fairness definition incorporating additional context	Metric Fairness	Individual fairness (based on a metric which guarantees that closeness implies closeness)
		Causal definitions	Counterfactual Fairness
(54)	Distributive justice principle prioritised	Equal benefit	Clinical utility in terms of net benefit
		Equal performance	Equalised odds
		Equal allocation	Statistical parity
	Decision type influenced	Non-polar (patient benefits from high-performing model)	–
		Polar (patient benefits from biased high or low prediction)	–
	Intended use	Informing tools (risk scoring 0-100%)	Well calibration
		Decision support tools (classification [0, 1])	Equalised odds

Note: This table highlights how many different taxonomies have been proposed, how incompatible or in disagreement they are, and how certain fairness notions are often categorised as fairness metrics.

Supplementary Table 2.4. Non-exhaustive review of existing reviews that cover existing fairness metrics

Citation	Type of Review	Scope
(42)	Narrative (?)	“This article presents an overview of the main concepts of identifying, measuring, and improving algorithmic fairness when using ML algorithms, focusing primarily on classification tasks.”
(43)	Survey	“In this survey, we investigated different real-world applications that have shown biases in various ways, and we listed different sources of biases that can affect AI applications. We then created a taxonomy for fairness definitions that machine learning researchers have defined to avoid the existing bias in AI systems. In addition to that, we examined different domains and subdomains in AI showing what researchers have observed with regard to unfair outcomes in the state-of-the-art methods and ways they have tried to address them.
(41)	Unclear	“Here we first assemble and categorize these definitions into two broad families: (1) those that constrain the effects of decisions on disparities; and (2) those that constrain the effects of legally protected characteristics, like race and gender, on decisions. (...) We conclude by listing several open challenges in fair machine learning and offering strategies to ensure algorithms are better aligned with policy goals.”
(45)	Survey	“This article seeks to provide an overview of the different schools of thought and approaches that aim to increase the fairness of Machine Learning. It organizes approaches into the widely accepted framework of pre-processing, in-processing, and post-processing methods, subcategorizing into a further 11 method areas”
(135)	Scoping	“Thus, the objectives of this scoping review were to summarize existing literature and identify gaps in the topic of tackling algorithmic bias and optimizing fairness in AI/ML models using real-world data (RWD) in health care domains.”
(132)	Unclear	“A plethora of different definitions of fairness in ML have been proposed, that consider different notions of what is a “fair decision” in situations impacting individuals in the population. The precise differences, implications and “orthogonality” between these notions have not yet been fully analyzed in the literature. In this work, we try to make some order out of this zoo of definitions.”
(136)	Narrative (?)	“First, we explicate the various choices and assumptions made—often implicitly—to justify the use of prediction-based decision-making. Next, we show how such choices and assumptions can raise fairness concerns and we present a notationally consistent catalog of fairness definitions from the literature. In doing so, we offer a concise reference for thinking through the choices, assumptions, and fairness considerations of prediction-based decision-making”
(44)	Textbook	Fairness and Machine Learning: Limitations and Opportunities
(40)	Unclear	“this paper collects the most prominent definitions of fairness for the

		algorithmic classification problem, explains the rationale behind these definitions, and demonstrates each of them on a single unifying case-study”
(137)	Survey	“This paper is a survey of fairness notions that, unlike other surveys in the literature, addresses the question of "which notion of fairness is most suited to a given real-world scenario and why?". Our attempt to answer this question consists in (1) identifying the set of fairness-related characteristics of the real-world scenario at hand, (2) analyzing the behavior of each fairness notion, and then (3) fitting these two elements to recommend the most suitable fairness notion in every specific setup. The results are summarized in a decision diagram that can be used by practitioners and policy makers to navigate the relatively large catalogue of ML fairness notions.?
(47)	Narrative	“This paper provides a concise survey of fairness metrics applied in healthcare AI, including their mathematical representations, suitable use cases, and limitations, which are lacking in the existing literature.”
(46)	Scoping	“We conducted a scoping review on algorithmic individual fairness (IF) to understand the current state of research in the metrics and methods developed to achieve IF and their applications in healthcare.”
(133)	Survey	“In this survey, we review the current progress of in-processing fairness mitigation techniques. Based on where the fairness is achieved in the model, we categorize them into explicit and implicit methods, where the former directly incorporates fairness metrics in training objectives, and the latter focuses on refining latent representation learning.”
(138)	Systematic	“(...) review fairness literature in the context of machine learning and link it to core regulatory instruments to assist policymakers, AI developers, researchers, and other stakeholders in appropriately addressing fairness concerns and complying with relevant regulatory requirements.”

Previous efforts have reviewed fairness in machine learning but they rarely focus on clinical prediction models and do not provide comprehensive or critical perspective

Supplementary Table 2.5. Individual sources of data (on an article basis) and extracted metrics per source

Ref	Article Title	Description and Critical Appraisal	Venue	Article Identification Method	Fairness Metric(s)	Community	Year	Google Scholar Citations (3rd Apr 2025)
(63)	Fair transfer learning with missing protected attributes	Original research paper, with clear and formal definition of fairness metric, citing a previous study. Implications of the metric are not thoroughly studied. Focus is on missing sensitive attributes in the context of healthcare insurance.	ACM AIES	Database Querying	Mean Score Parity	(Applied) Ethics	2019	128
(56)	Bipartite ranking fairness through a model agnostic ordering adjustment	Original research proposing a fair ranking algorithm. Metrics are proposed to assess such an approach, without further validation of the metrics themselves. “Adult”, “MIMIC”, “eICU”, and “Framingham” datasets are used.	IEEEXplore	Database Querying	Unsupervised Ranking Fairness (URF), Pairwise Ranking Fairness, PRF Disparity (ΔPRF)	AI	2023	4
(57)	Fairness-enhancing mixed effects deep learning improves fairness on in- and out-of-distribution clustered (non-iid) data	Original research proposing a fair framework using mixed-effects deep learning. The fairness metric is proposed but its behaviour is not studied. “Heritage Health” dataset.	arXiv	Database Querying	Regression Demographic Parity	AI	2023	0
(58)	Equitable allocation of healthcare resources with fair cox models	Original research on fairness of survival models. Existing fairness metrics are extended here. While “fair learning” is assessed according to such metrics, these were not validated. “FLC Data” dataset.	arXiv	Database Querying	Survival Group Fairness, Survival Intersectional Fairness, Survival Individual Fairness	AI	2020	12
(59)	Fair and interpretable models for survival analysis	Original research. Authors propose fairness metrics based on survival functions and censoring. Fair survival models are proposed and assessed using such metrics, but metrics were not thoroughly validated and behaviour is unknown. “SEER dataset” which includes breast cancer patients and survival data.	ACM SIGKDD	Backward Citation Tracking	Censoring-Based Group Fairness, Censoring-Based Individual Fairness	AI	2022	24

(60)	Learning fair representations	Original research. Authors propose a fair model to achieve "group fairness". Fairness is assessed according to statistical parity, which is defined here, but not validated in detail. "Heritage Health" dataset is used to predict patient length of stay in the hospital.	ICML	Backward Citation Tracking	Statistical Parity	AI	2013	2,360
(61)	Generalized demographic parity for group fairness	Original research proposing the metric to address the fact that metrics largely overlook continuous sensitive attributes. Mathematical and empirical validation of metric behaviour is conducted. Clinical validation lacking.	ICLR	Backward Citation Tracking	Multi-group Demographic Parity, Generalised Demographic Parity (GDP)	AI	2022	80
(55)	An intersectional definition of fairness	Original research proposing and discussing metrics that could be informed by intersectionality. The added value of the metrics themselves, compared to existing ones, is questionable. Clinical validation lacking.	IEEEXplore	Backward Citation Tracking	Statistical Parity Subgroup Fairness, Differential Fairness	AI	2020	270
(41)	The measure and mismeasure of fairness	Review of several fairness metrics and definitions. Comprehensive and detailed. Defines the metric and explores some properties mathematically and in context, but lacks clinical validation.	arXiv	Backward Citation Tracking	Conditional Statistical Parity	(Applied) Ethics	2023	1,282
(62)	Certifying and removing disparate impact	Original research proposing a metric to measure disparate impact, as defined legally. This metric has been criticised elsewhere in the literature. This initial metric proposal lacks clinical validation.	ACM SIGKDD	Backward Citation Tracking	Disparate Impact	AI	2015	2,479
(71)	Longitudinal fairness with censorship	Original research proposing a metric and modelling approaches in longitudinal right-censored scenarios. Metric implementation is quite complex. Connection with sAUC is mentioned but further metric validation would be beneficial. "SUPPORT dataset", which is related to survival of inpatients.	ACM AAAI	Backward Citation Tracking	Concordance Imparity	(Applied) Ethics	2022	64
(123)	Validation of a Proprietary Deterioration Index Model and Performance in Hospitalized Adults	Original research evaluating "Deterioration Index" performance across different hospital settings and subgroups of patients. Metric is introduced without a reference or mathematical formulation, and	JAMA Network Open	Backward Citation Tracking	AUROC parity ratio	Biomedical	2023	20

		motivation is not clear. Data from 8 heterogenous Midwestern US hospitals (EHR).						
(64)	Can you fake it until you make it? impacts of differentially private synthetic data on downstream classification fairness	Original research focussed on privacy and fairness of models that are used to generate images (GANs) and then used in downstream prediction. Authors use AUC difference without references, formal definition, or investigation of its potential properties. “MIMIC-CXR”, “CheXpert”, and “ISIC archive” datasets used.	ACM FaaCT	Database Querying	AUROC Parity	(Applied) Ethics	2021	65
(65)	Creating fair models of atherosclerotic cardiovascular disease risk	Original research proposing the use of adversarial learning to develop fair models. Several metrics are used in evaluating the fairness of such models. These are used with minimal justification or validation. Data from “Stanford Medicine Research Data Repository”.	ACM AAAI	Database Querying	AUPRC Parity, Brier Score Parity, Earth Mover's Distance (EMD) for equivalent separation, Recall Coefficient of Variation	(Applied) Ethics	2019	84
(66)	The fairness of risk scores beyond classification: Bipartite ranking and the xAUC metric	Original research proposing relevant metrics. Mathematical definitions and properties are thoroughly studied. “Framingham” dataset was used for clinical validation.	NeurIPS	Backward Citation Tracking	Cross-Area Under the ROC Curve (xAUROC), xAUROC Disparity	AI	2019	87
(67)	(Predictable) performance bias in unsupervised anomaly detection	Original research on medical imaging fair modelling. Fairness metric introduced with minimal motivation and lacking validation. Data from “MIMIC-CXR”, “CXR14”, and “CheXpert”.	eBioMedicine	Database Querying	Subgroup AUROC (sAUROC)	Biomedical	2024	1
(51)	Harvard glaucoma fairness: a retinal nerve disease dataset for fairness learning and fair identity normalization	Original research on fair glaucoma prediction. Fairness and equity are used interchangeably. Fairness metric introduced with minimal motivation and lacking validation. “Harvard-GF” dataset.	IEEEXplore	Database Querying	Equity-Scaled AUROC	Biomedical	2024	19
(68)	A large-scale 3d imaging dataset for equitable eye diseases screening and fair identity scaling	Original research on eye disease prediction from images. Performance-scaled disparity measures are proposed with minimal justification or validation. “Harvard-EF” dataset was introduced and used.	arXiv	Database Querying	Mean Performance-Scaled Disparity (PSD), Maximum Performance-Scale	AI	2023	5

					d Disparity (PSD)			
(69)	Measuring and Reducing Racial Bias in a Pediatric Urinary Tract Infection Model	Original research evaluating an existing prediction model, UTICalc, used in young children. ECE parity is used without citation or validation. “UTICalc dataset” was used	AMIA	Database Querying	Expected Calibration Error (ECE) Parity	Biomedical	2024	3
(70)	Peeking into a black box, the fairness and generalizability of a MIMIC-III benchmarking model	Original research assessing the fairness of mortality risk prediction. Calibration-in-the-large is compared across subgroups as “is not supposed to be substantially different across demographic groups”, from which we derived the parity measure. Implications of computing this metric across subgroups, as opposed to overall population, are not studied.. “MIMIC” and “STARR” databases were used.	Scientific Data	Backward Citation Tracking	Calibration-in-the-large Parity	Biomedical	2022	70
(72)	Net benefit, calibration, threshold selection, and training objectives for algorithmic fairness in healthcare.	Original research that develops and evaluates models to estimate the ten-year risk of atherosclerotic cardiovascular disease to inform statin initiation. Evaluation is thorough and multiple metrics are used, though these are sometimes a different representation of similar performance domains, which make it hard to interpret. “Optum CDM” data was used.	ACM FaaCT	Database Querying	Absolute Calibration Error Parity, Log-Loss Parity	(Applied) Ethics	2022	36
(73)	Inherent trade-offs in the fair determination of risk scores	Original research that investigates the incompatibility of optimising for certain fairness metrics or criteria. The criteria behind these fairness metrics are originally proposed in this article and thoroughly investigated (“calibration, and balance for the two classes”); subsequent reviews formalise them as metrics, with minimal detail, which hampers the use of these metrics. Lacks clinical validation at this point.	arXiv	Backward Citation Tracking	Equal Calibration, Balance for Positive Class, Balance for Negative Class	AI	2016	2,343
(45)	Fairness in Machine Learning: A survey	A review of fairness, including metrics, across different domains. Fairness metric introduced and defined mathematically for the first time. Property	ACM Surveys	Backward Citation Tracking	Well Calibration	AI	2021	5,997

		investigation or critical appraisal is not performed. The metric cited (73) as the original paper but it does not actually propose well calibration. Clinical validation is lacking.						
(34)	Equality of opportunity in supervised learning	Original research that proposes criteria for group fairness. These fairness metrics appear to be proposed in this paper for the first time, with mathematical formulation and thorough investigation of properties. This study was selected due to its importance in the field but clinical validation is lacking at this point (2016).	NeurIPS	Backward Citation Tracking	Equal Opportunity Difference, Equalised Odds	AI	2016	5,506
(74)	Algorithmic decision making and the cost of fairness	Original research where the objective is to maximise public interest while satisfying disparities, in a recidivism use case. Metric is introduced with clear motivation and mathematical formulation, as a follow-up to previous studies. This study was selected as the metric was proposed here but clinical validation is lacking at this point (2017).	ACM SIGKDD	Backward Citation Tracking	Predictive Equality	AI	2017	1,645
(75)	Fair prediction with endogenous behavior	Original research with criminal justice as a use case. The study explores the incompatibility of certain fairness metrics or criteria. Equalising disincentives is formally introduced and its implications are studied. Clinical validation lacking.	ACM Economics	Backward Citation Tracking	Equalising Disincentives	AI	2020	44
(76)	Ai-fairness towards activity recognition of older adults	Original research on activity recognition among older adults. Fairness metric is introduced with minimal motivation, referencing, or validation, which is not satisfactory.	ACM EAI	Database Querying	Average Odds Difference	AI	2020	11
(77)	FairEHR-CLP: Towards Fairness-Aware Clinical Predictions with Contrastive Learning in Multimodal Electronic Health Records	Original research that proposes a framework for fair clinical predictions. One of the main contributions is the introduction of a fairness metric, with the goal of "capturing subgroup-specific error rate disparities" but this is done with minimal motivation and validation. "STARR" and "MIMIC" data was used.	arXiv	Database Querying	Average Disparity in Equalised Odds, Error Distribution Disparity Index (EDDI)	AI	2024	6

(78)	Detection and mitigation of algorithmic bias via predictive parity	Original research that focuses on the detection and mitigation of algorithmic bias defined through predictive parity, the metric introduced. This corresponds to the formal statistical test of a fairness criterion that had been proposed elsewhere (44). Formal definition and validation are included. Despite its potential clinical interest, clinical validation is missing at this point.	ACM FaaCT	Database Querying	Predictive Parity	(Applied) Ethics	2023	8
(29)	Ethnic classifications in algorithmic fairness: Concepts, measures and implications in practice	Original research discussing concepts and measures when assessing algorithmic fairness in terms of different ethnicity definitions. Prediction models are built to assess the impact of how race is addressed. Fairness metrics are discussed with limited validation. “SOEP” data was used in a health insurance related task.	ACM FaaCT	Database Querying	NPV Difference, Balanced Accuracy Difference	(Applied) Ethics	2024	3
(79)	Putting fairness principles into practice: Challenges, metrics, and improvements	Original research that provides a case-study of a production classification system, which is somewhat hard to interpret. The authors discuss open questions in implementing equality of opportunity and describe a new fairness metric that takes into account distributional differences. While motivation is clear, further validation of metric behaviour would have been beneficial. This study was selected due to its relevance in defining a new metric, but no clinical validation has been conducted.	ACM AAAI	Backward Citation Tracking	Conditional Equality of Opportunity Gap (EOGap)	(Applied) Ethics	2019	185
(80)	Fairness in criminal justice risk assessments: The state of the art	Review that defines a few fairness metrics in the context of defining algorithmic fairness. These definitions are based on previous studies but are here gathered with more specific implementation details, which the authors argue to be a source of confusion in previous literature. While motivation for metrics is clear, their implications or properties are not studied. The case study is focused on criminal justice risk, and no clinical validation is done at this point.	arXiv	Backward Citation Tracking	Conditional Use Accuracy Equality, Overall Accuracy Gap, Treatment Equality	AI	2021	1,281

(81)	In the name of fairness: assessing the bias in clinical record de-identification	Original research studying the fairness of health record de-identification. Fairness metrics introduced with minimal validation or investigation of properties. "U.S Social Security and Census" datasets are used, with implications in EHR data, according to the authors, although it appears that clinical validation has not been conducted.	ACM FaaCT	Database Querying	Recall Equality Difference, Maximum Difference Recall	(Applied) Ethics	2023	19
(82)	Automated speech analysis for risk detection of depression, anxiety, insomnia, and fatigue: Algorithm Development and Validation Study.	Original research focusing on mental health assessment via speech analysis. Disparity ratios are introduced as a family of fairness metrics, with minimal justification or investigation. Herein, this is reported as "recall disparity ratio" for better specificity and understanding. The data used was collected by the authors and consists of patients' answers regarding their perceived mental and sleep states.	JMIR	Database Querying	Recall Disparity Ratio	Biomedical	2024	1
(83)	Minimax group fairness: Algorithms and experiments	Original research investigating algorithms for "minimax group fairness". The minimum performance measure across subgroups is proposed as a fairness metric, which we herein report as "worst-group recall" for better understanding and feasibility. These metrics are thoroughly studied as fairness constraints. This study was selected for the comprehensive property study of the metric family and fairness criteria, although no clinical validation was conducted.	ACM FaaCT	Backward Citation Tracking	Worst-Group Recall	(Applied) Ethics	2021	123
(84)	Improving fairness in ai models on electronic health records: The case for federated learning methods.	Original research focussing on federated learning fairness using EHR data. The fairness metric is proposed in this context. While motivation is clear in the context of federated learning, metric validation is lacking. "Synthea" (a synthetic dataset) and "MIMIC" datasets were used.	ACM FaaCT	Database Querying	(Recall) Intergroup Standard Deviation	(Applied) Ethics	2023	31
(85)	A unified approach to quantifying algorithmic	Original research proposing that inequality indices can also be used to assess algorithmic fairness. The	ACM SIGKDD	Backward Citation	(Recall) Between-group	AI	2018	354

	unfairness: Measuring individual & group unfairness via inequality indices	study proposes several fairness metrics, derived from GEI, which have limited interpretability and overcomplexity. Clinical validation not performed.		Tracking	Generalised Entropy Index (GEI)			
(86)	Health equity assessment of machine learning performance (HEAL): a framework and dermatology AI model case study	Original research proposing a framework to assess algorithmic fairness in terms of a comparison with previous health outcomes. Dermatology images are used as a case study. While motivation is clear and seems relevant, implications are not sufficiently studied, both from an ethical and statistical point of view. A tele-dermatology dataset from the US and one from cancer clinics in Australia were used.	EClinicalMedicine	Database Querying	(Recall)-HEAL	Biomedical	2024	11
(87)	Can AI help reduce disparities in general medical and mental health care?	Original research involving two case studies to predict ICU mortality and 30-day psychiatric readmission with respect to race, gender, and insurance payer. Error rates were compared across groups, and differences can be reported from these comparisons. This study was included due to its frequent reference in the literature and for being one of the only ones using error rates. “MIMIC-III” data was used.	AMA Journal of Ethics	Backward Citation Tracking	Error Rate Ratio	(Applied) Ethics	2019	401
(52)	A fair federated learning approach to reducing demographic bias in privacy-sensitive classification models	Original research exploring fair classification under federated learning. F1-score parity is introduced under the name “discrimination index”, which seems disconnected from the literature, as “discrimination” typically has a very specific and distinct meaning (relating to, e.g, AUC). The metric’s properties are not studied. No clinical validation conducted.	IEEEXplore	Backward Citation Tracking	F1 Parity (“Discrimination Index”)	AI	2020	133
(88)	The advantages of the Matthews correlation coefficient (MCC) over F1 score and accuracy in binary classification evaluation.	Original investigation that discusses the advantages of MCC over accuracy and F1-score as metrics. Although this study does not specifically define MCC parity, it greatly elaborates on MCC itself and is often cited to support the use of MCC parity. Validation is conducted in synthetic datasets and one genomics scenario. Better validation of this	BMC Genomics	Backward Citation Tracking	Matthew's Correlation Coefficient (MCC) Parity	Biomedical	2020	5,497

		fairness metric is missing for clinical prediction models.						
(89)	Understanding algorithmic fairness for clinical prediction in terms of subgroup net benefit and health equity	Original research discussing fairness in clinical prediction models using net benefit. The fairness metric is derived mathematically and its properties are thoroughly studied. Utility is defined in terms of true negatives, which may need to be tweaked depending on the clinical application. Empirical validation is adequate and metric seems promising and relevant. A diabetes prognostic model and a lung cancer screening algorithm are used, which are relevant. “UK Biobank” data is used.	arXiv	Grey Literature	Subgroup net benefit	Biomedical	2024	0

Supplemental Table 2.6. Summary statistics of communities, venues, and article identification method for each included article

Article Information	N
**Community**	
(Applied) Ethics	13
AI	20
Biomedical	9
**Venue / Journal**	
ACM AAAI	3
ACM AIES	1
ACM EAI	1
ACM Economics	1
ACM FaaCT	7
ACM SIGKDD	4
ACM Surveys	1
AMA Journal of Ethics	1
AMIA	1
arXiv	8
BMC Genomics	1
eBioMedicine	1
eClinicalMedicine	1
ICLR	1
ICML	1
IEEEXplore	4
JAMA Network Open	1
JMIR	1
medRxiv	1
NeurIPS	2
Scientific Data	1
**Article Identification Method**	
Backward Citation Tracking	22
Database Querying	19
Grey Literature	1

Fairness metrics were proposed and defined across three main research communities, with most articles originating from “AI” venues (n = 20), followed by “Applied Ethics” (n = 13), and “Biomedical research” (n = 9).

Critical Appraisal of Fairness Metrics in Clinical Predictive AI

Supplementary Material 3: Formal definitions of identified fairness metrics

João Matos, MSc[1], Ben Van Calster, PhD[2,3*], Leo Anthony Celi, MD[4,5,6], Paula Dhiman, PhD[1], Judy Wawira Gichoya, MD[7], Richard D. Riley, PhD[8,9*], Chris Russell, PhD[10], Sara Khalid, PhD[1], Gary S. Collins, PhD[1*]

[1]Centre for Statistics in Medicine, Nuffield Department of Orthopaedics, Rheumatology and Musculoskeletal Sciences, University of Oxford, Oxford, UK
[2]Department of Development and Regeneration, KU Leuven, Leuven, Belgium
[3]Department of Biomedical Data Science, Leiden University Medical Centre, Leiden, Netherlands
[4]Beth Israel Deaconess Medical Center, Boston, MA, USA
[5]Laboratory for Computational Physiology, Massachusetts Institute of Technology, Cambridge, MA, USA
[6]Department of Biostatistics, Harvard T H Chan School of Public Health, Boston, MA, USA
[7]Department of Radiology and Imaging Sciences, Emory University, Atlanta, GA, USA
[8]Department of Applied Health Sciences, School of Health Sciences, College of Medicine and Health, University of Birmingham, Birmingham, UK
[9]National Institute for Health and Care Research (NIHR) Birmingham Biomedical Research Centre, Birmingham, UK
[10]Oxford Internet Institute, University of Oxford, Oxford, UK

[*]Full Professor

Contents

1 Performance Evaluation Metrics **27**
1.1 Notation . . . 27
1.2 Metrics . . . 27
1.2.1 Probability-based . . . 27
1.2.2 Threshold-dependent . . . 29

2 Performance-independent fairness metrics **30**
2.1 Probability-based . . . 30
2.1.0.1 Mean or Average Score Parity. . . . 30
2.1.0.2 Unsupervised Ranking Fairness (URF) . . . 30
2.1.0.3 Regression Demographic Parity . . . 30
2.1.0.4 Survival Group Fairness . . . 31
2.1.0.5 Censoring-Based Group Fairness . . . 31
2.1.0.6 Survival Intersectional Fairness . . . 32
2.1.0.7 Survival Individual Fairness . . . 32
2.1.0.8 Censoring-Based Individual Fairness . . . 33
2.2 Threshold-dependent . . . 33
2.2.0.1 Statistical Parity . . . 33
2.2.0.2 Multi-group Demographic Parity . . . 33
2.2.0.3 Generalised Demographic Parity (GDP) . . . 34
2.2.0.4 Statistical Parity Subgroup Fairness . . . 34
2.2.0.5 Conditional Statistical Parity . . . 34
2.2.0.6 Disparate Impact . . . 35
2.2.0.7 Differential Fairness . . . 35

3 Performance-dependent fairness metrics **36**
3.1 Probability-based . . . 36
3.1.1 Discrimination . . . 36
3.1.1.1 AUROC Parity . . . 36
3.1.1.2 AUPRC Parity . . . 36
3.1.1.3 Cross-Area Under the ROC Curve (xAUROC) . . . 36
3.1.1.4 xAUROC Disparity . . . 37
3.1.1.5 Subgroup AUROC (sAUROC) . . . 37
3.1.1.6 Pairwise Ranking Fairness (PRF) . . . 37

3.1.1.7 PRF Disparity (ΔPRF) . . . 37
3.1.1.8 Equity-Scaled Recall (ES-Recall) . . . 38
3.1.1.9 Mean Performance-Scaled Disparity (PSD) for AUROC . . . 38
3.1.1.10 Maximum Performance-Scaled Disparity (PSD) for AUROC . . . 39
3.1.1.11 Concordance Imparity (CI) . . . 39
3.1.1.12 AUROC Parity Ratio . . . 40
3.1.2 Calibration . . . 40
3.1.2.1 Expected Calibration Error (ECE) Parity . . . 40
3.1.2.2 Calibration-in-the-large Parity . . . 40
3.1.2.3 Absolute Calibration Error Parity . . . 40
3.1.2.4 Equal Calibration . . . 41
3.1.2.5 Well Calibration . . . 41
3.1.3 Overall . . . 41
3.1.3.1 Brier Score Parity . . . 41
3.1.3.2 Log-Loss Parity . . . 42
3.1.3.3 Earth Mover's Distance (EMD) for equivalent separation . . . 42
3.1.3.4 Balance for Positive Class . . . 42
3.1.3.5 Balance for Negative Class . . . 43
3.2 Threshold-dependent . . . 43
3.2.1 Partial . . . 43
3.2.1.1 Equal Opportunity Difference . . . 43
3.2.1.2 Predictive Equality. . . . 43
3.2.1.3 Equalised Odds . . . 43
3.2.1.4 Equalising Disincentives . . . 44
3.2.1.5 Average Odds Difference . . . 44
3.2.1.6 Average Disparity in Equalised Odds . . . 44
3.2.1.7 Predictive Parity . . . 45
3.2.1.8 NPV Difference . . . 45
3.2.1.9 Conditional Equality of Opportunity Gap (EOGap) . . . 45
3.2.1.10 Conditional Use Accuracy Equality. . . . 46
3.2.1.11 Recall Equality Difference . . . 46
3.2.1.12 Recall Disparity Ratio . . . 46
3.2.1.13 Worst-Group Outcome . . . 46
3.2.1.14 Maximum Difference Recall . . . 47
3.2.1.15 Recall Intergroup Standard Deviation (Recall-ISD) . . . 47
3.2.1.16 Recall Coefficient of Variation (Recall-CV) . . . 47
3.2.1.17 Between-group Generalised Entropy Index (GEI) . . . 48
3.2.1.18 Recall-HEAL . . . 48
3.2.2 Summary . . . 49
3.2.2.1 Overall Accuracy Gap. . . . 49
3.2.2.2 Error Rate Ratio . . . 49
3.2.2.3 Balanced Accuracy Difference . . . 49
3.2.2.4 F1 Parity . . . 50
3.2.2.5 Matthew's Correlation Coefficient (MCC) Parity . . . 50
3.2.2.6 Treatment Equality . . . 50
3.2.2.7 Error Distribution Disparity Index (EDDI) . . . 50
3.2.3 Clinical Benefit . . . 51
3.2.3.1 Subgroup Net Benefit . . . 51

Preliminaries

In this section we introduce the notation to be used in this document, as well performance metrics that may be used to derive other fairness metrics.

1 Performance Evaluation Metrics

1.1 Notation

Let A denote the sensitive or protected attribute. This attribute can take on various forms depending on the context:

- it can be *dichotomized*, where there are two groups, such as in the case of sex, with $A = 0$ representing one group (e.g., male) and $A = 1$ representing the other group (e.g., female).
- A may represent multiple groups, such as race or ethnicity, where $A \in \{0, 1, 2, \ldots, K\}$ for K distinct groups.
- A can also take continuous values, such as age, where $A \in \mathbb{R}_0^+$, representing a range of numerical values.

The true outcome label is denoted by Y. The model's estimated probability is represented by $\hat{p} = f(X)$, where f is the model that maps the set of features X to a predicted outcome. Upon defining a threshold τ, estimated probabilities $\hat{p}$ can be transformed into a predicted classification, $\hat{Y}$. "Legitimate features" used in the model are represented by L and these are the ones that are agreed to be "true" probability modifiers.

The size of the groups can be indicated by n_0, the number of patients in the group where $A = 0$ (if dichotomized), and n_1, the number of patients in the group where $A = 1$. For cases with multiple groups or continuous values, the size of specific subgroups can be represented similarly.

For a full list of notation and definitions, refer to Table 1.1.

Table I: Notation and definitions.

**Symbol**	**Description**
A	Sensitive or protected attribute
n_0	Number of patients with $A = 0$
n_1	Number of patients with $A = 1$
X	Set of features
L	Legitimate features
Y	True class
$\hat{Y}$	Predicted class
$\hat{p}$	Estimated probability
f	Model
τ	Decision threshold for binary model

1.2 Metrics

In evaluating prediction models, understanding performance metrics is crucial. In the context of fairness, these metrics serve as inputs for performance-dependent fairness metrics.

1.2.1 Probability-based

These metrics do not set any decision threshold and work with the estimated probabilities outputted by a predictive model.

- **AUROC** (Area Under the Receiver Operating Characteristic [ROC] Curve) The ROC curve plots the TPR against the FPR for various decision thresholds for a binary classifier. The AUROC represents the area under this curve and can be expressed as:

$$\text{AUROC} = \int_0^1 \text{TPR}(\text{FPR})\, d(\text{FPR}),$$

where TPR is a function of FPR, and the integration is performed over the range of FPR values from 0 to 1 that is obtained via varying decision thresholds for a binary classifier. (Hanley and McNeil, 1982) Alternatively, AUROC can also be expressed as:

$$\text{AUROC} = P[\hat{p}(x^+) > \hat{p}(x^-)] = \frac{1}{n_1 n_0} \sum_{i:Y_i=1} \sum_{j:Y_j=0} \mathbb{I}[\hat{p}(X_i) > \hat{p}(X_j)],$$

where $\hat{p}(x)$ is the estimated probability assigned to an instance x, x^+ and x^- denote a randomly chosen positive and negative instance, respectively. n_1 and n_0 represent the number of positive and negative instances in the dataset, and $\mathbb{I}$ is the indicator function that evaluates to 1 when the condition $\hat{p}(X_i) > \hat{p}(X_j)$ is true, and 0 otherwise.

This formulation highlights that AUROC measures the probability that a randomly chosen positive instance will be ranked higher than a randomly chosen negative instance (Kallus and A. Zhou, 2019; Cui *et al.*, 2023).

- **AUPRC** (Area Under the Precision-Recall [PR] Curve) The PR curve plots Precision (PPV) against Recall (TPR) for various thresholds. The AUPRC represents the area under this curve and is given by:

$$\text{AUPRC} = \int_0^1 \text{PPV}(\text{TPR})\, d(\text{TPR})$$

  where PPV is a function of TPR, and the integration is carried out over the range of TPR values from 0 to 1.

- **ECE** (Expected Calibration Error) ECE quantifies the calibration of a probabilistic classifier by measuring the weighted average of the differences between bin accuracy and bin confidence across M bins. The bins partition the probability interval $[0, 1]$ into equal-sized intervals. (Naeini *et al.*, 2015; Guo *et al.*, 2017) ECE is defined as:

$$\text{ECE} = \sum_{m=1}^{M} \frac{|B_m|}{n} \left|\text{acc}(B_m) - \text{conf}(B_m)\right|,$$

  where:

  - B_m: Set of instances whose estimated probabilities fall within the m-th bin, $B_m = \{i \mid \hat{Y}_i \in I_m\}$, and $I_m = \left(\frac{m-1}{M}, \frac{m}{M}\right]$.
  - $|B_m|$: Number of instances in the m-th bin.
  - n: Total number of instances.
  - $\text{acc}(B_m)$: Accuracy within bin B_m, given by:

$$\text{acc}(B_m) = \frac{1}{|B_m|} \sum_{i \in B_m} \mathbb{I}(\hat{Y}_i = Y_i),$$

    where $\mathbb{I}(\hat{Y}_i = Y_i)$ is an indicator function that equals 1 if the predicted label matches the true label and 0 otherwise.
  - $\text{conf}(B_m)$: Confidence within bin B_m, given by:

$$\text{conf}(B_m) = \frac{1}{|B_m|} \sum_{i \in B_m} \hat{p}_i,$$

    where S_i is the estimated probability for instance i, as defined in Table 1.1.

  A perfectly calibrated model has $\text{acc}(B_m) = \text{conf}(B_m)$ for all m, resulting in ECE $= 0$. Larger ECE values indicate greater calibration errors, with the calibration gap $|\text{acc}(B_m) - \text{conf}(B_m)|$ contributing to the overall error.

- **Brier Score** The Brier Score evaluates the accuracy of probabilistic predictions by measuring the mean squared difference between estimated probabilities ($\hat{p}$) and observed outcomes (Y). (Brier, 1950) It is defined as:

$$\text{Brier Score} = \frac{1}{n}\sum_{i=1}^{n}\left(\hat{p}_i - Y_i\right)^2,$$

  Brier score can range from 0 to 1, where 0 indicates better model calibration and discrimination, as predictions are closer to the true outcomes.

1.2.2 Threshold-dependent

Based on an estimated probability, one can set a threshold and obtain a predicted class. For the binary case:

- **True Positive (TP):** Correctly predicted positive class: $\hat{Y} = 1$ and $Y = 1$.
- **False Positive (FP):** Incorrectly predicted positive class: $\hat{Y} = 1$ and $Y = 0$.
- **False Negative (FN):** Incorrectly predicted negative class: $\hat{Y} = 0$ and $Y = 1$.
- **True Negative (TN):** Correctly predicted negative class: $\hat{Y} = 0$ and $Y = 0$.

Table II: Confusion matrix depicting prediction outcomes: TP, FP, FN, and TN.

	$\hat{Y} = 1$	$\hat{Y} = 0$
$Y = 1$	TP	FN
$Y = 0$	FP	TN

Threshold-dependent metrics: performance metrics can then be computed based on measures computed from confusion matrix:

- **TPR (Sensitivity or Recall):** True positive rate, $P(\hat{Y} = 1 \mid Y = 1) = \frac{TP}{TP+FN}$
- **FNR:** False negative rate, $P(\hat{Y} = 0 \mid Y = 1) = \frac{FN}{TP+FN} = 1 - \text{TPR}$
- **FPR:** False positive rate, $P(\hat{Y} = 1 \mid Y = 0) = \frac{FP}{TN+FP}$
- **TNR (Specificity):** True negative rate, $P(\hat{Y} = 0 \mid Y = 0) = \frac{TN}{TN+FP} = 1 - \text{FPR}$
- **PPV (Precision):** Positive predictive value, $P(Y = 1 \mid \hat{Y} = 1) = \frac{TP}{TP+FP}$
- **FDR:** False discovery rate, $P(Y = 0 \mid \hat{Y} = 1) = \frac{FP}{TP+FP} = 1 - \text{PPV}$
- **NPV:** Negative predictive value, $P(Y = 0 \mid \hat{Y} = 0) = \frac{TN}{TN+FN}$
- **FOR:** False omission rate, $P(Y = 1 \mid \hat{Y} = 0) = \frac{FN}{TN+FN} = 1 - \text{NPV}$
- **Accuracy:** Overall correct predictions, $P(\hat{Y} = Y) = \frac{TP+TN}{TP+TN+FP+FN}$
- **Balanced Accuracy:** Mean of recall (TPR) and specificity (TNR), $\text{BACC} = \frac{TPR+TNR}{2}$
- **F1-Score:** Harmonic mean of (PPV) precision and (TPR) recall, $\text{F1-Score} = 2 \cdot \frac{\text{PPV} \cdot \text{TPR}}{\text{PPV}+\text{TPR}}$
- **Matthew's Correlation Coefficient (MCC):** A balanced measure of classification performance, accounting for TP, TN, FP, and FN.

$$\text{MCC} = \frac{\text{TP}\cdot\text{TN} - \text{FP}\cdot\text{FN}}{\sqrt{(\text{TP}+\text{FP})(\text{TP}+\text{FN})(\text{TN}+\text{FP})(\text{TN}+\text{FN})}}$$

  MCC ranges from -1 (completely incorrect predictions) to $+1$ (perfect predictions), with 0 indicating random performance. (Chicco and Jurman, 2020)

Fairness Metrics

Herein, we report all the fairness metrics that were identified in scoping review process. For each metric, we report the mathematical definition, how to interpret the metric, and potential implications. N.B. these metrics are not necessarily recommended but the ones that were found in the literature.

2 Performance-independent fairness metrics

These metrics are solely based on the model's output and do not assess performance (against a ground-truth).

2.1 Probability-based

2.1.0.1 Mean or Average Score Parity.

(Coston *et al.*, 2019) This metric ensures that the average estimated probability $\hat{p}$ (score), is equal across protected groups A.

Mathematical Definition:

$$\mathcal{D}_{\text{mean parity}} = E[\hat{p}|A = 1] - E[\hat{p}|A \neq 1] \quad (1)$$

Interpretation: Mean or Average Score Parity assesses whether the average estimated probability of an expected outcome (e.g., treatment assignment or diagnosis) is consistent across groups. A higher level of parity indicates reduced dependence of the classifier's outputs on protected attributes. A model is considered to satisfy this metric if the expected value of the $\hat{p}$ remains consistent across all groups.

2.1.0.2 Unsupervised Ranking Fairness (URF)

This metric measures the disparity in ranking probabilities between two protected groups. (Cui *et al.*, 2023) URF evaluates whether the two groups have equal probabilities of ranking higher than one another, serving as a concordance statistic for the two protected groups, without depending on performance.

Mathematical Definition:

$$\text{URF}(a, b) = \left| P[y^{A=1} > y^{A\neq 1}] - P[y^{A\neq 1} > y^{A=1}] \right| \quad (2)$$

Interpretation: URF requires that the two protected groups $A = 1$ and $A \neq 1$ have the same probability of ranking higher than the other. A URF value of 0 indicates perfect ranking fairness.

2.1.0.3 Regression Demographic Parity

Regression Demographic Parity (DP_{reg}) is an extension of average score parity to scenarios where the sensitive attribute has more than two categories. (Nguyen *et al.*, 2023)

Mathematical Definition:

$$DP_{reg} = \frac{1}{C(|A|, 2)} \sum_{s=1}^{|A|-1} \sum_{a'=a+1}^{|A|} |\mathbb{E}[\hat{p} \mid A = a] - \mathbb{E}[\hat{p} \mid A = a']| \,, \quad (3)$$

where:

- A: Set of all subpopulations (as before),
- a: A specific subpopulation within A,
- $|A|$: Cardinality of A, representing the total number of subpopulations,
- $C(|A|, 2)$: Binomial coefficient indicating the number of distinct pairs of subpopulations,
- $E[\hat{p}]$: Expected probability estimation by the model.

Interpretation: This metric evaluates whether the average predicted outcomes do not differ across subpopulations. This is similar to the Mean Score Parity. A lower $\mathrm{DP}_{\mathrm{reg}}$ value indicates greater parity, while higher values suggest disparities between subpopulations.

2.1.0.4 Survival Group Fairness

This metric measures the worst-case deviation of the per-group expected hazard function $\bar{h}_\beta(a)$ from the population average hazard. (Keya *et al.*, 2020)

Mathematical Definition:

$$F_g = \max_{a \in A} \left| \bar{h}_\beta(a) - E[\bar{h}_\beta(X)] \right|, \tag{4}$$

where:

$$\bar{h}_\beta(a) \triangleq \int_X \exp(\beta^\top X) p(X \mid a), \tag{5}$$

and:

- $\bar{h}_\beta(a)$: Per-group expected hazard function for group a,
- $E[\bar{h}_\beta(X)]$: Population average hazard,
- $p(X \mid a)$: Conditional probability distribution of features X given a,
- $\exp(\beta^\top X)$: Hazard function component.

Interpretation: This metric captures the maximum disparity in the expected hazard function across protected groups. A lower F_g value indicates "greater fairness", with similar expected hazards across all groups.

Further, the naming convention used in the original article appears to be decontextualised from the broader landscape of fairness metrics, as "group fairness" is a type of fairness and not a metric on its own.

2.1.0.5 Censoring-Based Group Fairness

Censoring-Based Group Fairness ($F_{CG}(t)$) extends the concept of group fairness by incorporating censoring information. It compares survival probability estimation between groups of censored and uncensored individuals (Hu and G. H. Chen, 2024).

Mathematical Definition:

$$F_{CG}(t) \triangleq \frac{1}{|N_c| \times |N_{uc}|} \sum_{g \in \mathcal{G}} \sum_{i \in N_{c,g}, j \in N_{uc,g} \text{ s.t. } Y_j \geq Y_i} \left[\left| \hat{p}(t \mid X_i) - \hat{p}(t \mid X_j) \right| - \gamma \|X_i - X_j\| \right]_+ \tag{6}$$

where:

- $N_{c,g}$: Index set of censored data within group g,
- $N_{uc,g}$: Index set of uncensored data within group g,
- $\mathcal{G}$: Set of all groups,
- $\hat{p}(t \mid X)$: Estimated survival probability at time t for patient X,
- γ: Scale factor for feature distance, predefined,
- $[x]_+ = \max(0, x)$: Rectified linear function.

Interpretation: $F_{CG}(t)$ provides a fairness evaluation at the group level for survival analysis tasks. As with the individual censoring-based fairness metric, the selection of γ influences the results: a larger γ reduces the likelihood of $F_{CG}(t)$ exceeding zero. The final score is calculated as the average of $F_{CG}(t)$ values at the 25th, 50th, and 75th percentiles of observed times in the test data.

Implications: This metric measures the fairness of predictions within groups, ensuring that the survival probabilities for censored and uncensored individuals with similar features remain comparable. $F_{CG}(t)$ accounts for the impact of censoring in group-level comparisons.

2.1.0.6 Survival Intersectional Fairness

This metric evaluates disparities in the log-ratios of per-group hazard functions between intersectional subgroups, thereby addressing vulnerabilities that may arise at these intersections. (Keya *et al.*, 2020)

Mathematical Definition:

$$F_\epsilon = \max_{s_i \in A, s_j \in A} \left| \log \bar{h}_\beta(s_i) - \log \bar{h}_\beta(s_j) \right|, \tag{7}$$

where:

- $A = S_1 \times S_2 \times \cdots \times S_K$: Space of intersectional subgroups defined by the Cartesian product of protected attribute sets,
- s_i, s_j: Specific intersectional subgroups within A (e.g., women aged 20–30),
- $\bar{h}_\beta(s)$: Expected hazard function for subgroup s.

Interpretation: This metric computes the maximum disparity in the log-ratios of hazard functions across all pairs of intersectional subgroups. A lower F_ϵ value indicates reduced differences across vulnerable intersectional groups.

Implications: Similar to previous metrics that have been extended to survival analysis, this definition of a single metric as "intersectional fairness" is quite narrow and does not fully capture the breadth and complexity of the concept.

2.1.0.7 Survival Individual Fairness

Survival Individual Fairness measures whether a model produces similar outcomes for individuals deemed similar based on a predefined distance metric. In the context of survival models, this fairness criterion penalises differences in estimated hazard scores exceeding the distance between individuals in feature space. (Keya *et al.*, 2020)

Mathematical Definition:

$$F_i = \sum_{i=1}^{N^{(test)}} \sum_{j=i+1}^{N^{(test)}} \max(0, |\bar{h}_\beta(\mathbf{x}_i) - \bar{h}_\beta(\mathbf{x}_j)| - D(\mathbf{x}_i, \mathbf{x}_j)), \tag{8}$$

where:

- $\bar{h}_\beta(\mathbf{x}) = \exp(\beta^\top \mathbf{x})$: Hazard function with the base hazard $h_0(t)$ excluded, as it is not individual-specific,
- $D(\mathbf{x}_i, \mathbf{x}_j)$: Distance metric (e.g., Euclidean distance) between individuals $\mathbf{x}_i$ and $\mathbf{x}_j$, encoding similarity,
- $N^{(test)}$: Number of test set samples.

Interpretation: This metric penalises models for which predicted hazard scores for similar individuals differ by more than their defined similarity distance. The lower F_i, the lower such difference is.

Implications: It requires access to a meaningful distance metric.

2.1.0.8 Censoring-Based Individual Fairness

Censoring-Based Individual Fairness ($F_{CI}(t)$) builds upon survival individual fairness by incorporating censoring information. It aims for patients with similar features and outcomes to have similar predictions, even when some patients are censored.(Hu and G. H. Chen, 2024)

Mathematical Definition:

$$F_{CI}(t) \triangleq \frac{1}{|N_c| \times |N_{uc}|} \sum_{i \in N_c, j \in N_{uc} \text{ s.t. } Y_j \geq Y_i} \left[\left| \hat{p}(t \mid X_i^{\text{test}}) - \hat{p}(t \mid X_j^{\text{test}}) \right| - \gamma \| X_i^{\text{test}} - X_j^{\text{test}} \| \right]_+ \quad (9)$$

where:

- N_c: Index set of censored data,
- N_{uc}: Index set of uncensored data,
- $\hat{p}(t \mid X)$: Estimated survival probability at time t for patient X,
- γ: Scale factor for the feature distance, predefined (e.g., 0.01),
- $[x]_+ = \max(0, x)$: Rectified linear function.

Interpretation: $F_{CI}(t)$ evaluates fairness in survival analysis by addressing censoring biases. However, the choice of γ influences results: higher γ makes it easier for $F_{CI}(t)$ to approach zero. This metric is sensitive to the definition of similarity and the selection of t, typically evaluated at the 25th, 50th, and 75th percentiles of observed times in the test data, with their average used as the final score.

Implications: This metric ensures that censored and uncensored patients with similar features and outcomes have similar survival probability predictions. The γ parameter penalises excessive differences in predictions relative to feature distances.

2.2 Threshold-dependent

2.2.0.1 Statistical Parity

Also known as *"demographic parity"* or *"statistical parity difference"* or *"thresholded score parity"* (in contrast with average score parity).(Zemel *et al.*, 2013) Can be seen as a threshold-dependent version of the Mean Score Parity 2.1.0.1.

Mathematical Definition:

$$\mathcal{D}_{\text{statistical parity}} = \left| P[\hat{Y} = 1 | A = 1] - P[\hat{Y} = 1 | A \neq 1] \right| \quad (10)$$

Interpretation: A lower value of this metric indicates a smaller discrepancy in positivity rates between groups, signalling greater "fairness". Demographic parity, therefore, assesses the extent to which the rate of positive predictions differs between the two groups. *Note that this metric does not utilise the ground-truth Y.*

Implications: One drawback of measures like demographic parity is that a classifier with 100% accuracy may be labelled as unfair if the base rates (i.e., the actual positive outcome proportions) differ significantly between groups. Additionally, to satisfy demographic parity, patients with similar profiles might have to be treated differently if they belong to different groups, resulting in disparate error rates. On the other hand, one must note that to achieve demographic parity, it is only necessary to create a classifier that outputs the same predictions (positive or negative) for all patients.(Mittelstadt *et al.*, 2023)

2.2.0.2 Multi-group Demographic Parity

The definition of statistical or demographic parity presented in 2.2.0.1 is limited to a dichotomized view of sensitive attributes. Herein, a generalisation for categorical sensitive attributes is presented, which is still equivalent for the binary version. (Jiang *et al.*, 2022)

Mathematical Definition:

$$\Delta DDP = \sum_{a\in\mathcal{A}} \left|\mathbb{E}[\hat{Y}|A = A] - \mathbb{E}[\hat{Y}]\right| \tag{11}$$

Interpretation: Same as original demographic parity.
Implications: Same as original demographic parity.

2.2.0.3 Generalised Demographic Parity (GDP)

Although demographic parity has been widely used to evaluate the prediction bias, even with the multi-group extension 2.2.0.2, it is still inapplicable for continuous sensitive attributes since the data samples cannot be directly divided into several distinctive groups based on the sensitive attributes. Assuming continuous sensitive attributes $A \in [0, 1]$, (Jiang *et al.*, 2022) propose GDP to extend tractable DP for continuous sensitive attributes.

$$\Delta\text{GDP} = \int_0^1 \left|\mathbb{E}[\hat{Y}|A = a] - \mathbb{E}[\hat{Y}]\right| P_S(A = a)\,\mathrm{d}s \tag{12}$$

where $P_A(A = a)$ is the probability density function of the sensitive attribute

This can be implemented via histogram GDP estimation and kernel GDP estimation methods based on different group strategies. (Jiang *et al.*, 2022)

Interpretation: Similar to the original demographic parity.

2.2.0.4 Statistical Parity Subgroup Fairness

Statistical Parity Subgroup Fairness extends the concept of statistical parity to account for specific subgroups within a collection of protected groups $\mathcal{G}$. It computes the disparity in positive prediction rates between the overall population and specific subgroups, weighted by the subgroup's representation. (Kearns *et al.*, 2018; Foulds *et al.*, 2020)

Mathematical Definition:

$$\mathcal{D}_{subgroupstatisticalparity} = \left|P\left(\hat{Y} = 1\right) - P\left(\hat{Y} = 1 \mid g(A) = 1\right)\right| \times P(g(A) = 1), \tag{13}$$

where:

- $\mathcal{G}$: Collection of protected group indicators A,
- $g(A)$: Indicator function for subgroup membership, $g(A) = 1$ if A belongs to subgroup g.

Interpretation: Smaller reported differences indicate more similarity in statistical parity across subgroups.

Implications: While addressing subgroup fairness, this metric may underrepresent minority groups due to the proportional weighting term $P_\theta(g(A) = 1)$, which can dilute the fairness focus on smaller subgroups.

2.2.0.5 Conditional Statistical Parity

An extension of statistical parity. This metric controls for a limited set of "legitimate" features, ensuring that an equal proportion of individuals is selected from each group based on these factors. "Legitimate" features are ones that are independent of the protected attributes.(Corbett-Davies *et al.*, 2017)

Mathematical Definition:

$$\mathcal{D}_{\text{conditional parity}} = \left|P[\hat{Y} = 1|A = 1, X = l] - P[\hat{Y} = 1|A \neq 1, X = l]\right| \tag{14}$$

where l represents a subset of features that are considered "legitimate". In practice, computing this metric could mean using different *strata* to segment the study population.

Interpretation: Conditional statistical parity assesses fairness by controlling for specific, legitimate features and comparing positive prediction rates across protected groups within each subset of these features. A lower value reflects a smaller disparity in positivity rates.

Implications: Applying this metric requires careful definition of which features are considered legitimate, a challenging task since few features are entirely independent of sensitive attributes. As it is nontrivial to ascertain which features are "legitimate", the selection of legitimate features can introduce subjectivity and complexity to the analysis. However, if done right, the strata definition can make this metric highly relevant in assessing fairness.

2.2.0.6 Disparate Impact

This metric was developed to quantify the legal concept of disparate impact, requiring that the ratio of positive prediction rates across groups remains high.(Feldman *et al.*, 2015) This ensures that the proportion of positive predictions (e.g., acceptances or access to care) is similar between groups.

Its origins are in legal fairness considerations for selection procedures which sometimes use an 80% rule to define whether a process has disparate impact (ratio smaller than 0.8) (Feldman *et al.*, 2015).

Mathematical Definition:

$$\mathcal{D}_{\text{disparate impact}} = \frac{P[\hat{Y} = 1 | A \neq 1]}{P[\hat{Y} = 1 | A = 1]} \tag{15}$$

Interpretation: A higher value of this ratio indicates greater similarity in positive prediction rates across groups, signalling increased fairness.

Alternatively, this metric can be reformulated as what we call an "inverted disparate impact" measure (J. Zhang *et al.*, 2024):

$$\mathcal{D}_{\text{inverted D.I.}} = 1 - \min(\mathcal{D}_{\text{impact}}, 1/\mathcal{D}_{\text{impact}}) \tag{16}$$

This reformulation ensures that a perfectly fair model has a value of 0, rather than 1.

Implications: This measure aligns with the "80 percent rule" in disparate impact law, which mandates that the acceptance rate for any protected group should be at least 80% of that for the group with the highest acceptance rate. It can be seen as a measure of unintentional downstream "bias". However, it may not fully capture other fairness aspects, as it focuses solely on prediction rate similarity and does not consider ground-truth Y.

2.2.0.7 Differential Fairness

The differential fairness metric is presented as a hybrid measurement, at "an intersectional definition of fairness", which measures the similarity of model classification across combinations of sensitive attributes. On the other hand, as a notion/criterion (not a metric) it ensures that the likelihood of a prediction outcome is bounded within a specified range $(e^{-\epsilon}, e^{\epsilon})$ for all pairs of sensitive attribute tuples. According to the authors, this metric can be particularly useful in contexts with multiple sensitive attributes (e.g., race and sex). (Wan *et al.*, 2023; Foulds *et al.*, 2020)

Mathematical Definition:

$$\mathcal{D}_{DifferentialFairness} = \log\left(\frac{P(\hat{Y} = 1 \mid A = s_i)}{P(\hat{Y} = 1 \mid A = s_j)}\right), \tag{17}$$

and the criterion of "differential fairness" is met when

$$e^{-\epsilon} \leq \mathcal{D}_{DifferentialFairness} \leq e^{\epsilon}, \tag{18}$$

where:

- A: Sensitive attributes (e.g., combinations like {male, black} or {female, white}),
- s_i, s_j: Specific tuples of sensitive attribute values,
- ϵ: Differential fairness parameter controlling the allowable deviation.

Interpretation: A lower ϵ value indicates tighter bounds and stronger consistency across all combinations of sensitive attributes. Higher values of ϵ allow greater deviations.

3 Performance-dependent fairness metrics

3.1 Probability-based

3.1.1 Discrimination

3.1.1.1 AUROC Parity

The AUROC Parity measures the difference in AUROC between two subgroups defined by the sensitive attribute A. (Balagopalan *et al.*, 2022; Cheng *et al.*, 2021)

Mathematical Definition:

$$\mathcal{D}_{\text{AUROC}} = \left|\text{AUROC}_{A=1} - \text{AUROC}_{A\neq 1}\right|. \tag{19}$$

Interpretation: A parity value of 0 indicates no differences in AUROC across groups.

Implications: Minimising this metric means that similar discrimination across groups is being aimed for. Performance may vary naturally across groups and that must be considered when taking conclusions from this metric.

3.1.1.2 AUPRC Parity

The AUPRC Parity measures the difference in AUPRC between two subgroups defined by the sensitive attribute A. (S. Pfohl *et al.*, 2019)

Mathematical Definition:

$$\mathcal{D}_{\text{AUPRC}} = \left|\text{AUPRC}_{A=1} - \text{AUPRC}_{A\neq 1}\right|. \tag{20}$$

Interpretation: A parity value of 0 indicates no differences in AUPRC across groups.

3.1.1.3 Cross-Area Under the ROC Curve (xAUROC)

The Cross-Receiver Operating Characteristic (xROC) curve captures group-level disparities in misranking errors. It can be defined as:

$$\text{xROC}(\tau; y, a, b) = \left(P[y > \tau \mid A = a, Y = 0], P[y > \tau \mid A = b, Y = 1]\right),$$

where τ is the threshold, A is the sensitive attribute, Y is the true outcome, and y the probability estimated by the model. The xROC curve plots the TPR of group b (positive instances) against the FPR of group a (negative instances) across various thresholds.

The Cross-Area Under the ROC Curve (xAUROC) quantifies these disparities by measuring the probability that a randomly chosen positive instance ($Y = 1$) from one group is ranked higher than a randomly chosen negative instance ($Y = 0$) from another group. (Kallus and A. Zhou, 2019; Cui *et al.*, 2023)

Mathematical Definition:

$$\text{xAUROC}(a, b) = P[y_1^a > y_0^b] = \frac{1}{n_1^a n_0^b} \sum_{i: i\in a, Y_i=1} \sum_{j: j\in b, Y_j=0} \mathbb{I}\left[y(X_i, a) > y(X_j, b)\right], \tag{21}$$

Additionally, two metrics that expand upon xAUROC assess group-specific ranking disparities. The **Positive xAUROC** (xAUC_k^1) quantifies the probability that positive instances of group k are ranked above negative instances of all other groups, while the **Negative xAUROC** (xAUC_k^0) measures the probability that negative instances of group k are ranked below positive instances of all other groups.(S. R. Pfohl *et al.*, 2021)

Interpretation: The xAUROC provides a probabilistic interpretation of cross-group ranking fairness. A value of 0.5 indicates no ranking disparity between groups a and b, while deviations from 0.5 highlight misranking errors between the groups.

Implications: Motivation and theoretical underpinning are not fully clear, as empiricial studies are lacking.

3.1.1.4 xAUROC Disparity

The xAUROC Disparity measures the difference in xAUROC values between two sensitive groups a and b. (S. R. Pfohl *et al.*, 2021; Kallus and A. Zhou, 2019; Cui *et al.*, 2023)

Mathematical Definition:

$$\Delta\text{xAUROC(a,b)} = \left|P[y_1^a > y_0^b] - P[y_1^b > y_0^a]\right|, \tag{22}$$

Interpretation: A positive ΔxAUROC indicates that group b is disadvantaged compared to group a in terms of xAUROC, while a negative value reflects the opposite. This metric is anti-symmetric, meaning that $\Delta\text{xAUROC}(a, b) = -\Delta\text{xAUROC}(b, a)$. It captures the magnitude of ranking disparities.

3.1.1.5 Subgroup AUROC (sAUROC)

The subgroup AUROC (sAUROC) evaluates subgroup-specific false positive rates (FPR) while computing the true positive rate (TPR) over the entire population. (Meissen *et al.*, 2024)

Mathematical Definition:

$$\text{sAUROC}(s) = \int_0^1 \text{TPR}_{\mathcal{P}}(\text{FPR}_s)d(\text{FPR}_s), \tag{23}$$

$\text{TPR}_{\mathcal{P}}$ is the whole population's TPR and FPR_s the subgroup's FPR.

Interpretation: The sAUROC captures performance differences between subgroups by isolating subgroup-specific false positive rates while leveraging population-wide true positive rates. A higher sAUROC suggests better alignment between subgroup performance and overall population performance, while disparities are highlighted by deviations in sAUROC values across subgroups.

3.1.1.6 Pairwise Ranking Fairness (PRF)

Pairwise Ranking Fairness (PRF) quantifies the probability that a randomly selected positive instance from a group a is ranked higher than a randomly selected negative instance from either group a or any other group b. (Cui *et al.*, 2023)

Mathematical Definition:

$$\text{PRF}(a) = P[y_1^a > y_0] = \frac{1}{n_1^a \cdot n_0} \sum_{i:i\in a, Y_i=1} \sum_{j:Y_j=0} \mathbb{I}[y(X_i, a) > y(X_j)], \tag{24}$$

Interpretation: PRF evaluates ranking fairness for positive instances of group a, measuring their likelihood of being ranked higher than negative instances from any group.

3.1.1.7 PRF Disparity (ΔPRF)

The PRF Disparity (ΔPRF) measures the absolute difference in pairwise ranking fairness between two groups a and b. It captures disparities in ranking probabilities across groups. (Cui *et al.*, 2023)

Mathematical Definition:

$$\Delta\text{PRF}(a, b) = \left|P[y_1^a > y_0] - P[y_1^b > y_0]\right|. \tag{25}$$

Interpretation: A ΔPRF value of 0 indicates that the ranking fairness for positive instances is consistent between groups a and b. Larger values highlight disparities in ranking performance between groups.

3.1.1.8 Equity-Scaled Recall (ES-Recall)

Equity-scaled metrics (ES-M) are a family of metrics for incorporating both a fairness notion (of equity) and performance into the evaluation of models. ES-M can be applied to any conventional metric $\mathcal{M}$, such as Accuracy or AUROC, to evaluate both efficiency model performance and consistency. This metric adjusts the overall performance metric $\mathcal{M}$ by accounting for performance disparities across identity groups. (Luo *et al.*, 2024)

Mathematical Definition:

$$\text{ES-}\mathcal{M} = \frac{\mathcal{M}(X,Y)}{1+\Delta}, \tag{26}$$

where $\mathcal{M}(X,Y)$ represents the conventional metric calculated on the entire dataset, and Δ captures the performance disparity across groups, defined as:

$$\Delta = \sum_{i\in\mathcal{A}} \left|\mathcal{M}(X,Y) - \mathcal{M}(X,Y \mid A=i)\right|, \tag{27}$$

where $\mathcal{M}(X,Y \mid A)$ is the metric evaluated for the set of identity groups $\mathcal{A}$ defined by the sensitive attribute A.

Interpretation: The ES-M metric balances ”equity” and performance by penalising large performance disparities (Δ). Key properties include:

- When $\Delta \to 0$ (perfect ”equity”), ES-$\mathcal{M} \to \mathcal{M}$, meaning the equity-scaled metric equals the original performance metric.
- As Δ increases (greater disparity), ES-$\mathcal{M}$ decreases, reflecting reduced equity.

Bounds for ES-M are constrained as:

$$\frac{\mathcal{M}(X,Y)}{1+\max(\Delta)} \leq \text{ES-}\mathcal{M} \leq \mathcal{M}(X,Y), \tag{28}$$

ensuring ES-M is always less than or equal to the original metric $\mathcal{M}$.

Example: If $\mathcal{M}$ = Recall, then ES-Recall is given by:

$$\text{ES-Recall} = \frac{\text{Overall Recall}}{1+\Delta}, \tag{29}$$

where $\Delta = \sum_i |\text{Overall Recall} - \text{Group Recall}_i|$.

Implications: This formulation penalises disparities in metric M across groups, ensuring a model is both valid with respect for that metric M and equitable according to the formulation above. The added value of this metric is unclear, as it should still be accompanied by the original $\mathcal{M}$ metric to assess performance variation after equity-scaling.

3.1.1.9 Mean Performance-Scaled Disparity (PSD) for AUROC

Mean-PSD quantifies the disparity in AUROC performance across subgroups, scaled by the overall AUROC of the model. It is calculated as the standard deviation of subgroup AUROCs normalised by the overall AUROC. (Luo *et al.*, 2023)

Mathematical Definition:

$$\text{Mean-PSD} = \sqrt{\frac{\sum_{a\in\mathcal{A}}(\text{AUROC}_a - \text{AUROC}_{\text{mean}})^2}{N_{\mathcal{A}}}} \Big/ AUROC_{Overall}, \tag{30}$$

where $N_{\mathcal{A}}$ is the total number of subgroups of A.

Interpretation: A smaller Mean-PSD value indicates less variability in subgroup AUROC, reflecting more equitable performance across groups. Larger values suggest greater disparities in AUROC performance across subgroups.

3.1.1.10 Maximum Performance-Scaled Disparity (PSD) for AUROC

Max-PSD evaluates the maximum disparity in AUROC performance between subgroups, scaled by the overall AUROC. It compares the largest and smallest subgroup AUROC relative to the overall model performance. (Luo *et al.*, 2023)

Mathematical Definition:

$$\text{Max-PSD} = \frac{\text{AUROC}_{a_{\max}} - \text{AUROC}_{a_{\min}}}{AUROC_{Overall}}, \tag{31}$$

Interpretation: A smaller Max-PSD value indicates that the disparity between the best- and worst-performing subgroups is minimal, reflecting fairer model performance across groups. Higher values highlight significant performance differences.

3.1.1.11 Concordance Imparity (CI)

Concordance Imparity (CI) quantifies disparities in the discriminative ability of a model across different groups, particularly in the presence of censorship. It evaluates fairness by measuring the maximum deviation between the weighted subgroup-based concordance fractions $CF(a)$ for all pairs of patients a and a' within a sensitive attribute A. (W. Zhang and Weiss, 2022)

Mathematical Definition:

$$CI = \max_{a,a' \in A, a \neq a'} \left| CF(a) - CF(a') \right|, \tag{32}$$

where the concordance fraction for a group a, $CF(a)$, is defined as:

$$CF(A = a) = \frac{C_a}{P_a} = sAUC_a, \tag{33}$$

and:

- C_a: Number of concordant pairs of patients in group a,
- P_a: Total number of permissible pairs of patients in group a,

The ratio defined above can be interpreted as a variation of AUROC and so, alternatively, CI can be computed as the largest deviation among weighted sAUCs, as shown below:

$$CI = \max_{a,a' \in A \text{ and } a \neq a'} \left| sAUC_{(A=a)} - sAUC_{(A=a')} \right|, \tag{34}$$

where the weighted subgroup-based AUC, $sAUC_{(A=a)}$, is calculated as:

$$sAUC_{(A=a)} = \frac{n_a sAUC_a + \sum_{j \neq a} n_{a'} sAUC_{a'}}{\sum_{j=1}^{|A|} n_j}, \tag{35}$$

and:

- n_a: Number of samples in subgroup a,
- $sAUC_a$: Subgroup AUC for intra-group observations in a,
- $sAUC_{a'}$: Subgroup AUC for inter-group observations with a'.

Interpretation: CI measures the largest disparity in subgroup concordance scores across all groups, with lower values indicating more consistent model performance (in terms of concordance) across subgroups.

3.1.1.12 AUROC Parity Ratio

The AUROC Parity Ratio measures the ratio of AUROC values between two subgroups defined by the sensitive attribute A. (Byrd *et al.*, 2023)

Mathematical Definition:

$$\mathcal{R}_{\text{AUROC}} = \frac{\text{AUROC}_{A=1}}{\text{AUROC}_{A\neq 1}}. \tag{36}$$

Interpretation: A ratio of 1 indicates no differences in AUROC across groups. Values above or below 1 indicate that one group has higher or lower AUROC than the other.

Implications: Minimising deviation from 1 means aiming for similar discrimination across groups. However, natural differences in underlying group characteristics can influence AUROC values and should be accounted for when interpreting results.

3.1.2 Calibration

3.1.2.1 Expected Calibration Error (ECE) Parity

Expected Calibration Error (ECE) measures how closely the estimated probabilities $\hat{p}$ of a model align with the actual probabilities y. ECE parity quantifies the difference in calibration between the groups defined by the sensitive attribute A. (Anderson *et al.*, 2024; Y. Yang *et al.*, 2024)

Mathematical Definition:

$$\mathcal{D}_{\text{ECE}} = \left|\text{ECE}_{A=1} - \text{ECE}_{A\neq 1}\right|, \tag{37}$$

Interpretation: ECE parity is 0 when the model is equally calibrated across groups $A = 1$ and $A \neq 1$.

3.1.2.2 Calibration-in-the-large Parity

The Calibration-in-the-large Parity measures the difference in calibration-in-the-large between two subgroups defined by the sensitive attribute A. It quantifies whether systematic overestimation or underestimation of risk varies across groups.(F. Chen *et al.*, 2024; Röösli *et al.*, 2022)

Mathematical Definition:

$$\mathcal{D}_{\text{Calibration-in-the-large}} = \left|\text{Calibration-in-the-large}_{A=1} - \text{Calibration-in-the-large}_{A\neq 1}\right|, \tag{38}$$

where calibration-in-the-large for a group $A = a$ is defined as:

$$\text{Calibration-in-the-large}_{A=a} = \frac{1}{N_a}\sum_{i\in A=a}\hat{p}_i - \frac{1}{N_a}\sum_{i\in A=a} y_i, \tag{39}$$

and N_a being the total number of instances in group $A = a$, $\hat{p}_i$ the predicted risk, and y_i the actual risk.

Interpretation: A gap of 0 indicates that calibration-in-the-large is consistent across subgroups, implying no systematic bias between the groups. Non-zero values reflect differences in over- or underestimation of risk between the groups.

3.1.2.3 Absolute Calibration Error Parity

The Absolute Calibration Error (ACE) Parity measures the difference in ACE between two subgroups defined by the sensitive attribute A. This metric quantifies disparities in overall calibration between groups by assessing deviations from perfect calibration. (S. Pfohl *et al.*, 2022)

Mathematical Definition:

$$\mathcal{D}_{\text{ACE}} = \left|\text{ACE}_{A=1} - \text{ACE}_{A\neq 1}\right|, \tag{40}$$

where ACE for a group $A = a$ is defined as:

$$\text{ACE}_{A=a} = \mathbb{E}_{A=a}\left[\left(g_\phi(\hat{p}) - \hat{p}\right)^2\right], \tag{41}$$

and g_ϕ is an auxiliary estimator approximating $P(Y = 1 \mid y)$.

Interpretation: A parity value of 0 indicates consistent absolute calibration between subgroups, while non-zero values highlight disparities in how well the estimated probabilities align with observed outcomes across groups.

3.1.2.4 Equal Calibration

Also known as Test-fairness or Matching Conditional Frequencies. This metric requires that, for any estimated probability value, both groups have similar positivity rates in the ground-truth. (Chouldechova, 2017)

Mathematical Definition:

$$\mathcal{D}_{\text{calibration}} = \left| P[Y = 1|A = 1, \hat{p}] - P[Y = 1|A \neq 1, \hat{p}] \right| \tag{42}$$

where $\hat{p}$ represents the estimated probability value. Conditioning on $\hat{p}$ means that few data points will be available for each conditional probability computation, which may become impractical. It is also not clear how to combine the several $\hat{p}$ upon which conditioning is to be done.

Interpretation: Similar conditional frequencies across groups, as defined above, for each estimated probability value suggests equal calibration.

Implications: Minimising this metric will result in similar calibration across groups, which may imply fairness from a calibration perspective. Although desirable in most cases, equal calibration is incompatible with other fairness metrics, such as equalised odds (Kleinberg *et al.*, 2016). The implementation of this metric is not sufficiently clear.

3.1.2.5 Well Calibration

Well calibration is an extension of "Equal calibration" 3.1.2.4, requiring that the actual probability of being in the positive class not only matches the estimated probability across all groups, but also a target probability. (Caton and Haas, 2024; Kleinberg *et al.*, 2016)

Mathematical Definition:

$$P(Y = 1 \mid A = 1, \hat{p}) = P(Y = 1 \mid A \neq 1, \hat{p}) = s_\epsilon \tag{43}$$

This "well calibration" can be operationalised as the difference between each group's estimated probability and the target probability s_ϵ.

Interpretation: The higher the difference between each group's estimated probability and the target threshold, the less fair and performant the model is.

Implications: Achieving well calibration ensures that estimated probabilities align with actual probabilities for all groups, reducing disparities in risk estimation. This may be necessary in scenarios where clinical or operational guidelines define specific risk thresholds to ensure compliance, such as the risk of atherosclerotic cardiovascular disease (ASCVD) to inform statin initiation. (S. Pfohl *et al.*, 2022)

3.1.3 Overall

3.1.3.1 Brier Score Parity

The Brier Score Parity measures the difference in Brier Score between two subgroups defined by the sensitive attribute A. (S. Pfohl *et al.*, 2019)

Mathematical Definition:

$$\mathcal{D}_{\text{Brier}} = \left| \text{Brier}_{A=1} - \text{Brier}_{A\neq1} \right|, \tag{44}$$

Interpretation: A parity value of 0 indicates no differences in Brier Score across groups, implying consistent calibration and prediction accuracy between subgroups.

3.1.3.2 Log-Loss Parity

The Log-Loss Parity measures the difference in logarithmic loss (or binary cross-entropy) between two subgroups defined by the sensitive attribute A. (S. Pfohl *et al.*, 2022; R. Wang *et al.*, 2024)

Mathematical Definition:

$$\mathcal{D}_{\text{Log Loss}} = \left|\text{LogLoss}_{A=1} - \text{LogLoss}_{A\neq 1}\right|, \tag{45}$$

where log loss for a group $A = a$ with N_a instances is defined as:

$$\text{LogLoss}_{A=a} = -\frac{1}{N_a} \sum_{i \in A=a} \left[Y_i \log(\hat{p}_i) + (1 - Y_i)\log(1 - \hat{p}_i)\right], \tag{46}$$

Interpretation: A parity value of 0 indicates that log loss is consistent across subgroups, suggesting that the model performs equally well in terms of probabilistic predictions for both groups. Non-zero values reflect disparities in prediction error between the groups.

3.1.3.3 Earth Mover's Distance (EMD) for equivalent separation

The EMD, also known as the first Wasserstein distance, can quantify the distance between the empirical distributions of estimated probabilities ($\hat{p}$) conditioned on the sensitive attribute (A) and the true outcome (Y). This metric enforces a separation criterion across all possible thresholds, requiring the distributions of estimated probabilities for each sensitive group to be as similar as possible within each outcome stratum. (S. Pfohl *et al.*, 2019)

Mathematical Definition:

$$EMD_{subgroups} = \frac{1}{|Y|} \sum_{Y_i \in Y} \frac{1}{|\mathcal{A}|^2} \sum_{a_i, a_j \in \mathcal{A}} W_1 \left(P(\hat{Y} \mid A = a_i, Y = Y_i), P(\hat{Y} \mid A = a_j, Y = Y_i)\right), \tag{47}$$

where:

- W_1: First Wasserstein distance (Earth Mover's Distance),
- $p(\hat{Y} \mid A = a, = Y_i)$: Empirical distribution of estimated probabilities ($\hat{Y}$) conditioned on the sensitive group $A = a$ and outcome $Y = y$,
- $\mathcal{A}$: Set of sensitive groups,
- $|Y|$: Number of possible outcome classes.

Interpretation: An EMD value of 0 indicates that the estimated probability distributions are identical across sensitive groups within each outcome stratum. Higher values reflect greater divergence in distributions, highlighting disparities in probabilistic estimations across groups.

Implications: The EMD metric is stricter than Equalised Odds, as it enforces separation across all thresholds. This implies that ROC curves for the groups must also be as similar as possible.

3.1.3.4 Balance for Positive Class

This criterion requires that subjects from both protected and unprotected groups in the positive class have equal average estimated probabilities, ensuring that one group does not consistently receive higher probabilities than the other. (Kleinberg *et al.*, 2016)

Mathematical Definition:

$$\mathcal{D}_{\text{balance positive}} = \mathbb{E}(\hat{p} \mid Y = 1, A = 1) - \mathbb{E}(\hat{p} \mid Y = 1, A \neq 1) \tag{48}$$

Interpretation: Balance for the positive class implies that, on average, individuals in the positive class receive comparable probabilities across groups.

Implications: In a clinical setting, this criterion ensures consistency in estimated probabilities for patients likely to have a positive outcome.

3.1.3.5 Balance for Negative Class

Similar to balance for the positive class, this definition requires that subjects in the negative class from both protected and unprotected groups receive equal average estimated probabilities (Kleinberg *et al.*, 2016).

Mathematical Definition:

$$\mathcal{D}_{\text{balance negative}} = \mathbb{E}(\hat{p} \mid Y = 0, A = 1) - \mathbb{E}(\hat{p} \mid Y = 0, A \neq 1) \quad (49)$$

Interpretation: Balance for the negative class implies that individuals in the negative class should have similar estimated probabilities on average across groups.

Implications: For clinical predictions, this criterion reduces the risk of disproportionate error in estimated probabilities for patients likely to have a negative outcome.

3.2 Threshold-dependent

3.2.1 Partial

3.2.1.1 Equal Opportunity Difference

Also known as Recall Parity (or Gap) or False Negative Error Rate Balance. This measure, introduced by (Hardt *et al.*, 2016), requires that TPRs are similar across groups.

Mathematical Definition:

$$\mathcal{D}_{\text{TPR}} = \left| P[\hat{Y} = 1 | A = 1, Y = 1] - P[\hat{Y} = 1 | A \neq 1, Y = 1] \right| = \mathcal{D}_{\text{FNR}} \quad (50)$$

Interpretation: This metric focuses only on TPRs, ensuring that individuals with a positive outcome are equally likely to receive a positive prediction.

Implications: It is a descriptive metric as it can measure disparities in decision making related, for example, to access to health care (Cheng *et al.*, 2021; M. Y. Yang *et al.*, 2023). Ensuring equal opportunity in one error type (e.g., TPR) can lead to increased disparities in other error types, such as false negatives. Furthermore, this metric can be minimised if one aims for the recall of both groups to be equal (for example selecting different threshold per group such that the recall is the same for both groups), which can result in a deterioration of other important metrics (Mittelstadt *et al.*, 2023).

3.2.1.2 Predictive Equality.

Also known as False Positive Error Rate Balance or Specificity Parity. This metric requires that FPRs are similar across groups, meaning that individuals with a negative outcome are equally likely to receive a positive prediction across groups.(Pessach and Shmueli, 2022)

Mathematical Definition:

$$\mathcal{D}_{\text{FPR}} = \left| P[\hat{Y} = 1 | A = 1, Y = 0] - P[\hat{Y} = 1 | A \neq 1, Y = 0] \right| = \mathcal{D}_{\text{TNR}} \quad (51)$$

Interpretation: A lower value indicates that FPRs are similar across groups, reducing disparities in false positives.

Implications: While highly relevant in healthcare contexts where false positives can induce harm to the patient (Cheng *et al.*, 2021; M. Y. Yang *et al.*, 2023), this measure only considers one type of error, which can make it insufficient for full fairness assessment. Ensuring equality in one error type can increase disparities in other types of errors, as discussed in prior metrics.

3.2.1.3 Equalised Odds

Also known as conditional procedure accuracy equality or disparity mistreatment. Initially proposed by (Hardt *et al.*, 2016), equalised odds aims to overcome the limitations of demographic parity and related measures. It combines both Equal Opportunity (TPRs) and Predictive Equality (FPRs).

Mathematical Definition:

$$\begin{aligned}\mathcal{D}_{\mathrm{FPR}} &= \left|P[\hat{Y}=1 \mid A=1, Y=0] - P[\hat{Y}=1 \mid A\neq 1, Y=0]\right| \\ &= \left|FPR_{A=1} - FPR_{A\neq 1}\right| \\ &\wedge \\ \mathcal{D}_{\mathrm{TPR}} &= \left|P[\hat{Y}=1 \mid A=1, Y=1] - P[\hat{Y}=1 \mid A\neq 1, Y=1]\right| \\ &= \left|TPR_{A=1} - TPR_{A\neq 1}\right| \end{aligned} \tag{52}$$

Operationalising this into a single measure may mean taking the mean, maximum, or use some other aggregation strategy to combine $\mathcal{D}_{\mathrm{TPR}}$ and $\mathcal{D}_{\mathrm{FPR}}$.

Interpretation: Equalised odds compares both FPRs and TPRs across groups, providing a more complete view of this kind of fairness.

Implications: A fully accurate classifier will satisfy equalised odds, assuming unbiased base rates in each group. Equalised odds further enforces that the accuracy is equally high across all groups, punishing models that only perform well on majority groups. This measure depends on the true outcome (ground-truth), requiring that base rates are representative and not biased. When implementing equalised odds, to ensure a single scalar value is returned, the maximum or an average of TPR and FPR differences across groups can be reported, and this design choice should be clearly reported.

3.2.1.4 Equalising Disincentives

This metric compares the difference between TPR and FPR across protected groups. (Caton and Haas, 2024; Jung *et al.*, 2020; Wan *et al.*, 2023)

Mathematical Definition:

$$\mathcal{D}_{\text{equalising disincentives}} = (\mathrm{TPR}_{A=1} - \mathrm{FPR}_{A=1}) - (\mathrm{TPR}_{A\neq 1} - \mathrm{FPR}_{A\neq 1}), \tag{53}$$

Interpretation: A value of 0 indicates that the difference between TPR and FPR is equal across groups, signalling balanced incentives for correct and incorrect classifications of positives. This could be interpreted as the difference across groups of a special case (threshold 0.5) of net benefit.

Implications: It seems to be utility-inspired.

3.2.1.5 Average Odds Difference

This metric computes the average difference between the FPR and TPR across protected groups. It combines the concepts of equalised odds (conjunction of equality of opportunity difference and predictive equality) into a single measure (Alam, 2020; J. Zhang *et al.*, 2024)

Mathematical Definition:

$$\mathcal{D}_{\text{average odds}} = \frac{1}{2}\left(\mathrm{FPR}_{A=1} - \mathrm{FPR}_{A\neq 1} + \mathrm{TPR}_{A=1} - \mathrm{TPR}_{A\neq 1}\right), \tag{54}$$

Interpretation: A value of 0 indicates equalised odds between groups. Values less than 0 imply a higher benefit for the privileged group, while values greater than 0 indicate a higher benefit for the unprivileged group.

3.2.1.6 Average Disparity in Equalised Odds

This metric measures the average deviation from the ideal Equalised Odds (EO) condition by evaluating the pairwise differences in TPR and FPR across all subgroups defined by a sensitive attribute A. It aggregates these differences into a single measure, capturing subgroup-specific disparities. (Y. Wang *et al.*, 2024)

Mathematical Definition:

$$\mathrm{EO}_{\mathrm{TPR}} = \frac{1}{\binom{|A|}{2}} \sum_{a_i} \sum_{a_j > a_i} \left|\mathrm{TPR}_{a_i} - \mathrm{TPR}_{a_j}\right|, \tag{55}$$

$$\mathrm{EO}_{\mathrm{FPR}} = \frac{1}{\binom{|A|}{2}} \sum_{a_i} \sum_{a_j > a_i} \left| \mathrm{FPR}_{a_i} - \mathrm{FPR}_{a_j} \right| \tag{56}$$

Here, A is the set of subgroups defined by A, and $\binom{|A|}{2}$ is the number of unique subgroup pairs. Similarly to equalised odds 3.2.1.3, both partial measures must be combined into a single measure, using an average or a maximum operator, for example.

Interpretation: This metric quantifies fairness disparities across all subgroups. A lower value indicates smaller deviations in TPR and FPR across subgroups, moving closer to achieving Equalised Odds. It is particularly useful in settings with diverse or unevenly represented subgroups defined by A.

3.2.1.7 Predictive Parity

Also known as outcome test. This metric requires that the positive predictive values (PPVs) are similar across groups (DiCiccio *et al.*, 2023). This is equivalent to precision.

Mathematical Definition:

$$\mathcal{D}_{\mathrm{PPV}} = \left| P[Y = 1 | A = 1, \hat{Y} = 1] - P[Y = 1 | A \neq 1, \hat{Y} = 1] \right| = \mathcal{D}_{\mathrm{FDR}} \tag{57}$$

Interpretation: Lower values of this metric indicate that the PPVs across groups are similar, suggesting fairer outcomes for individuals receiving a positive prediction.

Implications: Meeting this metric would mean that the probability of an individual with a positive prediction actually experiencing a positive outcome is the same across groups. As it relies on the ground-truth outcome, this metric assumes outcomes were achieved fairly. It can be incompatible with equalised odds or equal opportunity if prevalence differs across groups (Barocas *et al.*, 2023).

3.2.1.8 NPV Difference

This metric computes the difference in NPV between protected groups. It measures disparities in the accuracy of negative predictions across groups, ensuring that $P(Y = 0 \mid \hat{Y} = 0)$ is consistent with respect to sensitive attributes. (Jaime and Kern, 2024)

Mathematical Definition:

$$\mathcal{D}_{\mathrm{NPV}} = \left| \mathrm{NPV}_{A=1} - \mathrm{NPV}_{A \neq 1} \right| = \mathcal{D}_{\mathrm{FOR}}, \tag{58}$$

Interpretation: A value of 0 indicates parity in NPV between groups.

Implications: Potentially relevant when needed to ensure that negative predictions are equally performant across groups.

3.2.1.9 Conditional Equality of Opportunity Gap (EOGap)

The Conditional Equality of Opportunity Gap (EOGap) quantifies disparities in opportunity gaps across subgroups, conditioned on L, a covariate subspace of X that represents variables that legitimately modify risk across subgroups, and weighted by a probability p_a for each value $a \in \mathcal{A}$, where A represents the sensitive attribute. (Beutel *et al.*, 2019)

Mathematical Definition:

$$\mathrm{EOGap} = \sum_{l \in \mathcal{L}} p_l \left[P(\hat{Y} = 1 \mid Y = 1, A = 1, L = l) - P(\hat{Y} = 1 \mid Y = 0, A \neq 1, L = l) \right], \tag{59}$$

where p_l is the probability weight assigned to each value $l \in \mathcal{L}$, a set of features to condition upon.

Interpretation: Setting $p_l = \frac{1}{|\mathcal{L}|}$ equally weights all subgroup values. Alternatively, $p_l = P(L = l \mid S = 0)$ can be used to align with the background distribution of the sensitive attribute, ensuring subgroup weighting reflects the overall data distribution.

Implications: The set of features to condition upon still needs to be subjectively selected.

3.2.1.10 Conditional Use Accuracy Equality.

Similar to Equalised Odds, but related to PPV and NPV. Also known as positive and negative predictive parity. Requires equal positive and negative predictive values across groups. (Pessach and Shmueli, 2022; Verma and Rubin, 2018)

Mathematical Definition:

$$\begin{aligned} \mathcal{D}_{\text{PPV}} &= P(Y=1 \mid \hat{Y}=1, A=1) - P(Y=1 \mid \hat{Y}=1, A \neq 1) \\ &\wedge \\ \mathcal{D}_{\text{NPV}} &= P(Y=0 \mid \hat{Y}=0, A=1) - P(Y=0 \mid \hat{Y}=0, A \neq 1) \end{aligned} \tag{60}$$

Similarly to equalised odds 3.2.1.3, these two partial measures should then be combined into a single metric (unclear how). It is also unclear how to calculate probabilities that are simultaneously conditioned upon Y, A, and L.

Interpretation: This measure implies that individuals with similar predicted outcomes (positive or negative) should have similar true outcomes across groups.

3.2.1.11 Recall Equality Difference

The Recall Equality Difference measures the average absolute difference in recall (TPR) between each demographic group and the overall recall across all groups. This metric quantifies disparities in recall across groups within a given dimension. (Xiao *et al.*, 2023) This metric is an extension of the Equal Opportunity Difference 3.2.1.1 to multiple groups defined by a sensitive attribute A.

Mathematical Definition:

$$\text{Recall Equality Difference} = \frac{1}{|\mathcal{A}|} \sum_{i \in \mathcal{A}} |TPR - TPR_{A=i}| \,, \tag{61}$$

Interpretation: A value of 0 indicates perfect recall equality across all groups, while larger values reflect greater disparities in recall between groups.

3.2.1.12 Recall Disparity Ratio

The Recall Disparity Ratio measures the ratio of recall (TPR) between the group with the highest recall and the group with the lowest recall. It quantifies the relative performance gap between the best- and worst-performing groups. This could be applied to any metric other than TPR. (Riad *et al.*, 2024)

Mathematical Definition:

$$\text{Disparity Ratio} = \frac{\min_{i \in \mathcal{A}} TPR_{A=i}}{\max_{j \in \mathcal{A}} TPR_{A=j}}, \tag{62}$$

Interpretation: A Disparity Ratio of 1 indicates equal recall across all groups, while smaller values highlight larger performance gaps.

3.2.1.13 Worst-Group Outcome

The Worst-Group Outcome focuses on the minimum recall (TPR) across all demographic groups. It identifies the lowest-performing group and ensures that the fairness evaluation prioritizes improvements for the most disadvantaged group. (Riad *et al.*, 2024; Diana *et al.*, 2021; Tarek *et al.*, 2024; Xia *et al.*, 2024)

Mathematical Definition:

$$\text{Worst-Group Outcome} = \min_{i \in \mathcal{A}} TPR_{A=i}. \tag{63}$$

Interpretation: Higher values indicate better performance for the worst-performing group. This metric aligns with minimax fairness, aiming to improve outcomes for the most disadvantaged group.

Implications: This metric seems to be related to John Rawls' definition of fairness, aiming to maximise the minimum benefit across individuals.

3.2.1.14 Maximum Difference Recall

The Maximum Difference Recall measures the largest absolute difference between the recall (TPR) of a specific demographic group and the overall recall across all groups. This metric identifies the group experiencing the largest deviation from the average recall. It can be generalised to other base classification metrics. (Xiao *et al.*, 2023)

Mathematical Definition:

$$\text{Maximum Difference Recall} = \max_{i \in \mathcal{A}} |TPR_{A=i} - TPR|, \tag{64}$$

Interpretation: A value of 0 indicates perfect alignment of all groups' recall with the overall recall, while larger values reflect the magnitude of the largest deviation experienced by any group.

3.2.1.15 Recall Intergroup Standard Deviation (Recall-ISD)

The Intergroup Standard Deviation (ISD) family of metrics measures the variability of a chosen metric M across multiple demographic groups defined by a sensitive attribute A. ISD evaluates disparities in the performance of M across groups, offering a generalisable metric for fairness analysis. (Y. Huang *et al.*, 2024; Foryciarz *et al.*, 2022)

Mathematical Definition:

$$\text{ISD}(\mathcal{M}, A) = \sqrt{\frac{1}{|A|} \sum_{i=1}^{|A|} (\mathcal{M}_i - \mathcal{M})^2}, \tag{65}$$

The ISD can be applied to various performance metrics, such as TPR and accuracy, among others. The Recall Intergroup Standard Deviation measures the variability of true positive rates (TPR) across groups (Poulain *et al.*, 2023):

$$\text{TPSD} = \sqrt{\frac{1}{|A|} \sum_{a=1}^{|A|} (TPR_{A=a} - TPR)^2},$$

Interpretation: An ISD value of 0 indicates perfect consistency of metric M across all groups, while larger values reflect greater disparities in M.

3.2.1.16 Recall Coefficient of Variation (Recall-CV)

The Coefficient of Variation (CV) measures the relative variability of a chosen metric M across multiple demographic groups defined by a sensitive attribute A. It quantifies the ratio of the standard deviation of the metric to its mean, providing a normalised measure of variability that is independent of the scale of the metric. (S. Pfohl *et al.*, 2019)

Mathematical Definition:

$$\mathcal{M}\text{-CV}(\mathcal{M}, A) = \frac{\sqrt{\frac{1}{|A|} \sum_{i=1}^{|A|} (\mathcal{M}_i - \mathcal{M})^2}}{\mathcal{M}}, \tag{66}$$

The CV metric can be applied to various performance metrics, such as TPR, AUROC, or ECE. The Recall (TPR) Coefficient of Variation (Recall-CV) measures the relative variability of TPR across groups:

$$\text{Recall-CV} = \frac{\sqrt{\frac{1}{|A|} \sum_{a=1}^{|A|} (TPR_{A=a} - TPR)^2}}{TPR} \tag{67}$$

Interpretation: A CV value of 0 indicates no relative variability in the metric M across groups, while higher values reflect greater disparities normalised to the scale of M.

Implications: This metric can potentially be useful for comparing variability across metrics that may differ in scale, offering consistency.

3.2.1.17 Between-group Generalised Entropy Index (GEI)

The Generalised Entropy Index (GEI) measures the inequality in outcomes of an algorithm at both the individual and group levels. (Speicher *et al.*, 2018; Y. Huang *et al.*, 2024; H. E. Wang *et al.*, 2024)

Mathematical Definition:

$$\mathcal{E}^{\alpha}(b_1, b_2, \ldots, b_n) = \frac{1}{n\alpha(\alpha-1)} \sum_{i=1}^{n} \left[\left(\frac{b_i}{\mu} \right)^{\alpha} - 1 \right], \tag{68}$$

where:

- b_i: Benefit function for individual i, representing the predictive bias or advantage conferred,
- μ: Mean benefit across all individuals, $\mu = \frac{1}{n}\sum_{i=1}^{n} b_i$,
- α: Weight parameter controlling sensitivity to disparities ($\alpha \notin \{0, 1\}$),
- n: Number of individuals in the dataset.

When $\alpha = 0$, the GEI is the mean log deviation or Theil L index; when $\alpha = 1$ the GEI becomes the Theil T index, and when $\alpha = 2$ the GEI is equivalent half the squared coefficient of variation.

This overall notion, which can also be seen as a way of individual fairness (as it does not require defining sensitive attributes *a priori*), can be decomposed into two parcels: between-group and within-group (un)fairness. The former will be the focus here. It can be computed by separating the patients into subsets according to the sensitive attribute being studied. Each subgroup is then assigned a benefit that is the average of the benefit of all the individuals in the group. Then, the average benefits of subgroups are fed into GEI and that is our metric.

A recall between-group GEI would have been implemented as described above with a suitable benefit function that incorporates a fairness notion of equal opportunity.

Interpretation: GEI and its parcels can be interpreted as a measure of information theoretic redundancy in data. It quantifies overall inequality in benefits across individuals and groups. Lower between-group-GEI values indicate greater equality in benefits, while higher values reflect increased disparity. By varying α, the GEI can emphasise different aspects of inequality. For large α the index is especially sensitive to the existence of large benefits, whereas for small α the index is especially sensitive to the existence of small benefits.

Implications: Utility-inspired but not explicitly proposed for healthcare or as a clinical utility metric.

3.2.1.18 Recall-HEAL

The Health Equity Assessment of machine Learning performance (HEAL) metric evaluates the anticorrelation between pre-existing health outcomes and AI tool performance across subpopulations for a given sensitive attribute. (Schaekermann *et al.*, 2024)

Mathematical Definition:

$$R = -\mathrm{corr}\left[(\mathrm{HO}_1, \mathrm{HO}_2, \ldots, \mathrm{HO}_N), (\mathrm{AI}_1, \mathrm{AI}_2, \ldots, \mathrm{AI}_N) \right], \tag{69}$$

where:

- corr: Spearman's rank correlation coefficient,
- HO_i: Quantitative measure of pre-existing health outcomes for subpopulation i,
- AI_i: AI tool performance for subpopulation i such as Recall in this case,
- N: Total number of subpopulations considered.

Interpretation: HEAL can be interpreted as a reparations-based metric that prioritises improved AI performance for historically disadvantaged subpopulations.The metric R is defined as the negated correlation coefficient, such that higher values of R (closer to 1.0) indicate that subpopulations with worse health outcomes receive higher AI performance, with the priority decreasing as health outcomes improve. In other words, the subpopulation with the worst outcomes achieves the highest AI performance, reflecting reparative fairness.

Implications: The HEAL metric highlights ethical considerations in AI fairness by focusing on reparations for historically disadvantaged subpopulations. This approach raises several debates, as it requires reliable and comparable measures of historical health outcomes and AI performance. Moreover, ensuring fairness for present and future implications is critical to avoid perpetuating new biases. Ethical scrutiny is necessary to balance reparative justice with broader equity goals.

3.2.2 Summary

3.2.2.1 Overall Accuracy Gap.

This metric requires that accuracy is similar across groups. (Caton and Haas, 2024; Verma and Rubin, 2018; M. Liu *et al.*, 2024)

Mathematical Definition:

$$\mathcal{D}_{\text{accuracy gap}} = \left|P[\hat{Y} = Y|A = 1] - P[\hat{Y} = Y|A \neq 1]\right| = \mathcal{D}_{\text{error disparity}} \tag{70}$$

This metric is mathematically equivalent to an error (or zero-one loss) disparity.

Interpretation: A smaller difference means that groups have similar prediction accuracy.

Implications: This metric does not assess the kind of mistakes done by the model, and is depedent on the selected threshold.

3.2.2.2 Error Rate Ratio

Also referred to as the zero-one loss ratio, this metric calculates the ratio of error rates between unprivileged and privileged groups. (I. Y. Chen *et al.*, 2019; J. Huang *et al.*, 2022) It is mathematically equivalent to the overall accuracy ratio.

Mathematical Definition:

$$\mathcal{D}_{\text{error rate}} = \frac{P[Y \neq \hat{Y} \mid A = 1]}{P[Y \neq \hat{Y} \mid A \neq 1]} = \mathcal{D}_{\text{accuracy rate}} \tag{71}$$

Interpretation: A ratio of 1 indicates parity in error rates between groups, while deviations from 1 highlight disparities in model performance.

Implications: Similar to accuracy parity.

3.2.2.3 Balanced Accuracy Difference

This metric evaluates the disparity in balanced accuracy (BACC) scores between a protected group and an unprotected group (Jaime and Kern, 2024).

Mathematical Definition:

$$\mathcal{D}_{\text{BACC}} = \text{BACC}(S = 1) - \text{BACC}(S \neq 1), \tag{72}$$

Interpretation: A difference closer to 0 indicates parity in performance across groups, according to balanced accuracy.

3.2.2.4 F1 Parity

This metric quantifies the variation in F1 score between groups defined by a sensitive attribute A. (Djebrouni *et al.*, 2024; D. Y. Zhang *et al.*, 2020)

Mathematical Definition:

$$\mathcal{D}_{\text{discrimination index}} = \text{F1}_{A=1} - \text{F1}_{A\neq 1}, \tag{73}$$

The original paper refers to this metric as "Discrimination Index" but such term seems disconnected from terminology found in the literature, as the F1-score does not refer to discrimination. We renamed this metric to F1 Parity.

Interpretation: A value of 0 indicates parity in F1 scores across groups.

Implications: F1-score has been regarded as an inadvisable metric for performance assessment as it ignores TN, conflates classification with clinical utility and has no intuitive interpretation. A difference in F1-score across subgroups has unclear implications.

3.2.2.5 Matthew's Correlation Coefficient (MCC) Parity

This metric evaluates whether the MCC is equal across groups. MCC is a measure of the relationship between actual and predicted values and is equivalent to the chi-square statistic for a two-by-two contingency table. (Anderson *et al.*, 2024; Chicco and Jurman, 2020)

Mathematical Definition:

$$\mathcal{D}_{\text{MCC}} = \text{MCC}_{A=1} - \text{MCC}_{A\neq 1}, \tag{74}$$

Interpretation: A value of 0 indicates parity in MCC across groups.

3.2.2.6 Treatment Equality

Also known as Error Rate Balance. This definition focuses on the ratio of FPs and FNs across groups. (Pessach and Shmueli, 2022)

Mathematical Definition:

$$\mathcal{D}_{\text{treatment equality}} = \frac{\text{FP}}{\text{FN}}_{A\neq 1} - \frac{\text{FP}}{\text{FN}}_{A=1} \tag{75}$$

Interpretation: Treatment Equality requires that the classifier has an equal proportion of errors across groups.

Implications: Seems to be utility-inspired but not explicitly defined as such.

3.2.2.7 Error Distribution Disparity Index (EDDI)

The EDDI measures the deviation of error rates across subgroups relative to the overall error rate (OER). It is designed to address fairness concerns in settings with significant data variability and diverse subgroup sizes, that are common in clinical applications. (Y. Wang *et al.*, 2024)

Mathematical Definition:

$$\text{EDDI} = \frac{1}{|A|} \sum_{a \in A} \frac{\text{ER}_a - \text{OER}}{\max(\text{OER}, 1 - \text{OER})}, \tag{76}$$

where:

$$\text{ER}_a = \frac{1}{N_a} \sum_{i \in a} \mathbb{I}(Y_i \neq \hat{Y}_i), \quad \text{OER} = \frac{1}{N} \sum_{i=1}^{N} \mathbb{I}(Y_i \neq \hat{Y}_i),$$

and:

- ER_a: Error rate for subgroup a,
- OER: Overall error rate across the dataset,
- N_a: Number of instances in subgroup a,

- N: Total number of instances in the dataset,
- $\mathbb{I}(Y_i \neq \hat{Y}_i)$: Indicator function, equal to 1 if $Y_i \neq \hat{Y}_i$, and 0 otherwise.

Interpretation: A lower EDDI value indicates reduced disparity in error rates across subgroups, signalling greater fairness. In general, consistent error rates across subgroups result in EDDI values closer to 0, while larger deviations increase EDDI, highlighting disparities.

3.2.3 Clinical Benefit

3.2.3.1 Subgroup Net Benefit

This metric evaluates the expected benefit of applying a predictive model to a subgroup, considering outcome prevalence, the proportion of true and false positives, and a weighting factor that reflects treatment effectiveness. (Benitez-Aurioles *et al.*, 2024)

Mathematical Definition:

$$sNB(\tau, \lambda) = 1 - \pi + \frac{\lambda}{N}\left(TP(\tau) - \frac{\tau}{1-\tau}FP(\tau)\right) \tag{77}$$

Where:

- π is the prevalence of the outcome,
- N is the subgroup size,
- $TP(\tau)$ and $FP(\tau)$ are the number of true positives and false positives at threshold τ,
- τ is the decision threshold,
- λ is the relative utility of treating a true positive compared to avoiding treatment of a true negative.

Interpretation: Higher values of $sNB(\tau, \lambda)$ indicate greater net clinical benefit from treating patients in a subgroup, assuming a given threshold τ and treatment utility λ.

Implications: This metric incorporates clinical relevance into model evaluation, but its value depends on accurately estimating outcome prevalence and the relative benefit of treatment.

References

Alam, Mohammad Arif Ul (2020). "Ai-fairness towards activity recognition of older adults", *MobiQuitous 2020-17th EAI International Conference on Mobile and Ubiquitous Systems: Computing, Networking and Services*, pp. 108–117.

Anderson, Joshua W, Shaikh, Nader, and Visweswaran, Shyam (2024). "Measuring and Reducing Racial Bias in a Pediatric Urinary Tract Infection Model", *AMIA Summits on Translational Science Proceedings*, Vol. 2024, p. 488.

Balagopalan, Aparna *et al.*, (2022). "The road to explainability is paved with bias: Measuring the fairness of explanations", *Proceedings of the 2022 ACM Conference on Fairness, Accountability, and Transparency*, pp. 1194–1206.

Barocas, Solon, Hardt, Moritz, and Narayanan, Arvind (2023). *Fairness and machine learning: Limitations and opportunities*, MIT press.

Benitez-Aurioles, Jose *et al.*, (2024). "Understanding algorithmic fairness for clinical prediction in terms of subgroup net benefit and health equity", *arXiv preprint arXiv:2412.07879*,

Beutel, Alex *et al.*, (2019). "Putting fairness principles into practice: Challenges, metrics, and improvements", *Proceedings of the 2019 AAAI/ACM Conference on AI, Ethics, and Society*, pp. 453–459.

Brier, Glenn W (1950). "Verification of forecasts expressed in terms of probability", *Monthly weather review*, Vol. 78 No. 1, pp. 1–3.

Byrd, Thomas F *et al.*, (2023). "Validation of a proprietary deterioration index model and performance in hospitalized adults", *JAMA Network Open*, Vol. 6 No. 7, e2324176–e2324176.

Caton, Simon and Haas, Christian (2024). "Fairness in machine learning: A survey", *ACM Computing Surveys*, Vol. 56 No. 7, pp. 1–38.

Chen, Feng *et al.*, (2024). "Unmasking bias in artificial intelligence: a systematic review of bias detection and mitigation strategies in electronic health record-based models", *Journal of the American Medical Informatics Association*, Vol. 31 No. 5, pp. 1172–1183.

Chen, Irene Y, Szolovits, Peter, and Ghassemi, Marzyeh (2019). "Can AI help reduce disparities in general medical and mental health care?", *AMA journal of ethics*, Vol. 21 No. 2, pp. 167–179.

Cheng, Victoria *et al.*, (2021). "Can you fake it until you make it? impacts of differentially private synthetic data on downstream classification fairness", *Proceedings of the 2021 ACM Conference on Fairness, Accountability, and Transparency*, pp. 149–160.

Chicco, Davide and Jurman, Giuseppe (2020). "The advantages of the Matthews correlation coefficient (MCC) over F1 score and accuracy in binary classification evaluation", *BMC genomics*, Vol. 21, pp. 1–13.

Chouldechova, Alexandra (2017). "Fair prediction with disparate impact: A study of bias in recidivism prediction instruments", *Big data*, Vol. 5 No. 2, pp. 153–163.

Corbett-Davies, Sam *et al.*, (2017). "Algorithmic decision making and the cost of fairness", *Proceedings of the 23rd acm sigkdd international conference on knowledge discovery and data mining*, pp. 797–806.

Coston, Amanda *et al.*, (2019). "Fair transfer learning with missing protected attributes", *Proceedings of the 2019 AAAI/ACM Conference on AI, Ethics, and Society*, pp. 91–98.

Cui, Sen *et al.*, (2023). "Bipartite ranking fairness through a model agnostic ordering adjustment", *IEEE Transactions on Pattern Analysis and Machine Intelligence*,

Diana, Emily *et al.*, (2021). "Minimax group fairness: Algorithms and experiments", *Proceedings of the 2021 AAAI/ACM Conference on AI, Ethics, and Society*, pp. 66–76.

DiCiccio, Cyrus *et al.*, (2023). "Detection and mitigation of algorithmic bias via predictive parity", *Proceedings of the 2023 ACM Conference on Fairness, Accountability, and Transparency*, pp. 1801–1816.

Djebrouni, Yasmine *et al.*, (2024). "Bias mitigation in federated learning for edge computing", *Proceedings of the ACM on Interactive, Mobile, Wearable and Ubiquitous Technologies*, Vol. 7 No. 4, pp. 1–35.

Feldman, Michael *et al.*, (2015). "Certifying and removing disparate impact", *proceedings of the 21th ACM SIGKDD international conference on knowledge discovery and data mining*, pp. 259–268.

Foryciarz, Agata *et al.*, (2022). "Evaluating algorithmic fairness in the presence of clinical guidelines: the case of atherosclerotic cardiovascular disease risk estimation", *BMJ Health & Care Informatics*, Vol. 29 No. 1.

Foulds, James R *et al.*, (2020). "An intersectional definition of fairness", *2020 IEEE 36th International Conference on Data Engineering (ICDE).* IEEE, pp. 1918–1921.

Guo, Chuan *et al.*, (2017). “On calibration of modern neural networks”, *International conference on machine learning.* PMLR, pp. 1321–1330.

Hanley, James A and McNeil, Barbara J (1982). “The meaning and use of the area under a receiver operating characteristic (ROC) curve.” *Radiology*, Vol. 143 No. 1, pp. 29–36.

Hardt, Moritz, Price, Eric, and Srebro, Nati (2016). “Equality of opportunity in supervised learning”, *Advances in neural information processing systems*, Vol. 29.

Hu, Shu and Chen, George H (2024). “Fairness in survival analysis with distributionally robust optimization”, *Journal of Machine Learning Research*, Vol. 25 No. 246, pp. 1–85.

Huang, Jonathan *et al.*, (2022). “Evaluation and mitigation of racial bias in clinical machine learning models: scoping review”, *JMIR Medical Informatics*, Vol. 10 No. 5, e36388.

Huang, Yu *et al.*, (2024). “A scoping review of fair machine learning techniques when using real-world data”, *Journal of Biomedical Informatics*, p. 104622.

Jaime, Sofia and Kern, Christoph (2024). “Ethnic classifications in algorithmic fairness: Concepts, measures and implications in practice”, *The 2024 ACM Conference on Fairness, Accountability, and Transparency*, pp. 237–253.

Jiang, Zhimeng *et al.*, (2022). “Generalized demographic parity for group fairness”, *International Conference on Learning Representations.*

Jung, Christopher *et al.*, (2020). “Fair prediction with endogenous behavior”, *Proceedings of the 21st ACM Conference on Economics and Computation*, pp. 677–678.

Kallus, Nathan and Zhou, Angela (2019). “The fairness of risk scores beyond classification: Bipartite ranking and the xauc metric”, *Advances in neural information processing systems*, Vol. 32.

Kearns, Michael *et al.*, (2018). “Preventing fairness gerrymandering: Auditing and learning for subgroup fairness”, *International conference on machine learning.* PMLR, pp. 2564–2572.

Keya, Kamrun Naher *et al.*, (2020). “Equitable allocation of healthcare resources with fair cox models”, *arXiv preprint arXiv:2010.06820*,

Kleinberg, Jon, Mullainathan, Sendhil, and Raghavan, Manish (2016). “Inherent trade-offs in the fair determination of risk scores”, *arXiv preprint arXiv:1609.05807*,

Liu, Mingxuan *et al.*, (2024). “Fairness-Aware Interpretable Modeling (FAIM) for Trustworthy Machine Learning in Healthcare”, *arXiv preprint arXiv:2403.05235*,

Luo, Yan *et al.*, (2023). “Eye fairness: A large-scale 3d imaging dataset for equitable eye diseases screening and fair identity scaling”, *arXiv preprint arXiv:2310.02492*,

Luo, Yan *et al.*, (2024). “Harvard glaucoma fairness: a retinal nerve disease dataset for fairness learning and fair identity normalization”, *IEEE Transactions on Medical Imaging*,

Meissen, Felix *et al.*, (2024). “(Predictable) performance bias in unsupervised anomaly detection”, *Ebiomedicine*, Vol. 101.

Mittelstadt, Brent, Wachter, Sandra, and Russell, Chris (2023). “The Unfairness of Fair Machine Learning: Levelling down and strict egalitarianism by default”, *arXiv preprint arXiv:2302.02404*,

Naeini, Mahdi Pakdaman, Cooper, Gregory, and Hauskrecht, Milos (2015). “Obtaining well calibrated probabilities using bayesian binning”, *Proceedings of the AAAI conference on artificial intelligence.* Vol. 29. No. 1.

Nguyen, Son, Wang, Adam, and Montillo, Albert (2023). “Fairness-enhancing mixed effects deep learning improves fairness on in-and out-of-distribution clustered (non-iid) data”, *arXiv preprint arXiv:2310.03146*,

Pessach, Dana and Shmueli, Erez (2022). “A review on fairness in machine learning”, *ACM Computing Surveys (CSUR)*, Vol. 55 No. 3, pp. 1–44.

Pfohl, Stephen *et al.*, (2019). “Creating fair models of atherosclerotic cardiovascular disease risk”, *Proceedings of the 2019 AAAI/ACM Conference on AI, Ethics, and Society*, pp. 271–278.

Pfohl, Stephen *et al.*, (2022). “Net benefit, calibration, threshold selection, and training objectives for algorithmic fairness in healthcare”, *Proceedings of the 2022 ACM Conference on Fairness, Accountability, and Transparency*, pp. 1039–1052.

Pfohl, Stephen R, Foryciarz, Agata, and Shah, Nigam H (2021). “An empirical characterization of fair machine learning for clinical risk prediction”, *Journal of biomedical informatics*, Vol. 113, p. 103621.

Poulain, Raphael, Bin Tarek, Mirza Farhan, and Beheshti, Rahmatollah (2023). “Improving fairness in ai models on electronic health records: The case for federated learning methods”, *Proceedings of the 2023 ACM conference on fairness, accountability, and transparency*, pp. 1599–1608.

Riad, Rachid *et al.*, (2024). “Automated speech analysis for risk detection of depression, anxiety, insomnia, and fatigue: Algorithm Development and Validation Study”, *Journal of Medical Internet Research*, Vol. 26, e58572.

Röösli, Eliane, Bozkurt, Selen, and Hernandez-Boussard, Tina (2022). "Peeking into a black box, the fairness and generalizability of a MIMIC-III benchmarking model", *Scientific Data*, Vol. 9 No. 1, p. 24.

Schaekermann, Mike *et al.*, (2024). "Health equity assessment of machine learning performance (HEAL): a framework and dermatology AI model case study", *EClinicalMedicine*, Vol. 70.

Speicher, Till *et al.*, (2018). "A unified approach to quantifying algorithmic unfairness: Measuring individual &group unfairness via inequality indices", *Proceedings of the 24th ACM SIGKDD international conference on knowledge discovery & data mining*, pp. 2239–2248.

Tarek, Mirza Farhan Bin, Poulain, Raphael, and Beheshti, Rahmatollah (2024). "Fairness-Optimized Synthetic EHR Generation for Arbitrary Downstream Predictive Tasks", *arXiv preprint arXiv:2406.02510*,

Verma, Sahil and Rubin, Julia (2018). "Fairness definitions explained", *Proceedings of the international workshop on software fairness*, pp. 1–7.

Wan, Mingyang *et al.*, (2023). "In-processing modeling techniques for machine learning fairness: A survey", *ACM Transactions on Knowledge Discovery from Data*, Vol. 17 No. 3, pp. 1–27.

Wang, H Echo *et al.*, (2024). "Assessing racial bias in healthcare predictive models: Practical lessons from an empirical evaluation of 30-day hospital readmission models", *Journal of Biomedical Informatics*, Vol. 156, p. 104683.

Wang, Ryan *et al.*, (2024). "Drop the shortcuts: image augmentation improves fairness and decreases AI detection of race and other demographics from medical images", *EBioMedicine*, Vol. 102.

Wang, Yuqing *et al.*, (2024). "FairEHR-CLP: Towards Fairness-Aware Clinical Predictions with Contrastive Learning in Multimodal Electronic Health Records", *arXiv preprint arXiv:2402.00955*,

Xia, Peng *et al.*, (2024). "CARES: A Comprehensive Benchmark of Trustworthiness in Medical Vision Language Models", *arXiv preprint arXiv:2406.06007*,

Xiao, Yuxin *et al.*, (2023). "In the name of fairness: assessing the bias in clinical record de-identification", *Proceedings of the 2023 ACM Conference on Fairness, Accountability, and Transparency*, pp. 123–137.

Yang, Ming Ying *et al.*, (2023). "Evaluating the Impact of Social Determinants on Health Prediction in the Intensive Care Unit", *Proceedings of the 2023 AAAI/ACM Conference on AI, Ethics, and Society*, pp. 333–350.

Yang, Yuzhe *et al.*, (2024). "The limits of fair medical imaging AI in real-world generalization", *Nature Medicine*, pp. 1–11.

Zemel, Rich *et al.*, (2013). "Learning fair representations", *International conference on machine learning*. PMLR, pp. 325–333.

Zhang, Daniel Yue, Kou, Ziyi, and Wang, Dong (2020). "Fairfl: A fair federated learning approach to reducing demographic bias in privacy-sensitive classification models", *2020 IEEE International Conference on Big Data (Big Data)*. IEEE, pp. 1051–1060.

Zhang, Jiaqing *et al.*, (2024). "Developing a fair and interpretable representation of the clock drawing test for mitigating low education and racial bias", *Scientific Reports*, Vol. 14 No. 1, p. 17444.

Zhang, Wenbin and Weiss, Jeremy C (2022). "Longitudinal fairness with censorship", *proceedings of the AAAI conference on artificial intelligence*. Vol. 36. No. 11, pp. 12235–12243.

Supplementary Material 4: Catalogue of fairness metrics

Table of Contents

Supplementary Table 4.1. Fairness Metrics, main properties, and classification according to different taxonomies 56
Supplementary Table 4.2. Operationalisation features of Fairness Metrics 61
Supplementary Table 4.3. Interpretation of Fairness Metrics 66
Supplementary Table 4.4. Critical Appraisal of Fairness Metrics 72

Supplementary Table 4.1. Fairness Metrics, main properties, and classification according to different taxonomies

This table reports all fairness metrics that were found in the literature.

- **id :** unique identifier that will be consistent for each metric across the document, especially to be used in the tables;
- **Metric name:** common name with which the metric is referred to, although other aliases may exist (see the next table);
- **Citation:** the original paper where the metric was *proposed* for the first time; *defined*, for example in a significant review; or *used* in a publication involving evaluation of clinical prediction models;
- **Defined for healthcare:** whether the metric was originally proposed in the healthcare domain and, if so, in which kind of modality;
- **Performance dependency:** whether the metric is dependent on performance (i.e, compares a pre-existing performance metric) or independent from performance (i.e, assesses the model output independently)
- **Model output type:** probability-based or threshold-independent
- **Performance metric domain:** taxonomy applied to the base performance metric, if applicable. Based on (1)
- **Fairness type:** individual or group

group fairness axiomatic criteria: also known as statistical non-discrimination; three criteria that aim to define the absence of discrimination in terms of statistical expressions and that can be associated with certain classification fairness metrics. Such criteria can be fulfilled “equivalently” or in a “relaxed” manner (127)

id	Metric Name	Citation	Defined for healthcare?	Performance Dependency	Model output type	Performance Metric(1)	Fairness Type	Group Axiomatic Fairness Criteria (44)
**1.1.0.1**	Mean Score Parity	(63) (defined)	No	Performance independent	Probability based	N/A	Group	Equivalent Independence
**1.1.0.2**	Unsupervised Ranking Fairness (URF)	(56) (defined)	No	Performance independent	Probability based	N/A	Group	N/A
**1.1.0.3**	Regression Demographic Parity	(57) (proposed)	No	Performance independent	Probability based	N/A	Group	N/A
**1.1.0.4**	Survival Group Fairness	(58) (proposed)	Yes	Performance independent	Probability based	N/A	Group	N/A
**1.1.0.5**	Censoring-Based Group Fairness	(59) (proposed)	No	Performance independent	Probability based	N/A	Group	N/A
**1.1.0.6**	Survival Intersectional Fairness	(58) (proposed)	Yes	Performance independent	Probability based	N/A	Group	N/A
**1.1.0.7**	Survival Individual Fairness	(58) (proposed)	Yes	Performance independent	Probability based	N/A	Individual	N/A
**1.1.0.8**	Censoring-Based Individual Fairness	(59) (proposed)	No	Performance independent	Probability based	N/A	Individual	N/A
**1.2.0.1**	Statistical Parity	(60) (defined)	No	Performance independent	Threshold dependent	N/A	Group	Equivalent Independence
**1.2.0.2**	Multi-group Demographic Parity	(61) (proposed)	No	Performance independent	Threshold dependent	N/A	Group	Equivalent Independence
**1.2.0.3**	Generalised Demographic Parity (GDP)	(61) (proposed)	No	Performance independent	Threshold dependent	N/A	Group	Equivalent Independence
**1.2.0.4**	Statistical Parity Subgroup Fairness	(55) (proposed)	No	Performance independent	Threshold dependent	N/A	Group	Equivalent Independence
**1.2.0.5**	Conditional Statistical Parity	(41) (defined)	No	Performance independent	Threshold dependent	N/A	Group	Relaxed Independence
**1.2.0.6**	Disparate Impact	(62) (defined)	No	Performance independent	Threshold dependent	N/A	Group	Relaxed Independence
**1.2.0.7**	Differential Fairness	(55) (defined)	No	Performance independent	Threshold dependent	N/A	Group	N/A

**2.1.1.1**	AUROC Parity	(64) (used)	?	Performance dependent	Probability based	Discrimination	Group	N/A
**2.1.1.2**	AUPRC Parity	(65) (used)	? Used with EHR	Performance dependent	Probability based	Discrimination	Group	N/A
**2.1.1.3**	Cross-Area Under the ROC Curve (xAUROC)	(66) (proposed)	No	Performance dependent	Probability based	Discrimination	Group	N/A
**2.1.1.4**	xAUROC Disparity	(66) (proposed)	No	Performance dependent	Probability based	Discrimination	Group	N/A
**2.1.1.5**	Subgroup AUROC (sAUROC)	(67) (proposed)	Yes, Imaging	Performance dependent	Probability based	Discrimination	Group	N/A
**2.1.1.6**	Pairwise Ranking Fairness	(56) (proposed)	No	Performance dependent	Probability based	Discrimination	Group	N/A
**2.1.1.7**	PRF Disparity (ΔPRF)	(56) (proposed)	No	Performance dependent	Probability based	Discrimination	Group	N/A
**2.1.1.8**	Equity-Scaled AUROC	(51) (proposed)	Yes, Imaging	Performance dependent	Probability based	Discrimination	Group	N/A
**2.1.1.9**	Mean Performance-Scaled Disparity (PSD) for AUROC	(68) (proposed)	Yes, Imaging	Performance dependent	Probability based	Discrimination	Group	N/A
**2.1.1.10**	Maximum Performance-Scaled Disparity (PSD) for AUROC	(68) (proposed)	Yes, Imaging	Performance dependent	Probability based	Discrimination	Group	N/A
**2.1.1.11**	Concordance Imparity	(71) (proposed)	No	Performance dependent	Probability based	Discrimination	Group	N/A
**2.1.1.12**	AUROC parity ratio	(123)(proposed)	Yes, EHR	Performance dependent	Probability based	Discrimination	Group	N/A
**2.1.2.1**	Expected Calibration Error (ECE) Parity	(69) (used)	Yes, tabular	Performance dependent	Probability based	Calibration	Group	Relaxed Sufficiency
**2.1.2.2**	Calibration-in-the-large Parity	(70) (used)	? Used with EHR	Performance dependent	Probability based	Calibration	Group	Relaxed Sufficiency
**2.1.2.3**	Absolute Calibration Error Parity	(72) (used)	? Used with EHR	Performance dependent	Probability based	Calibration	Group	Relaxed Sufficiency

**2.1.2.4**	Equal Calibration	(73) (defined)	No	Performance dependent	Probability based	Calibration	Group	Equivalent Sufficiency
**2.1.2.5**	Well Calibration	(45) (defined)	No	Performance dependent	Probability based	Calibration	Group	Relaxed Sufficiency
**2.1.3.1**	Brier Score Parity	(65) (used)	? Used with EHR	Performance dependent	Probability based	Overall	Group	N/A
**2.1.3.2**	Log-Loss Parity	(72) (used)	? Used with EHR	Performance dependent	Probability based	Overall	Group	N/A
**2.1.3.3**	Earth Mover's Distance (EMD) for equivalent separation	(65) (used)	? Used with EHR	Performance dependent	Probability based	N/A	Group	Equivalent Separation
**2.1.3.4**	Balance for Positive Class	(73) (defined)	No	Performance dependent	Probability based	N/A	Group	Relaxed Separation
**2.1.3.5**	Balance for Negative Class	(73) (defined)	No	Performance dependent	Probability based	N/A	Group	Relaxed Separation
**2.2.1.1**	Equal Opportunity Difference	(34) (defined)	No	Performance dependent	Threshold dependent	Partial	Group	Relaxed Separation
**2.2.1.2**	Predictive Equality	(74) (defined)	No	Performance dependent	Threshold dependent	Partial	Group	Relaxed Separation
**2.2.1.3**	Equalised Odds	(34) (defined)	No	Performance dependent	Threshold dependent	Partial	Group	Equivalent Separation
**2.2.1.4**	Equalising Disincentives	(75) (proposed)	No	Performance dependent	Threshold dependent	Partial	Group	Relaxed Separation
**2.2.1.5**	Average Odds Difference	(76) (proposed)	No	Performance dependent	Threshold dependent	Partial	Group	Relaxed Separation
**2.2.1.6**	Average Disparity in Equalised Odds	(77) (proposed)	Yes, multimodal	Performance dependent	Threshold dependent	Partial	Group	Equivalent Separation
**2.2.1.7**	Predictive Parity	(78) (defined)	No	Performance dependent	Threshold dependent	Partial	Group	Relaxed Sufficiency
**2.2.1.8**	NPV Difference	(29) (defined)	No	Performance dependent	Threshold dependent	Partial	Group	Relaxed Sufficiency
**2.2.1.9**	Conditional Equality of Opportunity Gap (EOGap)	(79) (defined)	No	Performance dependent	Threshold dependent	Partial	Group	Relaxed Separation

**2.2.1.10**	Conditional Use Accuracy Equality	(80) (defined)	No	Performance dependent	Threshold dependent	Partial	Group	Equivalent Sufficiency
**2.2.1.11**	Recall Equality Difference	(81) (proposed)	Yes, NLP	Performance dependent	Threshold dependent	Partial	Group	N/A
**2.2.1.12**	Recall Disparity Ratio	(82) (defined)	Yes, speech analysis	Performance dependent	Threshold dependent	Partial	Group	N/A
**2.2.1.13**	Worst-Group Recall	(83) (defined)	No	Performance dependent	Threshold dependent	Partial	Group	N/A
**2.2.1.14**	Maximum Difference Recall	(81) (proposed)	Yes, NLP	Performance dependent	Threshold dependent	Partial	Group	N/A
**2.2.1.15**	Recall Intergroup Standard Deviation	(84) (proposed)	Yes, Federated EHR	Performance dependent	Threshold dependent	Partial	Group	N/A
**2.2.1.16**	Recall Coefficient of Variation	(65) (used)	? Used with EHR	Performance dependent	Threshold dependent	Partial	Group	N/A
**2.2.1.17**	Recall Between-group Generalised Entropy Index (GEI)	(85) (proposed)	No	Performance dependent	Threshold dependent	Partial	Group	N/A
**2.2.1.18**	Recall-HEAL	(86) (proposed)	Yes	Performance dependent	Threshold dependent	Partial	Group	N/A
**2.2.2.1**	Overall Accuracy Gap	(80) (defined)	No	Performance dependent	Threshold dependent	Summary	Group	N/A
**2.2.2.2**	Error Rate Ratio	(87) (used)	Yes, clinical notes	Performance dependent	Threshold dependent	Summary	Group	N/A
**2.2.2.3**	Balanced Accuracy Difference	(29) (defined)	No	Performance dependent	Threshold dependent	Summary	Group	N/A
**2.2.2.4**	F1 Parity	(52) (proposed)	No	Performance dependent	Threshold dependent	Summary	Group	N/A
**2.2.2.5**	Matthew's Correlation Coefficient (MCC) Parity	(88) (defined)	Yes, tabular	Performance dependent	Threshold dependent	Summary	Group	N/A
**2.2.2.6**	Treatment Equality	(80) (defined)	No	Performance dependent	Threshold dependent	Summary	Group	N/A
**2.2.2.7**	Error Distribution Disparity Index (EDDI)	(77) (proposed)	Yes, multimodal	Performance dependent	Threshold dependent	Summary	Group	N/A
**2.2.3.1**	Subgroup Net Benefit	(89)(proposed)	Yes, tabular	Performance dependent	Threshold dependent	Clinical Utility	Group	N/A

Supplementary Table 4.2. Operationalisation features of Fairness Metrics

This table focuses on how to operationalise / compute each of the metrics that were found in our scoping review. Several columns are outlined:

- **<u>id</u>**: unique identifier that will be consistent for each metric across the document, especially to be used in the tables;
- **<u>metric name</u>**: common name with which the metric is referred to, although other aliases may exist;
- **<u>aliases</u>**: alternative names for the same metric;
- **<u>parent fairness metric</u>**: original fairness metric from which the present metric was (potentially) derived;
- **<u>base prediction type / performance metric:</u>** type of model prediction that the metric takes as an input (either an estimated probability or threshold-dependent predicted class) or base performance metric that is computed across groups and then compared for the sake of fairness (any ML performance metric is eligible);
- **<u>use of ground-truth:</u>** whether the metric considers the actual ground-truth (or label) present in the dataset. This will have implications on whether the metric is bias-preserving (i.e., accepts the *status quo*) or bias-transforming (i.e., aim for substantive equality and challenge existing historical labels that may be biased) (53)
- **<u>conditioning:</u>** whether the metric considers the features used in the model training to create strata and compare groups of patients no only according to a sensitive attribute but also according to such features;
- **<u>sensitive attribute:</u>** type of sensitive attribute that the metric supports. Can be binary, multi-group, or continuous.

id	Metric Name	Aliases	Parent Fairness Metric	Base Prediction Type / Performance Metric	Use of ground truth Y	Conditioning	Sensitive Attribute
**1.1.0.1**	Mean Score Parity	Average Score Parity; Mean Difference	N/A	Estimated Probability	No Y	No X	Binary
**1.1.0.2**	Unsupervised Ranking Fairness (URF)		N/A	Estimated Probability	No Y	No X	Binary
**1.1.0.3**	Regression Demographic Parity		Statistical Parity	Estimated Probability	No Y	No X	Multi-group
**1.1.0.4**	Survival Group Fairness		Statistical Parity	Estimated Probability	No Y	No X	Multi-group
**1.1.0.5**	Censoring-Based Group Fairness		Statistical Parity	Estimated Probability	No Y	Uses X	Multi-group
**1.1.0.6**	Survival Intersectional Fairness		Statistical Parity	Estimated Probability	Uses Y	No X	Multi-group
**1.1.0.7**	Survival Individual Fairness		N/A	Estimated Probability	No Y	Uses X	Multi-group
**1.1.0.8**	Censoring-Based Individual Fairness		N/A	Estimated Probability	No Y	Uses X	Multi-group
**1.2.0.1**	Statistical Parity	Demographic Parity, Statistical Parity Difference, Thresholded Score Parity	Mean Score Parity	Predicted Class	No Y	No X	Binary
**1.2.0.2**	Multi-group Demographic Parity		Statistical Parity	Predicted Class	No Y	No X	Multi-group
**1.2.0.3**	Generalised Demographic Parity (GDP)		Statistical Parity	Predicted Class	No Y	No X	Continuous
**1.2.0.4**	Statistical Parity Subgroup Fairness		Statistical Parity	Predicted Class	No Y	No X	Multi-group
**1.2.0.5**	Conditional Statistical Parity	Conditional principal fairness, Conditional demographic parity	Statistical Parity	Predicted Class	No Y	Uses X	Binary
**1.2.0.6**	Disparate Impact		N/A	Predicted Class	No Y	No X	Binary
**1.2.0.7**	Differential Fairness		N/A	Predicted Class	No Y	No X	Multi-group
**2.1.1.1**	AUROC Parity		N/A	AUROC	Uses Y	No X	Binary
**2.1.1.2**	AUPRC Parity		N/A	AUPRC	Uses Y	No X	Binary
**2.1.1.3**	Cross-Area Under the ROC Curve (xAUROC)		N/A	Estimated Probability	Uses Y	No X	Binary
**2.1.1.4**	xAUROC Disparity		xAUROC	xAUROC	Uses Y	No X	Binary
**2.1.1.5**	Subgroup AUROC (sAUROC)		N/A	Estimated Probability	Uses Y	No X	Binary

**2.1.1.6**	Pairwise Ranking Fairness		N/A	Estimated Probability	Uses Y	No X	Binary
**2.1.1.7**	PRF Disparity (ΔPRF)		PRF	PRF	Uses Y	No X	Binary
**2.1.1.8**	Equity-Scaled AUROC		N/A	AUROC	Uses Y	No X	Multi-group
**2.1.1.9**	Mean Performance-Scaled Disparity (PSD) for AUROC		N/A	AUROC	Uses Y	No X	Multi-group
**2.1.1.10**	Maximum Performance-Scaled Disparity (PSD) for AUROC		N/A	AUROC	Uses Y	No X	Multi-group
**2.1.1.11**	Concordance Imparity		N/A	Predicted Class	Uses Y	No X	Multi-group
**2.1.1.12**	AUROC parity ratio		N/A	AUROC	Uses Y	No X	Binary
**2.1.2.1**	Expected Calibration Error (ECE) Parity		N/A	ECE	Uses Y	No X	Binary
**2.1.2.2**	Calibration-in-the-large Parity		N/A	Calibration in the large	Uses Y	No X	Binary
**2.1.2.3**	Absolute Calibration Error Parity		N/A	Absolute Calibration Error	Uses Y	No X	Binary
**2.1.2.4**	Equal Calibration		N/A	Estimated Probability	Uses Y	No X	Binary
**2.1.2.5**	Well Calibration	Test-fairness; Matching Conditional Frequencies	Equal Calibration	Estimated Probability	Uses Y	No X	Binary
**2.1.3.1**	Brier Score Parity		N/A	Brier Score	Uses Y	No X	Binary
**2.1.3.2**	Log-Loss Parity		N/A	Log-Loss	Uses Y	No X	Binary
**2.1.3.3**	Earth Mover's Distance (EMD) for equivalent separation	First Wasserstein Distance, Optimal Transport	N/A	Estimated Probability	Uses Y	No X	Multi-group
**2.1.3.4**	Balance for Positive Class		N/A	Estimated Probability	Uses Y	No X	Binary
**2.1.3.5**	Balance for Negative Class		N/A	Estimated Probability	Uses Y	No X	Binary
**2.2.1.1**	Equal Opportunity Difference	Recall Parity (or Gap), False Negative Error Rate Balance.	N/A	TPR	Uses Y	No X	Binary
**2.2.1.2**	Predictive Equality	False Positive Error Rate Balance, Specificity Parity	N/A	FPR ≡TNR	Uses Y	No X	Binary
**2.2.1.3**	Equalised Odds	Conditional procedure accuracy, Disparity mistreatment	N/A	TPR & FPR	Uses Y	No X	Binary

**2.2.1.4**	Equalising Disincentives		N/A	TPR & FPR	Uses Y	No X	Binary
**2.2.1.5**	Average Odds Difference		Equalised Odds	TPR & FPR	Uses Y	No X	Binary
**2.2.1.6**	Average Disparity in Equalised Odds		Equalised Odds	TPR & FPR	Uses Y	No X	Multi-group
**2.2.1.7**	Predictive Parity	Outcome test	N/A	PPV	Uses Y	No X	Binary
**2.2.1.8**	NPV Difference		N/A	NPV	Uses Y	No X	Binary
**2.2.1.9**	Conditional Equality of Opportunity Gap (EOGap)		Equalised Odds	TPR	Uses Y	Uses X	Binary
**2.2.1.10**	Conditional Use Accuracy Equality		N/A	PPV & NPV	Uses Y	No X	Binary
**2.2.1.11**	Recall Equality Difference		Equal Opportunity	TPR	Uses Y	No X	Multi-group
**2.2.1.12**	Recall Disparity Ratio		Equal Opportunity	TPR	Uses Y	No X	Multi-group
**2.2.1.13**	Worst-Group Recall		N/A	TPR	Uses Y	No X	Multi-group
**2.2.1.14**	Maximum Difference Recall		Equal Opportunity	TPR	Uses Y	No X	Multi-group
**2.2.1.15**	Recall Intergroup Standard Deviation	Recall Parity Standard Deviation	N/A	TPR	Uses Y	No X	Multi-group
**2.2.1.16**	Recall Coefficient of Variation		GEI	TPR	Uses Y	No X	Multi-group
**2.2.1.17**	Recall Between-group Generalised Entropy Index (GEI)		N/A	TPR	Uses Y	No X	Multi-group
**2.2.1.18**	Recall-HEAL		N/A	TPR	Uses Y	No X	Multi-group
**2.2.2.1**	Overall Accuracy Gap		N/A	Accuracy	Uses Y	No X	Binary
**2.2.2.2**	Error Rate Ratio	Zero-one loss ratio	N/A	Error Rate	Uses Y	No X	Binary
**2.2.2.3**	Balanced Accuracy Difference		N/A	Balanced Accuracy	Uses Y	No X	Binary
**2.2.2.4**	F1 Parity	F1 Parity	N/A	F1-Score	Uses Y	No X	Binary
**2.2.2.5**	Matthew's Correlation Coefficient (MCC) Parity		N/A	MCC	Uses Y	No X	Binary

**2.2.2.6**	Treatment Equality	Error rate balance	N/A	FP/FN	Uses Y	No X	Binary
**2.2.2.7**	Error Distribution Disparity Index (EDDI)		N/A	Error Rate	Uses Y	No X	Multi-group
**2.2.3.1**	Subgroup Net Benefit		N/A	TPR & FPR	Use U	No X	Multi-group

Supplementary Table 4.3. Interpretation of Fairness Metrics

This table analyses each of the metrics from an interpretation and implications perspective. The following are studied:

- **description:** short summarisation of the metric
- **bias-preserving vs bias-transforming**: whether the metric is bias-preserving (i.e accepts the status quo) or bias-transforming (i.e aim for substantive equality and challenge existing historical labels that may be biased);
- **prevalence-dependency**: whether the base prevalence per sensitive attribute group
- **target value**: value that represents "perfect fairness";
- **direction to be fairer**: direction that the metric takes when fairness is improved, towards a target value;
- **potential legal or ethical connection**: key concepts or notions of fairness in the literature or in society that can be linked to each metric. Sparsely populated because not all metrics are morally or legally grounded.

Abbreviations: AUROC, area under the receiver operating characteristic curve; AUPRC, area under the precision recall curve; TPR, true positive rate; FPR, false positive rate; PPV, positive predictive value; NPV, negative predictive value; FP, false positive; FN, false negative

id	Metric Name	Description	Bias preserving or transforming? (53)	Depends on prevalence?	Target Value	Direction to be fairer	Potential legal or ethical connection
**1.1.0.1**	Mean Score Parity	Difference in estimated probability across groups	Bias transforming	Yes	0	Lower	
**1.1.0.2**	Unsupervised Ranking Fairness (URF)	Disparity in the likelihood of each group's estimated probability ranking higher than the other	Bias transforming	No	0	Lower	
**1.1.0.3**	Regression Demographic Parity	Id. mean score parity, with extension to continuous outcomes and more than 2 protected groups	Bias transforming	Yes	0	Lower	
**1.1.0.4**	Survival Group Fairness	Worst-case deviation of the per-group expected hazard function from the population average hazard	Bias transforming	Yes	0	Lower	
**1.1.0.5**	Censoring-Based Group Fairness	Id. mean score parity, within groups of censored and uncensored individuals	Bias transforming	Yes	0	Lower	
**1.1.0.6**	Survival Intersectional Fairness	Difference in the log-ratios of per-group hazard functions between intersectional subgroup	Bias preserving	Yes	0	Lower	Intersectionality
**1.1.0.7**	Survival Individual Fairness	Difference in hazard scores among individuals deemed similar based on a defined distance metric	Bias transforming	Yes	0	Lower	
**1.1.0.8**	Censoring-Based Individual Fairness	Id. survival individual fairness, within groups of censored and uncensored individuals	Bias transforming	Yes	0	Lower	
**1.2.0.1**	Statistical Parity	Difference in proportion of predicted classes after applying the threshold to the estimated risks	Bias transforming	Yes	0	Lower	EU's non-discrimination law
**1.2.0.2**	Multi-group Demographic Parity	Id. statistical parity, generalised for more than two categorical protected groups	Bias transforming	Yes	0	Lower	
**1.2.0.3**	Generalised Demographic Parity (GDP)	Id. statistical parity, generalised for continuous sensitive attributes	Bias transforming	Yes	0	Lower	
**1.2.0.4**	Statistical Parity Subgroup Fairness	Id. statistical parity, weighted by each subgroup representation	Bias transforming	Yes	0	Lower	
**1.2.0.5**	Conditional Statistical Parity	Id. statistical parity, stratifying for limited sets of "legitimate" features (conditioning)	Bias transforming	Yes	0	Lower	Contextual equality in non-discrimination law in the EU (92)

**1.2.0.6**	Disparate Impact	Proportion of positive predictions across groups	Bias transforming	Yes	0 (< 0.2)	Lower	Four-Fifths Rule, Anti-discrimination Law and Unintentional Bias
**1.2.0.7**	Differential Fairness	Proportion of logarithm positive predictions between groups	Bias transforming	Yes	range (e^−ϵ, e^ϵ)	Bidirectional	
**2.1.1.1**	AUROC Parity	Difference of AUROC (probability that an individual with the event has a higher estimated probability than an individual without the event) between groups	Bias preserving	No	0	Lower	
**2.1.1.2**	AUPRC Parity	Difference of AUPRC (precision integrated over recall) between groups	Bias preserving	No	0	Lower	
**2.1.1.3**	Cross-Area Under the ROC Curve (xAUROC)	TPR of group b (positive instances) integrated over the FPR of group a (negative instances) across various thresholds	Bias preserving	No	0.5	Bidirectional	
**2.1.1.4**	xAUROC Disparity	Difference of xAUROC(a, b) and xAUROC(b, a)	Bias preserving	No	0	Lower	
**2.1.1.5**	Subgroup AUROC (sAUROC)	TPR of population integrated over FPR subgroup	Bias preserving	No	0.5	Bidirectional	
**2.1.1.6**	Pairwise Ranking Fairness	Probability that a randomly selected positive instance from a group a is ranked higher than a randomly selected negative instance from either group a or another group b [PRF(a)]	Bias preserving	No	1	Higher	
**2.1.1.7**	PRF Disparity (ΔPRF)	Absolute difference in pairwise ranking fairness between two groups a and b [PRF(a) - PRF(b)]	Bias preserving	No	0	Lower	
**2.1.1.8**	Equity-Scaled AUROC	AUROC divided by the sum of the differences of each group's AUROC and the population's AUROC	Bias preserving	No	AUROC	Higher	
**2.1.1.9**	Mean Performance-Scaled Disparity (PSD) for AUROC	Mean disparity in AUROC performance across subgroups, scaled by the population's AUROC	Bias preserving	No	0	Lower	
**2.1.1.10**	Maximum Performance-Scaled Disparity (PSD) for AUROC	Maximum disparity in AUROC performance across subgroups, scaled by the population's AUROC	Bias preserving	No	0	Lower	

**2.1.1.11**	Concordance Imparity	Largest deviation among weighted group's sAUROC, in the presence of censorship	Bias preserving	No	0	Lower	
**2.1.1.12**	AUROC parity ratio	Ratio of the AUROC of the protected group to the AUROC of the reference group	Bias preserving	No	1	Higher	
**2.1.2.1**	Expected Calibration Error (ECE) Parity	Parity of ECE (weighted mean absolute difference between average probability and observed proportion from risk groups) across groups	Bias preserving	Yes	0	Lower	
**2.1.2.2**	Calibration-in-the-large Parity	Parity of calibration-in-the-large (difference between mean estimated probability and observed prevalence) across groups	Bias preserving	Yes	0	Lower	
**2.1.2.3**	Absolute Calibration Error Parity	Parity of absolute calibration error (absolute difference between mean estimated probability and observed prevalence) across groups	Bias preserving	Yes	0	Lower	
**2.1.2.4**	Equal Calibration	Difference of observed outcome rate between groups, for each estimated probability value	Bias preserving	No	0	Lower	
**2.1.2.5**	Well Calibration	Id. equal calibration, requiring that the actual probability of being in the positive class not only matches the estimated probability across all groups, but also target probability	Bias preserving	Yes	0	Lower	
**2.1.3.1**	Brier Score Parity	Parity of Brier score (average squared difference between the outcome labels and the estimated probabilities) across groups	Bias preserving	Yes	0	Lower	
**2.1.3.2**	Log-Loss Parity	Parity of log-loss (negative loglikelihood) across groups	Bias preserving	Yes	0	Lower	
**2.1.3.3**	Earth Mover's Distance (EMD) for equivalent separation	First Wasserstein distance between the distribution of estimated probabilities across groups, conditioned on the expected outcome	Bias preserving	Yes	0	Lower	
**2.1.3.4**	Balance for Positive Class	Difference in mean estimated probability across groups, conditioned on positive expected outcome	Bias preserving	No	0	Lower	
**2.1.3.5**	Balance for Negative Class	Difference in mean estimated probability across groups, conditioned on negative expected outcome	Bias preserving	No	0	Lower	
**2.2.1.1**	Equal Opportunity Difference	Absolute difference in sensitivity/recall/TPR (proportion of individuals with an event that are correctly classified) across groups	Bias preserving	No	0	Lower	

**2.2.1.2**	Predictive Equality	Absolute difference in specificity/FPR (proportion of individuals without event that are correctly classified) across groups	Bias preserving	No	0	Lower	
**2.2.1.3**	Equalised Odds	"Aggregation" (e.g, mean, maximum, minimum) of equality of opportunity difference and predictive equality	Bias preserving	No	0	Lower	
**2.2.1.4**	Equalising Disincentives	Difference between groups of the difference of TPR and FPR	Bias preserving	No	0	Lower	
**2.2.1.5**	Average Odds Difference	Average difference between FPR and TPR across groups	Bias preserving	No	0	Lower	
**2.2.1.6**	Average Disparity in Equalised Odds	Id. equalised odds, generalised for several groups	Bias preserving	No	0	Lower	
**2.2.1.7**	Predictive Parity	Absolute difference in PPV/precision (proportion of high risk individuals that have an event) across groups	Bias preserving	No	0	Lower	
**2.2.1.8**	NPV Difference	Absolute difference in NPV (proportion of low risk individuals that do not have an event) across groups	Bias preserving	No	0	Lower	
**2.2.1.9**	Conditional Equality of Opportunity Gap (EOGap)	Id. equality of opportunity difference, stratifying for limited sets of "legitimate" features (conditioning)	Bias preserving	No	0	Lower	
**2.2.1.10**	Conditional Use Accuracy Equality	Id. equalised odds, but using PPV and NPV (instead of TPR and FPR)	Bias preserving	No	0	Lower	
**2.2.1.11**	Recall Equality Difference	Id. equality of opportunity difference, generalised to multiple groups, computing the absolute difference between each group's recall and the population recall	Bias preserving	No	0	Lower	
**2.2.1.12**	Recall Disparity Ratio	Ratio of recall between the group with the highest<br>recall and the group with the lowest recall	Bias preserving	No	1	Higher	
**2.2.1.13**	Worst-Group Recall	Minimum recall across all groups	Bias preserving	No	Max Recall	Higher	John Rawls' Min-Max Fairness
**2.2.1.14**	Maximum Difference Recall	Largest absolute difference between a group's recall and the population recall	Bias preserving	No	0	Lower	
**2.2.1.15**	Recall Intergroup Standard Deviation	Standard deviation of groups' recall	Bias preserving	No	0	Lower	

**2.2.1.16**	Recall Coefficient of Variation	Ratio of the standard deviation of groups' recall to its mean	Bias preserving	No	0	Lower	
**2.2.1.17**	Recall Between-group Generalised Entropy Index (GEI)	Generalised entropy index applied to each group's recall	Bias preserving	No	0	Lower	Somewhat Egalitarianism
**2.2.1.18**	Recall-HEAL	Anticorrelation between each group's recall and a health outcome of interested (obtained from external data)	Bias transforming	No	1	Higher	(Historical) Reparations
**2.2.2.1**	Overall Accuracy Gap	Absolute difference in accuracy (proportion of correctly classified individuals) across groups	Bias preserving	Yes	0	Lower	
**2.2.2.2**	Error Rate Ratio	Absolute difference in error rate (proportion of incorrectly classified individuals) across groups	Bias preserving	Yes	1	Higher	
**2.2.2.3**	Balanced Accuracy Difference	Absolute difference in balanced accuracy (mean of sensitivity and specificity) across groups	Bias preserving	No	0	Lower	
**2.2.2.4**	F1 Parity	Absolute difference in F1-score (harmonic mean of precision and recall) across groups	Bias preserving	Yes	0	Lower	
**2.2.2.5**	Matthew's Correlation Coefficient (MCC) Parity	Absolute difference in MCC (phi correlation between the model's classifications and the actual outcomes) across groups	Bias preserving	Yes	0	Lower	
**2.2.2.6**	Treatment Equality	Difference in the ratio of FP to FN between groups	Bias preserving	No	0	Lower	
**2.2.2.7**	Error Distribution Disparity Index (EDDI)	Deviation of error rates across groups relative to the population error rate	Bias preserving	Yes	0	Lower	
**2.2.3.1**	Subgroup Net Benefit	Expected benefit in terms of TN within a subgroup, accounting for the prevalence of the outcome, TPR and FPR at a decision threshold, and the relative benefit of treating TP versus avoiding unnecessary treatment	Bias preserving	No	(1-Prevalence)	Higher	

Supplementary Table 4.4. Critical Appraisal of Fairness Metrics

Critical appraisal of each identified metric is performed according to the following angles:

- **justified use:** scenarios in which assessing a model according to that metric is advisable;
- **example in a clinical prediction model:** clinical prediction model in which the use of the metric fits its justified use;
- **direct harm:** potential harm to the patient when a metric is not satisfied or is not as good as it should be;
- **comment:** open questions and further comments.

id	Metric Name	Justified Use	Example in a clinical prediction model	Direct harm to patients	Comment
**1.1.0.1**	Mean Score Parity	Similar to statistical parity but at the risk level; provides the advantage of assessing non-threshold-dependent risks	Predicting Atherosclerotic Cardiovascular Disease Risk	Patients with similar actual risk may have different risk estimates depending on group membership	Has the merit of not imposing a decision threshold and working on the risk-level
**1.1.0.2**	Unsupervised Ranking Fairness (URF)	When the patients' risk ranking is relevant regardless of ground-truth and a single decision threshold is shared across subgroups of patients	An organ transplant assignment would ideally satisfy ranking fairness in an unsupervised manner, thus challenging the status quo of historical biases		Unclear implications in relation with other metrics
**1.1.0.3**	Regression Demographic Parity	id. statistical parity	id. statistical parity	id. statistical parity	
**1.1.0.4**	Survival Group Fairness	id. statistical parity			
**1.1.0.5**	Censoring-Based Group Fairness				
**1.1.0.6**	Survival Intersectional Fairness				
**1.1.0.7**	Survival Individual Fairness				
**1.1.0.8**	Censoring-Based Individual Fairness				
**1.2.0.1**	Statistical Parity	When model development data is expected to be prejudicial (or ground-truths have not been agreed upon) and parity should be expected	Predicting future health expenditure (Obermeyer 2019); if this metric had been used, the reported bias would have been mitigated	Lack of selection or positive prediction	As a bias preserving metric (Wacther et al. 2021), a 100% accurate model can be deemed unfair according to this metric. On the other hand, a dummy classifier that predicts '1' for all subjects would be 100% fair
**1.2.0.2**	Multi-group Demographic Parity	id. statistical parity	id. statistical parity	id. statistical parity	
**1.2.0.3**	Generalised Demographic Parity (GDP)	id. statistical parity	id. statistical parity	id. statistical parity	

**1.2.0.4**	Statistical Parity Subgroup Fairness	id. statistical parity	id. statistical parity	id. statistical parity	
**1.2.0.5**	Conditional Statistical Parity	When a bias transforming metric is required and there are clear strata of patients according to which disparate predictions are expected to take place	id. statistical parity	Lack of selection or differential positivity rate across subgroups, even after stratifying according to more relevant covariates	Considered a “gold standard” for fairness automation (92) that aligns with the aims of EU non-discrimination legislation. Since the metric is conditioned on strata, it inherently relies on “contextual evaluations”, which may be more meaningful in complex societal and clinical scenarios. Determining "legitimate" features is a nontrivial process and may require speculating about the underlying causal pathways
**1.2.0.6**	Disparate Impact	When compliance with the disparate impact law is required; similar to statistical parity but in the form of a ratio and with a reference value determined by law (20%)	id. statistical parity	id. statistical parity	Its (US) legal origin may make it attractive to regulators and quantify a model's output in legal terms
**1.2.0.7**	Differential Fairness	Unclear			
**2.1.1.1**	AUROC Parity	When similar discrimination across groups is desirable, which is most often the case		Lack of discrimination	
**2.1.1.2**	AUPRC Parity	Unclear		Lack of discrimination	
**2.1.1.3**	Cross-Area Under the ROC Curve (xAUROC)	Unclear		Unfair discrimination and ranking of risks	Unclear how this relates to the traditional AUROC
**2.1.1.4**	xAUROC Disparity	Unclear		Unfair discrimination and ranking of risks	Unclear how this relates to the traditional AUROC
**2.1.1.5**	Subgroup AUROC (sAUROC)	Unclear		Unfair discrimination and ranking of risks	Unclear how this relates to the traditional AUROC
**2.1.1.6**	Pairwise Ranking Fairness	Unclear		Unfair discrimination and ranking of risks	Unclear how this relates to traditional discrimination metrics
**2.1.1.7**	PRF Disparity (ΔPRF)	Unclear		Unfair discrimination and ranking of risks	

**2.1.1.8**	Equity-Scaled AUROC	Unclear; potentially useful to compare models but clinical applicability is unclear	Glaucoma screening (51)	Lack of discrimination	Regarded by the authors who propose it as a more comprehensive metric that combines both performance and equity performance. However, this metric may add very little compared to an AUROC parity or a similar metric; an ES-AUROC carries little meaning when not reported together with a non-equity-scaled-AUROC that can be used for baseline comparison. On the other hand, a very inequitable model that has a very high baseline AUROC could still be deemed "fair", as the high baseline performance mitigates the impact of the unfairness. There is no in-depth study around these questions and potential misbehaviours of this metric
**2.1.1.9**	Mean Performance-Scaled Disparity (PSD) for AUROC	Unclear		Lack of discrimination	
**2.1.1.10**	Maximum Performance-Scaled Disparity (PSD) for AUROC	Unclear		Lack of discrimination	
**2.1.1.11**	Concordance Imparity	When censorship is present and discrimination is desirable		Lack of discrimination	Insufficient empirical evaluation
**2.1.1.12**	AUROC parity ratio	When within-group discrimination is expected to be as similar as possible	Validation of a Proprietary Deterioration Index Model and Performance in Hospitalized Adults	Lack of discrimination	Insufficient motivation and evaluation
**2.1.2.1**	Expected Calibration Error (ECE) Parity			Lack of calibration	
**2.1.2.2**	Calibration-in-the-large Parity			Lack of calibration	

**2.1.2.3**	Absolute Calibration Error Parity			Lack of calibration	
**2.1.2.4**	Equal Calibration	When ensuring good calibration (independent from the decision threshold) across groups is deemed necessary	Predicting Atherosclerotic Cardiovascular Disease Risk and compare model's risks with guideline's risks	Lack of calibration, resulting in uneven under- or over-estimation of risk	
**2.1.2.5**	Well Calibration	When there is a reference risk threshold defined in medical guidelines and it its important that all subgroups are well calibrated in respect to that reference value	See Equal Calibration. In this case the actual predicted risk would be compared with medical guidelines	Lack of calibration	
**2.1.3.1**	Brier Score Parity	Unclear			
**2.1.3.2**	Log-Loss Parity	Unclear			Usually used as a loss function and convenient to compare models. The behaviour of this metric is usually a good representation of a model's performance. Nevertheless, this metric is not necessarily interpretable in the context of a clinical prediction model and discrimination or calibration metrics can prove to be more useful or informative
**2.1.3.3**	Earth Mover's Distance (EMD) for equivalent separation	When the exact output probability distributions are expected to be matched across the different sensitive attribute groups			Strongest form of meeting the separation criterion; hard to interpret
**2.1.3.4**	Balance for Positive Class				
**2.1.3.5**	Balance for Negative Class				
**2.2.1.1**	Equal Opportunity Difference	When ground-truth is consensual and there is an "overwhelming" harm coming from false negatives	Screening programmes, reference to a specialist, or assignment to a limited resource (e.g organ transplant or hospital bed)	Fails to identify positive cases	The name of the metric may not make sense if the positive outcome class is related to an adverse outcome.
**2.2.1.2**	Predictive Equality	When ground-truth is consensual and there is an "overwhelming" harm coming from false positives	Surgery outcome prediction where there might be an adverse outcome (the adverse outcome being the positive class)	Fails to identify negative cases	
**2.2.1.3**	Equalised Odds	When it is desirable to combine TPR and FPR parity into the same metric	Clinical prediction model to decide whether to perform a risky surgery. Missing eligible	Not fully clear due to the duality of criteria in this	The way TPR & FPR parity is combined into a single metric is

			patients may be as harmful as selecting patients who do not benefit from the intervention, resulting in worsened outcomes in both types of errors	metric. Fails to identify both positive and negative cases.	not always consensual: can be taken as an average or as a max/min. The name "equalised odds" is also questionable given that probabilities are being used, rather than odds (distinct mathematical concepts)
**2.2.1.4**	Equalising Disincentives	Similar to equalised odds	id. equalised odds	id. equalised odds	
**2.2.1.5**	Average Odds Difference	id. equalised odds	id. equalised odds	id. equalised odds	
**2.2.1.6**	Average Disparity in Equalised Odds	id. equalised odds	id. equalised odds	id. equalised odds	
**2.2.1.7**	Predictive Parity	When resources are limited and all the positive predictions must be correct across different groups			
**2.2.1.8**	NPV Difference	When the reliability of a negative prediction is important to be correct across groups			
**2.2.1.9**	Conditional Equality of Opportunity Gap (EOGap)	When ground-truth is consensual, there is an overwhelming harm coming from FN, and a limited set of "legitimate" features could be used to stratification beyond the ones defined by the sensitive attributes	Predicting health needs in the future across sensitive attributes but also considering comorbidities as strata (22)		
**2.2.1.10**	Conditional Use Accuracy Equality	Combination of PPV and NPV parity		Not fully clear due to the duality of criteria in this metric. Fails prediction when prediction is both positive or negative	
**2.2.1.11**	Recall Equality Difference	See equality of opportunity, but extended to multiple groups on the sensitive attribute definition			
**2.2.1.12**	Recall Disparity Ratio	When the main concern for fairness is the performance range across all groups	There are several groups with respect to the sensitive attribute and there is it is desirable to minimise disparities across the board		
**2.2.1.13**	Worst-Group Recall	When the main concern for fairness is the minimum performance across all groups			
**2.2.1.14**	Maximum Difference Recall	When the main concern for fairness is the performance range across all groups			

**2.2.1.15**	Recall Intergroup Standard Deviation	When dispersion and high variance of performance across groups is not desirable			
**2.2.1.16**	Recall Coefficient of Variation	id. intergroup standard deviation			
**2.2.1.17**	Recall Between-group Generalised Entropy Index (GEI)	Unclear			The generalised entropy index as an inequality index can be decomposed as a sum of between-group and within-group inequality. Herein we just include the former as a group fairness metric; the implications of the latter as a metric are unclear, and might lean more towards individual fairness
**2.2.1.18**	Recall-HEAL	When reparations with respect to historical, more negative outcome burdens are looked for	A clinical prediction model to predict dermatological conditions from skin imaging (86). This metric would be satisfied if the model performance (TPR in this case) was the best among the patients with the worst health outcomes (disability-adjusted life years in the example)	The potential effect of levelling down patients who could have achieved better outcomes if not for the reparation justice that this metric implies	The implications of this metric are largely understudied
**2.2.2.1**	Overall Accuracy Gap	When ground-truth is agreed upon, the harm of misclassification is the same regardless of patients' ground-truth, and the distribution of such classes is globally "balanced"			The accuracy metric can easily be "fooled" depending on outcome distributions and decision thresholds
**2.2.2.2**	Error Rate Ratio	Same as overall accuracy gap			id. overall accuracy gap
**2.2.2.3**	Balanced Accuracy Difference	When the type of error does not necessarily matter (FP or FN) but the accuracy among positive and negative class individuals should be equally weighted	In predicting a rare outcome where a FP or a FN are equally harmful	Patients with rare outcomes may have disproportionate lower accuracy	
**2.2.2.4**	F1 Parity	Achieve overall classification parity through F1-score			F1-Score has been regarded as an inadvisable metric for performance assessment as it ignores TN, conflates classification with clinical utility and has no intuitive interpretation (1)

**2.2.2.5**	Matthew's Correlation Coefficient (MCC) Parity	Achieve overall classification parity through MCC			Like F1-Score, it is hard to interpret intuitively (1). Nevertheless, MCC has been suggested to provide the most comprehensive quantification of classification performance (88), and could be an interesting metric to base fairness metrics upon.
**2.2.2.6**	Treatment Equality	Unclear	Unclear	Unclear	
**2.2.2.7**	Error Distribution Disparity Index (EDDI)	When several groups exist and the disparity needs to be minimised			
**2.2.3.1**	Subgroup Net Benefit	When net benefit needs to be computed across subgroups and be comparable. Considering the specific implementation of this metric, utility is defined in terms of true negatives.	A diabetes prognostic model and a lung cancer screening algorithm	Unequal of utility for all patients.	

References

1. Van Calster B, Collins GS, Vickers AJ, Wynants L, Kerr KF, Barreñada L, et al. Performance evaluation of predictive AI models to support medical decisions: Overview and guidance. arXiv [cs.LG]. 2024. Available from: http://arxiv.org/abs/2412.10288

2. van Smeden M, Reitsma JB, Riley RD, Collins GS, Moons KG. Clinical prediction models: diagnosis versus prognosis. J Clin Epidemiol. 2021 Apr 1;132:142–5. Available from: http://dx.doi.org/10.1016/j.jclinepi.2021.01.009

3. Topol EJ. High-performance medicine: the convergence of human and artificial intelligence. Nat Med. 2019 Jan 7;25(1):44–56. Available from: https://www.nature.com/articles/s41591-018-0300-7

4. Wynants L, Van Calster B, Collins GS, Riley RD, Heinze G, Schuit E, et al. Prediction models for diagnosis and prognosis of covid-19: systematic review and critical appraisal. BMJ. 2020 Apr 7;369:m1328. Available from: https://www.bmj.com/content/369/bmj.m1328.abstract

5. Kappen TH, van Klei WA, van Wolfswinkel L, Kalkman CJ, Vergouwe Y, Moons KGM. Evaluating the impact of prediction models: lessons learned, challenges, and recommendations. Diagn Progn Res. 2018 Jun 12; 2(1):11. Available from: https://pmc.ncbi.nlm.nih.gov/articles/PMC6460651/

6. Kanis JA, Oden A, Johnell O, Johansson H, De Laet C, Brown J, et al. The use of clinical risk factors enhances the performance of BMD in the prediction of hip and osteoporotic fractures in men and women. Osteoporos Int. 2007 Aug;18(8):1033–46. Available from: https://pubmed.ncbi.nlm.nih.gov/17323110/

7. Hippisley-Cox J, Coupland C, Brindle P. Development and validation of QRISK3 risk prediction algorithms to estimate future risk of cardiovascular disease: prospective cohort study. BMJ. 2017 May 23; 357:j2099. Available from: https://www.bmj.com/content/357/bmj.j2099.abstract

8. Andaur Navarro CL, Damen JAA, Takada T, Nijman SWJ, Dhiman P, Ma J, et al. Systematic review finds "spin" practices and poor reporting standards in studies on machine learning-based prediction models. J Clin Epidemiol. 2023 Jun; 158:99–110. Available from: https://pubmed.ncbi.nlm.nih.gov/37024020/

9. Dhiman P, Ma J, Navarro CA, Speich B, Bullock G, Damen JA, et al. Reporting of prognostic clinical prediction models based on machine learning methods in oncology needs to be improved. J Clin Epidemiol. 2021 Oct; 138:60–72. Available from: https://pubmed.ncbi.nlm.nih.gov/34214626/

10. Johnson AEW, Pollard TJ, Mark RG. Reproducibility in critical care: a mortality prediction case study. Doshi-Velez F, Fackler J, Kale D, Ranganath R, Wallace B, Wiens J, editors. 18--19 Aug 2017;68:361–76. Available from: https://proceedings.mlr.press/v68/johnson17a.html

11. Collins GS, Reitsma JB, Altman DG, Moons KGM. Transparent Reporting of a multivariable prediction model for Individual Prognosis Or Diagnosis (TRIPOD). Ann Intern Med. 2015 May 19; 162(10):735–6. Available from: https://pubmed.ncbi.nlm.nih.gov/25984857/

12. Collins GS, Moons KGM, Dhiman P, Riley RD, Beam AL, Van Calster B, et al. TRIPOD+AI statement: updated guidance for reporting clinical prediction models that use regression or machine learning methods. BMJ. 2024 Apr 16;385:e078378. Available from: https://www.bmj.com/content/385/bmj-2023-078378.abstract

13. Nazer LH, Zatarah R, Waldrip S, Ke JXC, Moukheiber M, Khanna AK, et al. Bias in artificial intelligence algorithms and recommendations for mitigation. PLOS Digit Health. 2023 Jun;2(6):e0000278. Available from: http://dx.doi.org/10.1371/journal.pdig.0000278

14. Nakayama LF, Matos J, Quion J, Novaes F, Mitchell WG, Mwavu R, et al. Unmasking biases and navigating pitfalls in the ophthalmic artificial intelligence lifecycle: A narrative review. PLOS Digit Health. 2024 Oct;3(10):e0000618. Available from: https://doi.org/10.1371/journal.pdig.0000618

15. Matos J, Gallifant J, Chowdhury A, Economou-Zavlanos N, Charpignon ML, Gichoya J, et al. A clinician's guide to understanding bias in critical clinical prediction models. Crit Care Clin. 2024 Oct; 40(4):827–57. Available from: https://pubmed.ncbi.nlm.nih.gov/39218488/

16. Andaur Navarro CL, Damen JAA, Takada T, Nijman SWJ, Dhiman P, Ma J, et al. Risk of bias in studies on prediction models developed using supervised machine learning techniques: systematic review. BMJ. 2021 Oct 20;375:n2281. Available from: https://www.bmj.com/content/375/bmj.n2281.abstract

17. Collins GS, Dhiman P, Ma J, Schlussel MM, Archer L, Van Calster B, et al. Evaluation of clinical prediction models

(part 1): from development to external validation. BMJ. 2024 Jan 8; 384:e074819. Available from: https://www.bmj.com/content/384/bmj-2023-074819.abstract

18. Yang Y, Zhang H, Gichoya JW, Katabi D, Ghassemi M. The limits of fair medical imaging AI in real-world generalization. Nature Medicine. 2024;1–11. Available from: https://www.nature.com/articles/s41591-024-03113-4

19. Panch T, Mattie H, Atun R. Artificial intelligence and algorithmic bias: implications for health systems. J Glob Health. 2019 Dec; 9(2):010318. Available from: http://dx.doi.org/10.7189/jogh.09.020318

20. Celi LA, Cellini J, Charpignon ML, Dee EC, Dernoncourt F, Eber R, et al. Sources of bias in artificial intelligence that perpetuate healthcare disparities—A global review. PLOS Digital Health. 2022 Mar 31; 1(3):e0000022. Available from: https://doi.org/10.1371/journal.pdig.0000022

21. Kirk JK, Passmore LV, Bell RA, Narayan KMV, D'Agostino RB Jr, Arcury TA, et al. Disparities in A1C levels between Hispanic and non-Hispanic white adults with diabetes: a meta-analysis. Diabetes Care. 2008 Feb; 31(2):240–6. Available from: https://pubmed.ncbi.nlm.nih.gov/17977939/

22. Obermeyer Z, Powers B, Vogeli C, Mullainathan S. Dissecting racial bias in an algorithm used to manage the health of populations. Vol. 366, Science. 2019. p. 447–53. Available from: http://dx.doi.org/10.1126/science.aax2342

23. Charpignon ML, Byers J, Cabral S, Celi LA, Fernandes C, Gallifant J, et al. Critical Bias in Critical Care Devices. Crit Care Clin. 2023 Oct;39(4):795–813. Available from: http://dx.doi.org/10.1016/j.ccc.2023.02.005

24. Martins I, Matos J, Gonçalves T, Celi LA, Wong AI, Cardoso JS. Evaluating the impact of pulse oximetry bias in machine learning under counterfactual thinking. arXiv [cs.LG]. 2024. Available from: https://link.springer.com/chapter/10.1007/978-3-031-82007-6_21

25. Participation E. Equality Act 2010. Available from: https://www.legislation.gov.uk/ukpga/2010/15/contents

26. Legislation summary - How is discrimination addressed in EU legislation?. Available from: https://www.eu-patient.eu/policy/Policy/Anti-discrimination/legislation-summary---how-is-discrimination-addressed-in-eu-legislation/

27. US Legal, Inc. Protected Group Member Law and Legal Definition. Available from: https://definitions.uslegal.com/p/protected-group-member/

28. Gallifant J, Bitterman DS, Celi LA, Gichoya JW, Matos J, McCoy LG, et al. Ethical debates amidst flawed healthcare artificial intelligence metrics. NPJ Digit Med. 2024 Sep 11; 7(1):243. Available from: https://www.nature.com/articles/s41746-024-01242-1

29. Jaime S, Kern C. Ethnic classifications in algorithmic fairness: Concepts, measures and implications in practice. The 2024 ACM Conference on Fairness, Accountability, and Transparency. 2024. p. 237–53. Available from: https://facctconference.org/static/papers24/facct24-17.pdf

30. Goetz L, Seedat N, Vandersluis R, van der Schaar M. Generalization-a key challenge for responsible AI in patient-facing clinical applications. NPJ Digit Med. 2024 May 21; 7(1):126. Available from: https://www.nature.com/articles/s41746-024-01127-3

31. Alderman JE, Palmer J, Laws E, McCradden MD, Ordish J, Ghassemi M, et al. Tackling algorithmic bias and promoting transparency in health datasets: the STANDING Together consensus recommendations. Lancet Digit Health. 2025 Jan 1; 7(1):e64–88. Available from: http://www.thelancet.com/article/S2589750024002243/abstract

32. Oakden-Rayner L, Dunnmon J, Carneiro G, Ré C. Hidden stratification causes clinically meaningful failures in machine learning for medical imaging. Proc ACM Conf Health Inference Learn. 2020 Apr; 2020:151–9. Available from: https://pmc.ncbi.nlm.nih.gov/articles/PMC7665161/

33. Mccradden M, Odusi O, Joshi S, Akrout I, Ndlovu K, Glocker B, et al. What's fair is… fair? Presenting JustEFAB, an ethical framework for operationalizing medical ethics and social justice in the integration of clinical machine learning: JustEFAB. 2023 ACM Conference on Fairness, Accountability, and Transparency. New York, NY, USA: ACM; 2023. p. 1505–19. Available from: https://dl.acm.org/doi/pdf/10.1145/3593013.3594096

34. Hardt M, Price E, Srebro N. Equality of opportunity in supervised learning. Advances in neural information processing systems. 2016;29. Available from: https://dl.acm.org/doi/10.5555/3157382.3157469

35. Chen J, Kallus N, Mao X, Svacha G, Udell M. Fairness under unawareness: Assessing disparity when protected class is unobserved. Proceedings of the Conference on Fairness, Accountability, and Transparency. New York, NY, USA: ACM; 2019. Available from: https://dl.acm.org/doi/abs/10.1145/3287560.3287594

36. Dwork C, Hardt M, Pitassi T, Reingold O, Zemel R. Fairness Through Awareness. arXiv [cs.CC]. 2011. Available from: http://arxiv.org/abs/1104.3913

37. Kusner MJ, Loftus JR, Russell C, Silva R. Counterfactual Fairness. arXiv [stat.ML]. 2017. Available from: http://arxiv.org/abs/1703.06856

38. Martinez N, Bertran M, Sapiro G. Minimax Pareto Fairness: A Multi Objective Perspective. International Conference on Machine Learning. PMLR; 2020. p. 6755–64. Available from: https://proceedings.mlr.press/v119/martinez20a.html

39. Lekadir K, Frangi AF, Porras AR, Glocker B, Cintas C, Langlotz CP, et al. FUTURE-AI: international consensus guideline for trustworthy and deployable artificial intelligence in healthcare. BMJ. 2025 Feb 5; 388:e081554. Available from: https://www.bmj.com/content/388/bmj-2024-081554.abstract

40. Verma S, Rubin J. Fairness definitions explained. Proceedings of the International Workshop on Software Fairness. New York, NY, USA: ACM; 2018. Available from: https://fairware.cs.umass.edu/papers/Verma.pdf

41. Corbett-Davies S, Gaebler JD, Nilforoshan H, Shroff R, Goel S. The measure and mismeasure of fairness. arXiv [cs.CY]. 2018. Available from: http://arxiv.org/abs/1808.00023

42. Pessach D, Shmueli E. A review on fairness in machine learning. ACM Computing Surveys (CSUR). 2022;55(3):1–44. Available from: https://dl.acm.org/doi/10.1145/3494672

43. Mehrabi N, Morstatter F, Saxena N, Lerman K, Galstyan A. A survey on bias and fairness in machine learning. ACM Comput Surv. 2022 Jul 31; 54(6):1–35. Available from: https://dl.acm.org/doi/10.1145/3457607

44. Barocas S, Hardt M, Narayanan A. Fairness and machine learning: Limitations and opportunities. MIT press; 2023. Available from: https://fairmlbook.org/

45. Caton S, Haas C. Fairness in Machine Learning: A survey. ACM Comput Surv. 2024 Jul 31;56(7):1–38. Available from: https://dl.acm.org/doi/pdf/10.1145/3616865

46. Anderson JW, Visweswaran S. Algorithmic individual fairness and healthcare: a scoping review. JAMIA Open. 2025 Feb; 8(1):ooae149. Available from: https://academic.oup.com/jamiaopen/article-pdf/8/1/ooae149/61297239/ooae149.pdf

47. Mienye ID, Swart TG, Obaido G. Fairness Metrics in AI Healthcare Applications: A Review. In: 2024 IEEE International Conference on Information Reuse and Integration for Data Science (IRI). IEEE; 2024. p. 284–9. Available from: https://ieeexplore.ieee.org/document/10703970

48. Arksey H, O'Malley L. Scoping studies: towards a methodological framework. Int J Soc Res Methodol. 2005 Feb 1; 8(1):19–32. Available from: https://www.tandfonline.com/doi/abs/10.1080/1364557032000119616

49. Tricco AC, Lillie E, Zarin W, O'Brien KK, Colquhoun H, Levac D, et al. PRISMA extension for Scoping Reviews (PRISMA-ScR): Checklist and explanation. Ann Intern Med. 2018 Oct 2; 169(7):467–73. Available from: https://www.acpjournals.org/doi/10.7326/M18-0850

50. Franklin JS, Bhanot K, Ghalwash M, Bennett KP, McCusker J, McGuinness DL. An ontology for fairness metrics. In: Proceedings of the 2022 AAAI/ACM Conference on AI, Ethics, and Society. New York, NY, USA: ACM; 2022. Available from: https://dl.acm.org/doi/pdf/10.1145/3514094.3534137

51. Luo Y, Tian Y, Shi M, Pasquale LR, Shen LQ, Zebardast N, et al. Harvard glaucoma fairness: a retinal nerve disease dataset for fairness learning and fair identity normalization. IEEE Transactions on Medical Imaging. 2024 Jul 1;43(7):2623–2633. Available from: https://ieeexplore.ieee.org/document/10472539

52. Zhang DY, Kou Z, Wang D. Fairfl: A fair federated learning approach to reducing demographic bias in privacy-sensitive classification models. 2020 IEEE International Conference on Big Data (Big Data). IEEE; 2020. p. 1051–60. Available from: https://ieeexplore.ieee.org/document/9378043

53. Wachter S, Mittelstadt B, Russell C. Bias preservation in machine learning: The legality of fairness metrics under EU non-discrimination law. SSRN Electron J. 2021 Jan 15; Available from: https://papers.ssrn.com/abstract=3792772

54. van der Meijden SL, Wang Y, Arbous MS, Geerts BF, Steyerberg EW, Hernandez-Boussard T. Navigating fairness in AI-based prediction models: Theoretical constructs and practical applications. medRxiv. 2025. Available from: http://dx.doi.org/10.1101/2025.03.24.25324500

55. Foulds JR, Islam R, Keya KN, Pan S. An intersectional definition of fairness. 2020 IEEE 36th International Conference on Data Engineering (ICDE). IEEE; 2020. p. 1918–21. Available from: https://ieeexplore.ieee.org/document/9101635

56. Cui S, Pan W, Zhang C, Wang F. Bipartite ranking fairness through a model agnostic ordering adjustment. IEEE Transactions on Pattern Analysis and Machine Intelligence. 2023. Available from: https://ieeexplore.ieee.org/document/10169084

57. Nguyen S, Wang A, Montillo A. Fairness-enhancing mixed effects deep learning improves fairness on in- and out-of-distribution clustered (non-iid) data. arXiv [cs.LG]. 2023. Available from: http://arxiv.org/abs/2310.03146

58. Keya KN, Islam R, Pan S, Stockwell I, Foulds JR. Equitable allocation of healthcare resources with fair cox models. arXiv. 2020. Available from: https://arxiv.org/abs/2010.06820

59. Rahman MM, Purushotham S. Fair and interpretable models for survival analysis. Proceedings of the 28th ACM SIGKDD Conference on Knowledge Discovery and Data Mining. New York, NY, USA: ACM; 2022. p. 1452–62. Available from: https://dl.acm.org/doi/pdf/10.1145/3534678.3539259

60. Zemel R, Wu Y, Swersky K, Pitassi T, Dwork C. Learning fair representations. International conference on machine learning. PMLR; 2013. p. 325–33. Available from: https://proceedings.mlr.press/v28/zemel13

61. Jiang Z, Han X, Fan C, Yang F, Mostafavi A, Hu X. Generalized demographic parity for group fairness. International Conference on Learning Representations. 2022. Available from: https://openreview.net/pdf?id=YigKlMJwjye

62. Feldman M, Friedler SA, Moeller J, Scheidegger C, Venkatasubramanian S. Certifying and removing disparate impact. In: proceedings of the 21th ACM SIGKDD international conference on knowledge discovery and data mining. 2015. p. 259–68. Available from: https://dl.acm.org/doi/10.1145/2783258.2783311

63. Coston A, Ramamurthy KN, Wei D, Varshney KR, Speakman S, Mustahsan Z, et al. Fair transfer learning with missing protected attributes. Proceedings of the 2019 AAAI/ACM Conference on AI, Ethics, and Society. 2019. p. 91–8. Available from: https://dl.acm.org/doi/10.1145/3306618.3314236

64. Cheng V, Suriyakumar VM, Dullerud N, Joshi S, Ghassemi M. Can you fake it until you make it? impacts of differentially private synthetic data on downstream classification fairness. Proceedings of the 2021 ACM Conference on Fairness, Accountability, and Transparency. 2021. p. 149–60. Available from: https://dl.acm.org/doi/10.1145/3442188.3445879

65. Pfohl S, Marafino B, Coulet A, Rodriguez F, Palaniappan L, Shah NH. Creating fair models of atherosclerotic cardiovascular disease risk. Proceedings of the 2019 AAAI/ACM Conference on AI, Ethics, and Society. 2019. p. 271–8. Available from: https://dl.acm.org/doi/10.1145/3306618.3314278

66. Kallus N, Zhou A. The fairness of risk scores beyond classification: Bipartite ranking and the xauc metric. Advances in neural information processing systems. 2019;32. Available from: https://dl.acm.org/doi/10.5555/3454287.3454596

67. Meissen F, Breuer S, Knolle M, Buyx A, Müller R, Kaissis G, et al. (Predictable) performance bias in unsupervised anomaly detection. Ebiomedicine. 2024;101. Available from: https://www.sciencedirect.com/science/article/pii/S2352396424000379

68. Luo Y, Tian Y, Shi M, Elze T, Wang M. Eye fairness: A large-scale 3d imaging dataset for equitable eye diseases screening and fair identity scaling. arXiv. 2023. Available from: https://arxiv.org/abs/2310.02492

69. Anderson JW, Shaikh N, Visweswaran S. Measuring and Reducing Racial Bias in a Pediatric Urinary Tract Infection Model. AMIA Summits on Translational Science Proceedings. 2024;2024:488. Available from: https://pubmed.ncbi.nlm.nih.gov/37790354/

70. Röösli E, Bozkurt S, Hernandez-Boussard T. Peeking into a black box, the fairness and generalizability of a MIMIC-III benchmarking model. Scientific Data. 2022;9(1):24. Available from: https://www.nature.com/articles/s41597-021-01110-7

71. Zhang W, Weiss JC. Longitudinal fairness with censorship. proceedings of the AAAI conference on artificial

intelligence. 2022. p. 12235–43. Available from: https://doi.org/10.1609/aaai.v36i11.21484

72. Pfohl S, Xu Y, Foryciarz A, Ignatiadis N, Genkins J, Shah N. Net benefit, calibration, threshold selection, and training objectives for algorithmic fairness in healthcare. Proceedings of the 2022 ACM Conference on Fairness, Accountability, and Transparency. 2022. p. 1039–52. Available from: https://dl.acm.org/doi/10.1145/3531146.3533166

73. Kleinberg J, Mullainathan S, Raghavan M. Inherent trade-offs in the fair determination of risk scores. arXiv. 2016. Available from: https://dl.acm.org/doi/10.1145/3603195.3603197

74. Corbett-Davies S, Pierson E, Feller A, Goel S, Huq A. Algorithmic decision making and the cost of fairness. Proceedings of the 23rd acm SIGKKD international conference on knowledge discovery and data mining. 2017. p. 797–806. Available from: https://dl.acm.org/doi/10.1145/3097983.3098095

75. Jung C, Kannan S, Lee C, Pai M, Roth A, Vohra R. Fair prediction with endogenous behavior. Proceedings of the 21st ACM Conference on Economics and Computation. 2020. p. 677–8. Available from: https://dl.acm.org/doi/abs/10.1145/3391403.3399473

76. Alam MAU. Ai-fairness towards activity recognition of older adults. MobiQuitous 2020-17th EAI International Conference on Mobile and Ubiquitous Systems: Computing, Networking and Services. 2020. p. 108–17. Available from: https://dl.acm.org/doi/10.1145/3448891.3448943

77. Wang Y, Pillai M, Zhao Y, Curtin C, Hernandez-Boussard T. FairEHR-CLP: Towards Fairness-Aware Clinical Predictions with Contrastive Learning in Multimodal Electronic Health Records. arXiv. 2024. Available from: https://arxiv.org/abs/2402.00955

78. DiCiccio C, Hsu B, Yu Y, Nandy P, Basu K. Detection and mitigation of algorithmic bias via predictive parity. In: Proceedings of the 2023 ACM Conference on Fairness, Accountability, and Transparency. 2023. p. 1801–16. Available from: https://dl.acm.org/doi/10.1145/3593013.3594117

79. Beutel A, Chen J, Doshi T, Qian H, Woodruff A, Luu C, et al. Putting fairness principles into practice: Challenges, metrics, and improvements. Proceedings of the 2019 AAAI/ACM Conference on AI, Ethics, and Society. 2019. p. 453–9. Available from: https://dl.acm.org/doi/10.1145/3306618.3314234

80. Berk R, Heidari H, Jabbari S, Kearns M, Roth A. Fairness in criminal justice risk assessments: The state of the art. arXiv [stat.ML]. 2017. Available from: http://arxiv.org/abs/1703.09207

81. Xiao Y, Lim S, Pollard TJ, Ghassemi M. In the name of fairness: assessing the bias in clinical record de-identification. Proceedings of the 2023 ACM Conference on Fairness, Accountability, and Transparency. 2023. p. 123–37. Available from: https://dl.acm.org/doi/10.1145/3593013.3593982

82. Riad R, Denais M, de Gennes M, Lesage A, Oustric V, Cao XN, et al. Automated speech analysis for risk detection of depression, anxiety, insomnia, and fatigue: Algorithm Development and Validation Study. Journal of Medical Internet Research. 2024;26:e58572. Available from: https://www.jmir.org/2024/1/e58572/

83. Diana E, Gill W, Kearns M, Kenthapadi K, Roth A. Minimax group fairness: Algorithms and experiments. Proceedings of the 2021 AAAI/ACM Conference on AI, Ethics, and Society. 2021. p. 66–76. Available from: https://dl.acm.org/doi/10.1145/3461702.3462523

84. Poulain R, Bin Tarek MF, Beheshti R. Improving fairness in ai models on electronic health records: The case for federated learning methods. Proceedings of the 2023 ACM conference on fairness, accountability, and transparency. 2023. p. 1599–608. Available from: https://dl.acm.org/doi/10.1145/3593013.3594102

85. Speicher T, Heidari H, Grgic-Hlaca N, Gummadi KP, Singla A, Weller A, et al. A unified approach to quantifying algorithmic unfairness: Measuring individual &group unfairness via inequality indices. Proceedings of the 24th ACM SIGKDD international conference on knowledge discovery & data mining. 2018. p. 2239–48. Available from: https://dl.acm.org/doi/10.1145/3219819.3220046

86. Schaekermann M, Spitz T, Pyles M, Cole-Lewis H, Wulczyn E, Pfohl SR, et al. Health equity assessment of machine learning performance (HEAL): a framework and dermatology AI model case study. EClinicalMedicine. 2024;70. Available from: https://www.thelancet.com/journals/eclinm/article/PIIS2589-5370(24)00058-0/fulltext

87. Chen IY, Szolovits P, Ghassemi M. Can AI help reduce disparities in general medical and mental health care? AMA journal of ethics. 2019;21(2):167–79. Available from: https://pubmed.ncbi.nlm.nih.gov/30794127/

88. Chicco D, Jurman G. The advantages of the Matthews correlation coefficient (MCC) over F1 score and accuracy

in binary classification evaluation. BMC genomics. 2020;21:1–13. Available from: https://bmcgenomics.biomedcentral.com/articles/10.1186/s12864-019-6413-7

89. Benitez-Aurioles J, Joules A, Brusini I, Peek N, Sperrin M. Understanding algorithmic fairness for clinical prediction in terms of subgroup net benefit and health equity. arXiv [stat.AP]. 2024. Available from: http://arxiv.org/abs/2412.07879

90. Mittelstadt B, Wachter S, Russell C. The Unfairness of Fair Machine Learning: Levelling down and strict egalitarianism by default. Available from: https://papers.ssrn.com/abstract=4331652

91. Watkins EA, Chen J. The four-fifths rule is not disparate impact: A woeful tale of epistemic trespassing in algorithmic fairness. The 2024 ACM Conference on Fairness, Accountability, and Transparency. New York, NY, USA: ACM; 2024. Available from: http://dx.doi.org/10.1145/3630106.3658938

92. Wachter S, Mittelstadt B, Russell C. Why fairness cannot be automated: Bridging the gap between EU non-discrimination law and AI. SSRN Electron J. 2020 Mar 3; Available from: https://papers.ssrn.com/abstract=3547922

93. McDermott MBA, Zhang H, Hansen LH, Angelotti G, Gallifant J. A closer look at AUROC and AUPRC under class imbalance. arXiv [cs.LG]. 2024. Available from: http://arxiv.org/abs/2401.06091

94. Chouldechova A. Fair prediction with disparate impact: A study of bias in recidivism prediction instruments. Big data. 2017;5(2):153–63. Available from: https://www.semanticscholar.org/paper/Fair-prediction-with-disparate-impact%3A-A-study-of-Chouldechova/37f5d47019f467c74acff22a38ffd4b98bdcb5d4

95. Błasiok J, Nakkiran P. Smooth ECE: Principled reliability diagrams via kernel smoothing. arXiv [cs.LG]. 2023. Available from: http://arxiv.org/abs/2309.12236

96. Nam RK, Kattan MW, Chin JL, Trachtenberg J, Singal R, Rendon R, et al. Prospective multi-institutional study evaluating the performance of prostate cancer risk calculators. J Clin Oncol. 2011 Aug 1;29(22):2959–64. Available from: https://ascopubs.org/doi/10.1200/JCO.2010.32.6371

97. Vickers AJ, van Calster B, Steyerberg EW. A simple, step-by-step guide to interpreting decision curve analysis. Diagn Progn Res. 2019 Oct 4;3(1):18. Available from: https://diagnprognres.biomedcentral.com/articles/10.1186/s41512-019-0064-7

98. McCradden MD, Joshi S, Mazwi M, Anderson JA. Ethical limitations of algorithmic fairness solutions in health care machine learning. The Lancet Digital Health. 2020;2(5):e221–3. Available from: https://www.thelancet.com/journals/landig/article/PIIS2589-7500(20)30065-0/fulltext

99. Van Calster B, McLernon DJ, van Smeden M, Wynants L, Steyerberg EW, Topic Group "Evaluating diagnostic tests and prediction models" of the STRATOS initiative. Calibration: the Achilles heel of predictive analytics. BMC Med. 2019 Dec 16;17(1):230. Available from: https://bmcmedicine.biomedcentral.com/articles/10.1186/s12916-019-1466-7

100. Cheng HF, Stapleton L, Wang R, Bullock P, Chouldechova A, Wu ZSS, et al. Soliciting stakeholders' fairness notions in child maltreatment predictive systems. Proceedings of the 2021 CHI Conference on Human Factors in Computing Systems. New York, NY, USA: ACM; 2021. Available from: http://dx.doi.org/10.1145/3411764.3445308

101. Chien I, Deliu N, Turner RE, Weller A, Villar SS, Kilbertus N. Multi-disciplinary fairness considerations in machine learning for clinical trials. arXiv [cs.LG]. 2022. Available from: http://arxiv.org/abs/2205.08875

102. Tal E. Target specification bias, counterfactual prediction, and algorithmic fairness in healthcare. Proceedings of the 2023 AAAI/ACM Conference on AI, Ethics, and Society. New York, NY, USA: ACM; 2023. p. 312–21. Available from: http://dx.doi.org/10.1145/3600211.3604678

103. Jun I, Ser SE, Cohen SA, Xu J, Lucero RJ, Bian J, et al. Quantifying health outcome disparity in invasive Methicillin-Resistant Staphylococcus aureus infection using fairness algorithms on real-world data. Pac Symp Biocomput. 2024;29:419–32. Available from: https://pubmed.ncbi.nlm.nih.gov/38160296/

104. Zhou R, Liu Y, Xia W, Guo Y, Huang Z, Gan H, et al. JoCoRank: Joint correlation learning with ranking similarity regularization for imbalanced fetal brain age regression. Comput Biol Med. 2024 Mar;171(108111):108111. Available from: http://dx.doi.org/10.1016/j.compbiomed.2024.108111

105. Crenshaw K. Demarginalizing the Intersection of Race and Sex: A Black Feminist Critique of Antidiscrimination

Doctrine, Feminist Theory and Antiracist Politics. Available from: https://chicagounbound.uchicago.edu/uclf/vol1989/iss1/8/

106. Gohar U, Cheng L. A survey on intersectional fairness in Machine Learning: Notions, mitigation, and challenges. arXiv [cs.LG]. 2023. Available from: https://www.ijcai.org/proceedings/2023/0742.pdf

107. Nielsen MW, Gissi E, Heidari S, Horton R, Nadeau KC, Ngila D, et al. Intersectional analysis for science and technology. Nature. 2025 Apr 9;640(8058):329–37. Available from: http://dx.doi.org/10.1038/s41586-025-08774-w

108. Vickers AJ, Elkin EB. Decision curve analysis: a novel method for evaluating prediction models. Med Decis Making. 2006 Nov;26(6):565–74. Available from: https://journals.sagepub.com/doi/10.1177/0272989X06295361

109. Riley RD, Collins GS. Stability of clinical prediction models developed using statistical or machine learning methods. Biom J. 2023 Dec;65(8):e2200302. Available from: http://dx.doi.org/10.1002/bimj.202200302

110. Davis SE, Dorn C, Park DJ, Matheny ME. Emerging algorithmic bias: fairness drift as the next dimension of model maintenance and sustainability. J Am Med Inform Assoc. 2025 Mar 13;ocaf039. Available from: https://academic.oup.com/jamia/article/32/5/845/8074959

111. Riley RD, Collins GS, Whittle R, Archer L, Snell KIE, Dhiman P, et al. A decomposition of Fisher's information to inform sample size for developing fair and precise clinical prediction models -- part 1: binary outcomes. arXiv [stat.ME]. 2024. Available from: http://arxiv.org/abs/2407.09293

112. Riley RD, Collins GS, Archer L, Whittle R, Legha A, Kirton L, et al. A decomposition of Fisher's information to inform sample size for developing fair and precise clinical prediction models -- Part 2: time-to-event outcomes. arXiv [stat.ME]. 2025. Available from: http://arxiv.org/abs/2501.14482

113. Riley RD, Collins GS, Kirton L, Snell KI, Ensor J, Whittle R, et al. Uncertainty of risk estimates from clinical prediction models: rationale, challenges, and approaches. BMJ. 2025 Feb 13;388:e080749. Available from: http://dx.doi.org/10.1136/bmj-2024-080749

114. Oxford English Dictionary. intersectionality, Oxford University Press; 2023 Jul. Available from: https://doi.org/10.1093/OED/7276681610

115. Movva R, Shanmugam D, Hou K, Pathak P, Guttag J, Garg N, et al. Coarse race data conceals disparities in clinical risk score performance. arXiv [cs.CY]. 2023. Available from: http://arxiv.org/abs/2304.09270

116. Herlihy C, Truong K, Chouldechova A, Dudik M. A structured regression approach for evaluating model performance across intersectional subgroups. arXiv [cs.LG]. 2024. Available from: http://arxiv.org/abs/2401.14893

117. Thomassen D, le Cessie S, van Houwelingen HC, Steyerberg EW. Effective sample size: A measure of individual uncertainty in predictions. Stat Med. 2024 Mar 30;43(7):1384–96. Available from: http://dx.doi.org/10.1002/sim.10018

118. Efthimiou O, Seo M, Chalkou K, Debray T, Egger M, Salanti G. Developing clinical prediction models: a step-by-step guide. BMJ. 2024 Sep 3;386:e078276. Available from: https://www.bmj.com/content/386/bmj-2023-078276.abstract

119. Oxford English Dictionary. Fairness, N., Sense 6. Oxford University Press; 2024 Dec. Available from: https://doi.org/10.1093/OED/8693190878

120. Oxford English Dictionary. justice, n. meanings, etymology and more. Oxford University Press; 2025 Jun. Available from: https://doi.org/10.1093/OED/1055369584

121. Beigang F. Reconciling algorithmic fairness criteria. Philos Public Aff. 2023 Apr 1;51(2):166–90. Available from: http://dx.doi.org/10.1111/papa.12233

122. Ravindranath R, Stein JD, Hernandez-Boussard T, Fisher AC, Wang SY, SOURCE Consortium. The impact of race, ethnicity, and sex on fairness in artificial intelligence for glaucoma prediction models. Ophthalmol Sci. 2025 Jan 1;5(1):100596. Available from: http://dx.doi.org/10.1016/j.xops.2024.100596

123. Byrd TF 4th, Southwell B, Ravishankar A, Tran T, Kc A, Phelan T, et al. Validation of a proprietary Deterioration Index model and performance in hospitalized adults. JAMA Netw Open. 2023 Jul 3;6(7):e2324176. Available from: https://jamanetwork.com/journals/jamanetworkopen/fullarticle/2807550

124. Arnett DK, Blumenthal RS, Albert MA, Buroker AB, Goldberger ZD, Hahn EJ, et al. 2019 ACC/AHA guideline on the primary prevention of cardiovascular disease: A report of the American college of cardiology/American heart association task force on clinical practice guidelines. J Am Coll Cardiol. 2019 Sep 10;74(10):e177–232. Available from: https://www.jacc.org/doi/10.1016/j.jacc.2019.03.010

125. Johnson AEW, Bulgarelli L, Shen L, Gayles A, Shammout A, Horng S, et al. MIMIC-IV, a freely accessible electronic health record dataset. Sci Data. 2023 Jan 3;10(1):1. Available from: http://dx.doi.org/10.1038/s41597-022-01899-x

126. Yang MY, Kwak GH, Pollard T, Celi LA, Ghassemi M. Evaluating the Impact of Social Determinants on Health Prediction in the Intensive Care Unit. Proceedings of the 2023 AAAI/ACM Conference on AI, Ethics, and Society. 2023. p. 333–50. Available from: https://dl.acm.org/doi/10.1145/3600211.3604719

127. Barocas S, Hardt M, Narayanan A. Fairness and machine learning: Limitations and opportunities. MIT press; 2023. Available from: https://fairmlbook.org/

128. Rountree L, Lin YT, Liu C, Salvatore M, Admon A, Nallamothu BK, et al. Reporting of fairness metrics in clinical risk prediction models: A call for change. medRxiv. 2024. p. 2024.03.16.24304390. Available from: https://www.medrxiv.org/content/10.1101/2024.03.16.24304390v1.abstract

129. Garg P, Villasenor J, Foggo V. Fairness Metrics: A Comparative Analysis. arXiv [cs.CY]. 2020. Available from: http://arxiv.org/abs/2001.07864

130. Bellamy RKE, Dey K, Hind M, Hoffman SC, Houde S, Kannan K, et al. AI fairness 360: An extensible toolkit for detecting, understanding, and mitigating unwanted algorithmic bias. arXiv [cs.AI]. 2018. Available from: http://arxiv.org/abs/1810.01943

131. Liu M, Ning Y, Teixayavong S, Mertens M, Xu J, Ting DSW, et al. A translational perspective towards clinical AI fairness. NPJ Digit Med. 2023 Sep 14;6(1):172. Available from: https://www.nature.com/articles/s41746-023-00918-4

132. Castelnovo A, Crupi R, Greco G, Regoli D, Penco IG, Cosentini AC. A clarification of the nuances in the fairness metrics landscape. Sci Rep. 2022 Mar 10;12(1):4209. Available from: https://www.nature.com/articles/s41598-022-07939-1

133. Wan M, Zha D, Liu N, Zou N. In-processing modeling techniques for machine learning fairness: A survey. ACM Trans Knowl Discov Data. 2022 Jul 30; Available from: https://dl.acm.org/doi/pdf/10.1145/3551390

134. Mitchell S, Potash E, Barocas S, D'Amour A, Lum K. Prediction-based decisions and fairness: A catalogue of choices, assumptions, and definitions. arXiv [stat.AP]. 2018. Available from: http://arxiv.org/abs/1811.07867

135. Huang Y, Guo J, Chen WH, Lin HY, Tang H, Wang F, et al. A scoping review of fair machine learning techniques when using real-world data. Journal of Biomedical Informatics. 2024;104622. Available from: https://doi.org/10.1016/j.jbi.2024.104622

136. Mitchell S, Potash E, Barocas S, D'Amour A, Lum K. Algorithmic fairness: Choices, assumptions, and definitions. Annu Rev Stat Appl. 2021 Mar 7;8(1):141–63. Available from: https://www.annualreviews.org/content/journals/10.1146/annurev-statistics-042720-125902

137. Makhlouf K, Zhioua S, Palamidessi C. On the applicability of machine Learning fairness notions. SIGKDD Explor. 2021 May 26;23(1):14–23. Available from: https://dl.acm.org/doi/10.1145/3468507.3468511

138. Barr CJS, Erdelyi O, Docherty PD, Grace RC. A review of fairness and A practical guide to selecting context-appropriate fairness metrics in machine learning. arXiv [cs.AI]. 2024. Available from: http://arxiv.org/abs/2411.06624
